# Supplementary material for: Switchable synthesis of natural-product-like lawsones and indenopyrazoles through regioselective ring-expansion of indantrione
Source: Commun Chem. 2023 Jan 18;6:17. doi: 10.1038/s42004-022-00807-z (PMC9849474; doi:10.1038/s42004-022-00807-z)
Supplement: Supplementary file 2 — Supplementary Information [file 42004_2022_807_MOESM2_ESM.pdf]

Support Information

Switchable Synthesis of Natural-Product-Like Lawsones and Indenopyrazoles through Regioselective Ring-Expansion of Indantrione

Bingwei Hu[1†], Wenxin Yan[2†], Peiyun Jiang[1†], Ling Jiang[1], Xu Yuan[1], Jun Lin[1], Yinchun Jiao[2]* and Yi Jin[1]*

[1]Key Laboratory of Medicinal for Natural Resource, Ministry of Education and Yunnan Province, School of Pharmacy, Yunnan University, Kunming 650091, China.

[2]School of Chemistry and Chemical Engineering; Key Laboratory of Theoretical Organic Chemistry and Functional Molecular, Ministry of Education, Hunan University of Science and Technology, Xiangtan, 411201, P. R. China.

[†]These authors contributed equally: Bingwei Hu, Wenxin Yan, Peiyun Jiang

*corresponding authors' email: yinchunjiao@hnust.edu.cn, jinyi@ynu.edu.cn

Table of Contents

Supplementary Note 1. General information S2

Supplementary Note 2. Spectroscopic Data S2-S38

Supplementary Method 1. Gram-scale Reactions S39-S40

Supplementary Method 2. Synthetic Transformations S40-S43

Supplementary Note 3. X-ray Structure and Data of Products S44-S49

Supplementary Note 1

General information

All chemicals and reagents were used of commercial grade and were used without no further purification. The reactions were monitored by thin-layer chromatography (TLC) using silica gel GF254. Column chromatography was performed with 200–300 mesh silica gel. All yields refer to isolated products after purification. The intermediates and the products synthesized were fully characterized by spectroscopic data. The NMR spectra were recorded on Bruker DRX-600 (1H: 600 MHz, 13C: 151 MHz) using Chloroform-*d* and DMSO-d_6 as solvents. The following abbreviation were used to explain the multiplicities: (s) = singlet, (d) = doublet, (t) = triplet, (q) = quartet, (sept) = septuplet, (dd) = double doublet, (dt) = double triplet, (dq) = double quartet, (ddd) = double-double doublet, (m) = multiplet; Chemical shifts (δ) are expressed in parts per million (ppm) and J values are given in hertz (Hz). IR spectra were recorded on an FT-IR Thermo Nicolet Avatar 360 using a KBr pellet. HRMS was performed on an Agilent LC/MSD TOF instrument. The melting points were measured by the XT-4A melting point apparatus without correction.

Supplementary Note 2

Spectroscopic Data of 4a-4am, 5a-5ae, 5o-d_3

Spectroscopic Data of **4a**

2-hydroxy-3-phenylnaphthalene-1,4-dione

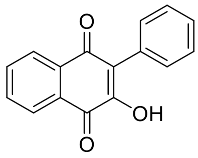

Red solid; Mp: 143.7-144.3 ^{o}C; 230 mg, yield: 92%;

^{1}**H NMR** (500 MHz, Chloroform-*d*) δ 8.09 (dd, *J* = 26.9, 7.6 Hz, 2H, ArH), 7.69 (dt, *J* = 36.9, 7.7 Hz, 2H, ArH), 7.56 (s, 1H, OH), 7.41 (dd, *J* = 21.6, 7.5 Hz, 4H, ArH), 7.32 (t, *J* = 7.4 Hz, 1H, ArH).

^{13}C NMR (126 MHz, Chloroform-*d*) δ 182.69, 180.83, 151.22, 134.26, 132.11, 131.81, 129.62, 128.95, 128.29, 127.63, 126.90, 126.26, 125.10, 121.14;

HRMS (TOF ES+): m/z calcd for $C_{16}H_{10}O_3$ $[M+H]^+$, 251.0703; found, 251.0704.

Spectroscopic Data of **4b**

2-(4-fluorophenyl)-3-hydroxynaphthalene-1,4-dione

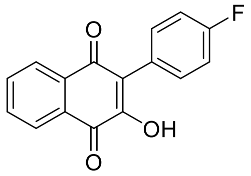

Yellow solid; Mp: 186.1-186.7 ^{o}C; 257 mg, yield: 96%;

^{1}H NMR (500 MHz, Chloroform-*d*) δ 8.10 (dd, *J* = 23.2, 7.6 Hz, 2H, ArH), 7.71 (dt, *J* = 36.3, 7.5 Hz, 2H, ArH), 7.57 (s, 1H, OH), 7.45 (t, *J* = 6.8 Hz, 2H, ArH), 7.08 (t, *J* = 8.6 Hz, 2H, ArH).

^{13}C NMR (126 MHz, Chloroform-*d*) δ 182.65, 180.69, 161.73 (d, *J* = 248.9 Hz), 151.20, 134.35, 132.22, 131.77, 131.71, 131.65, 128.25, 126.31, 125.18, 124.80 (d, *J* = 3.2 Hz), 120.10, 114.11, 113.94;

^{19}F NMR (471 MHz, Chloroform-*d*) δ -112.27.

HRMS (TOF ES+): m/z calcd for $C_{16}H_9FO_3$ $[M+H]^+$, 269.0608; found, 269.0610.

Spectroscopic Data of **4c**

2-(4-chlorophenyl)-3-hydroxynaphthalene-1,4-dione

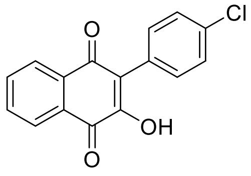

Yellow solid; Mp: 186.8-187.3 ^{o}C; 267 mg, yield: 94%;

^{1}H NMR (500 MHz, Chloroform-*d*) δ 8.10 (dd, *J* = 22.8, 7.7 Hz, 2H, ArH), 7.75 (t, *J* = 7.6 Hz, 1H, ArH), 7.67 (t, *J* = 7.6 Hz, 1H, ArH), 7.60 (s, 1H, OH), 7.43 – 7.32 (m, 4H, ArH).

^{13}C NMR (126 MHz, Chloroform-*d*) δ 182.44, 180.60, 151.26, 134.39, 133.66, 132.25, 131.75, 131.11, 128.23, 127.34, 127.19, 126.33, 125.21, 119.91.

HRMS (TOF ES+): m/z calcd for $C_{16}H_9ClO_3$ $[M+H]^+$, 285.0313; found, 285.0319.

Spectroscopic Data of **4d**

2-(4-bromophenyl)-3-hydroxynaphthalene-1,4-dione

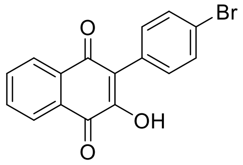

Red solid; Mp: 195.6-196.5 ^{o}C; 305 mg, yield: 93%;

^{1}H NMR (500 MHz, Chloroform-*d*) δ 8.10 (dd, *J* = 22.5, 7.7 Hz, 2H, ArH), 7.75 (t, *J* = 7.6 Hz, 1H, ArH), 7.67 (t, *J* = 7.5 Hz, 1H, ArH), 7.59 (s, 1H, OH), 7.51 (d, *J* = 8.1 Hz, 2H, ArH), 7.33 (d, *J* = 8.0 Hz, 2H, ArH).

^{13}C NMR (126 MHz, Chloroform-*d*) δ 182.36, 180.59, 151.22, 134.40, 132.26, 131.75, 131.36, 130.15, 128.23, 127.83, 126.33, 125.22, 122.01, 119.94.

HRMS (TOF ES+): m/z calcd for $C_{16}H_9BrO_3$ $[M+H]^+$, 328.9808; found, 328.9803.

Spectroscopic Data of **4e**

2-hydroxy-3-(p-tolyl)naphthalene-1,4-dione

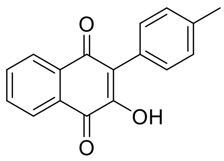

Yellow solid; Mp: 171.8-172.5 ^{o}C; 238 mg, yield: 90%;

^{1}H NMR (400 MHz, Chloroform-*d*) δ 8.08 (ddd, *J* = 22.9, 7.6, 1.3 Hz, 2H, ArH), 7.68 (dtd, *J* = 29.1, 7.5, 1.4 Hz, 2H, ArH), 7.34 (d, *J* = 8.1 Hz, 2H, ArH), 7.19 (d, *J* = 7.9 Hz, 2H, ArH), 2.32 (s, 3H, CH_3).

^{13}C NMR (101 MHz, Chloroform-*d*) δ 182.82, 180.81, 151.07, 137.67, 134.16, 132.06, 131.83, 129.52, 128.31, 127.68, 126.22, 125.93, 125.04, 121.22, 20.41.

HRMS (TOF ES+): m/z calcd for $C_{17}H_{12}O_3$ $[M+H]^+$, 265.0859; found, 265.0860.

Spectroscopic Data of **4f**

2-(4-ethylphenyl)-3-hydroxynaphthalene-1,4-dione

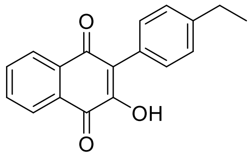

Yellow solid; Mp: 185.1-185.7 ^{o}C; 238 mg, yield: 86%;

¹H NMR (400 MHz, Chloroform-*d*) δ 8.09 (dd, *J* = 23.0, 7.6 Hz, 2H, ArH), 7.68 (dt, *J* = 29.2, 7.5 Hz, 2H, ArH), 7.53 (s, 1H, OH), 7.37 (d, *J* = 7.9 Hz, 2H, ArH), 7.22 (d, *J* = 7.8 Hz, 2H, ArH), 2.63 (q, *J* = 7.6 Hz, 2H, CH_2), 1.23 – 1.17 (m, 3H, CH_3).

¹³C NMR (101 MHz, Chloroform-*d*) δ 182.84, 180.83, 151.05, 143.88, 134.17, 132.06, 131.84, 129.60, 128.32, 126.49, 126.24, 126.14, 125.05, 121.23, 27.76, 14.29.

HRMS (TOF ES+): m/z calcd for $C_{18}H_{14}O_3$ $[M+H]^+$, 279.1016; found, 279.1018.

Spectroscopic Data of **4g**

2-([1,1'-biphenyl]-4-yl)-3-hydroxynaphthalene-1,4-dione

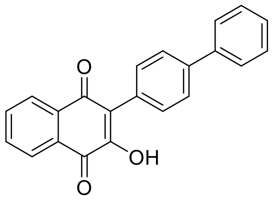

Yellow solid; Mp: 203.4-204.2 °C; 277 mg, yield: 85%;

¹H NMR (500 MHz, Chloroform-*d*) δ 8.10 (dd, *J* = 32.0, 7.7 Hz, 2H, ArH), 7.72 (t, *J* = 7.7 Hz, 1H, ArH), 7.68 – 7.49 (m, 8H, ArH and OH), 7.37 (t, *J* = 7.7 Hz, 2H, ArH), 7.28 (t, *J* = 7.5 Hz, 1H, ArH).

¹³C NMR (126 MHz, Chloroform-*d*) δ 182.74, 180.71, 151.20, 140.42, 139.70, 134.26, 132.15, 131.82, 130.13, 128.29, 127.89, 127.76, 126.48, 126.29, 126.17, 125.65, 125.12, 120.75.

HRMS (TOF ES+): m/z calcd for $C_{22}H_{14}O_3$ $[M+H]^+$, 327.1016; found, 327.1021.

Spectroscopic Data of **4h**

2-hydroxy-3-(4-methoxyphenyl)naphthalene-1,4-dione

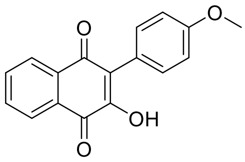

Red solid; Mp: 175.6-176.3 °C; 238 mg, yield: 85%;

¹H NMR (400 MHz, Chloroform-*d*) δ 8.09 (ddd, *J* = 23.0, 7.6, 1.4 Hz, 2H, ArH), 7.68 (dtd, *J* = 28.5, 7.5, 1.4 Hz, 2H, ArH), 7.48 – 7.39 (m, 2H, ArH), 6.96 – 6.88 (m, 2H, ArH), 3.78 (s, 3H, CH_3).

¹³C NMR (101 MHz, Chloroform-*d*) δ 182.99, 180.76, 158.81, 150.83, 134.11, 132.07, 131.85, 131.20, 128.34, 126.23, 125.01, 121.12, 120.87, 112.47, 54.27.

HRMS (TOF ES+): m/z calcd for $C_{17}H_{12}O_4$ $[M+Na]^+$, 303.0628; found, 303.0627.

Spectroscopic Data of **4i**

2-hydroxy-3-(4-phenoxyphenyl)naphthalene-1,4-dione

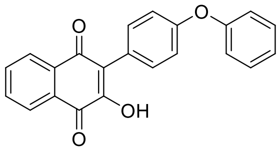

Red solid; Mp: 193.3-194.5 °C; 277 mg, yield: 81%;

^{1}H NMR (500 MHz, Chloroform-*d*) δ 8.12 (d, *J* = 7.6 Hz, 1H, ArH), 8.06 (d, *J* = 7.6 Hz, 1H, ArH), 7.75 – 7.69 (m, 1H, ArH), 7.65 (t, *J* = 7.7 Hz, 1H, ArH), 7.56 (s, 1H, OH), 7.44 (d, *J* = 8.3 Hz, 2H, ArH), 7.29 (t, *J* = 7.6 Hz, 2H, ArH), 7.10 – 6.96 (m, 5H, ArH).

^{13}C NMR (126 MHz, Chloroform-*d*) δ 182.83, 180.71, 156.84, 155.44, 151.02, 134.22, 132.14, 131.81, 131.38, 128.80, 128.29, 126.27, 125.09, 123.38, 122.76, 120.52, 118.64, 116.64.

HRMS (TOF ES+): m/z calcd for $C_{22}H_{14}O_4$ $[M+H]^+$, 343.0965; found, 343.0967.

Spectroscopic Data of **4j**

2-hydroxy-3-(4-(trifluoromethyl)phenyl)naphthalene-1,4-dione

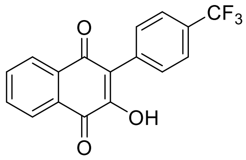

Red solid; Mp: 166.8-167.5 °C; 295 mg, yield: 93%;

^{1}H NMR (500 MHz, Chloroform-*d*) δ 8.12 (dd, *J* = 20.6, 7.6 Hz, 2H, ArH), 7.77 (t, *J* = 7.7 Hz, 1H, ArH), 7.70 (t, *J* = 7.7 Hz, 1H, ArH), 7.64 (d, *J* = 7.8 Hz, 3H, ArH and OH), 7.57 (d, *J* = 8.0 Hz, 2H, ArH).

^{13}C NMR (126 MHz, Chloroform-*d*) δ 182.23, 180.53, 151.56, 134.56, 132.66, 132.37, 131.67, 130.09, 129.42 (d, *J* = 32.6 Hz), 128.16, 126.38, 125.33, 123.82 (d, *J* = 3.8 Hz), 123.04 (d, *J* = 272.2 Hz), 119.60.
^{19}F NMR (471 MHz, Chloroform-*d*) δ -62.77.

HRMS (TOF ES+): m/z calcd for $C_{17}H_9F_3O_3$ $[M+H]^+$, 319.0577; found, 319.0582.

Spectroscopic Data of **4k**

2-hydroxy-3-(4-nitrophenyl)naphthalene-1,4-dione

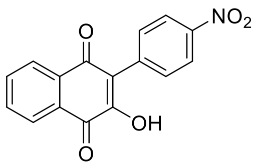

Red solid; Mp: 184.9-185.7 °C; 169 mg, yield: 84%;

^{1}H NMR (400 MHz, DMSO-d_6) δ 8.30 – 8.22 (m, 2H, ArH), 8.05 (ddd, *J* = 7.5, 4.1, 1.4 Hz, 2H, ArH), 7.85 (dtd, *J* = 23.2, 7.4, 1.4 Hz, 2H, ArH), 7.77 – 7.65 (m, 2H. ArH).

^{13}C NMR (101 MHz, DMSO-d_6) δ 182.90, 182.11, 161.47 – 153.40 (m), 146.60, 140.17, 135.28, 133.61, 132.83, 132.50, 130.63, 126.58, 126.16, 122.90, 119.92.

HRMS (TOF ES+): m/z calcd for $C_{16}H_9NO_5$ $[M+H]^+$, 296.0553; found, 296.0557.

Spectroscopic Data of **4l**

methyl 4-(3-hydroxy-1,4-dioxo-1,4-dihydronaphthalen-2-yl)benzoate

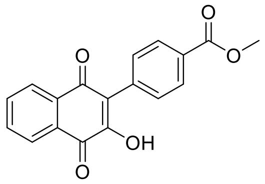

Red solid; Mp: 172.6-173.3 °C; 277 mg, yield: 90%;

^{1}H NMR (600 MHz, DMSO-d_6) δ 11.53 (s, 1H, OH), 8.14 – 8.02 (m, 4H, ArH), 7.91 (dt, *J* = 29.2, 7.5 Hz, 2H, ArH), 7.58 (d, *J* = 7.9 Hz, 2H, ArH), 3.93 (s, 3H, CH_3).

^{13}C NMR (151 MHz, DMSO-d_6) δ 183.62, 181.85, 166.61, 156.00, 137.26, 135.29, 133.80, 132.55, 131.60, 130.55, 129.00, 128.67, 126.59, 126.18, 121.43, 52.62.

HRMS (TOF ES+): m/z calcd for $C_{18}H_{12}O_5$ $[M+H]^+$, 309.0757; found, 309.0755.

Spectroscopic Data of **4m**

2-(3-fluorophenyl)-3-hydroxynaphthalene-1,4-dione

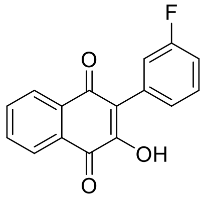

Yellow solid; Mp: 178.1-178.7 °C; 254 mg, yield: 95%;

^{1}H NMR (500 MHz, Chloroform-*d*) δ 8.11 (dd, *J* = 23.2, 7.7 Hz, 2H, ArH), 7.72 (dt, *J* = 37.9, 7.6 Hz, 2H, ArH), 7.60 (s, 1H, OH), 7.39 – 7.32 (m, 1H, ArH), 7.24 (d, *J* = 7.7 Hz, 1H, ArH), 7.18 (d, *J* = 5.5 Hz, 1H, ArH), 7.03 (td, *J* = 8.5, 2.6 Hz, 1H, ArH).

^{13}**C NMR** (126 MHz, Chloroform-*d*) δ 182.30, 180.66, 161.31 (d, *J* = 245.1 Hz), 151.39, 134.46, 132.26, 131.76, 130.92 (d, *J* = 8.6 Hz), 128.33 (d, *J* = 8.2 Hz), 128.20, 126.36, 125.48 (d, *J* = 3.1 Hz), 125.25, 119.80, 116.80 (d, *J* = 22.8 Hz), 114.57 (d, *J* = 20.9 Hz);

^{19}**F NMR** (471 MHz, Chloroform-*d*) δ -113.44.

HRMS (TOF ES+): m/z calcd for $C_{16}H_9FO_3$ $[M+H]^+$, 269.0608; found, 269.0610.

Spectroscopic Data of **4n**

2-(3-chlorophenyl)-3-hydroxynaphthalene-1,4-dione

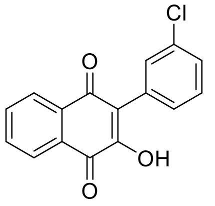

Red solid; Mp: 165.3-166.1 °C; 261 mg, yield: 92%;

^{1}**H NMR** (500 MHz, Chloroform-*d*) δ 8.11 (dd, *J* = 21.9, 7.7 Hz, 2H, ArH), 7.75 (t, *J* = 7.6 Hz, 1H, ArH), 7.68 (t, *J* = 7.6 Hz, 1H, ArH), 7.60 (s, 1H, OH), 7.45 (s, 1H, ArH), 7.32 (dd, *J* = 8.9, 4.4 Hz, 3H, ArH).

^{13}**C NMR** (126 MHz, Chloroform-*d*) δ 182.26, 180.60, 151.43, 134.47, 132.83, 132.28, 131.73, 130.66, 129.72, 128.20, 128.12, 127.89, 127.71, 126.35, 125.26, 119.69.

HRMS (TOF ES+): m/z calcd for $C_{16}H_9ClO_3$ $[M+Na]^+$, 307.0132; found, 307.0133.

Spectroscopic Data of **4o**

2-(3-bromophenyl)-3-hydroxynaphthalene-1,4-dione

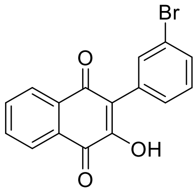

Red solid; Mp: 187.7-188.4 °C; 291 mg, yield: 89%;

^{1}**H NMR** (500 MHz, Chloroform-*d*) δ 8.11 (dd, *J* = 21.3, 7.7 Hz, 2H, ArH), 7.72 (dt, *J* = 37.6, 7.6 Hz, 2H, ArH), 7.66 – 7.49 (m, 2H, ArH and OH), 7.46 (d, *J* = 8.1 Hz, 1H, ArH), 7.38 (d, *J* = 7.8 Hz, 1H, ArH), 7.30 – 7.22 (m, 1H, ArH).

^{13}**C NMR** (126 MHz, Chloroform-*d*) δ 182.25, 180.60, 151.45, 134.48, 132.54, 132.29, 131.73, 130.94, 130.60, 128.39, 128.35, 128.20, 126.35, 125.27, 120.92, 119.60.

HRMS (TOF ES+): m/z calcd for $C_{16}H_9BrO_3$ $[M+H]^+$, 328.9808; found, 328.9810.

Spectroscopic Data of **4p**

2-hydroxy-3-(m-tolyl)naphthalene-1,4-dione

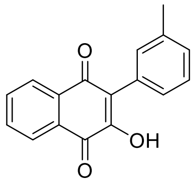

Yellow solid; Mp: 142.7-143.3 ^oC; 229 mg, yield: 87%;

^{1}H NMR (500 MHz, Chloroform-*d*) δ 8.10 (dd, *J* = 24.6, 7.6 Hz, 2H, ArH), 7.73 (t, *J* = 7.6 Hz, 1H, ArH), 7.66 (t, *J* = 7.5 Hz, 1H, ArH), 7.50 (s, 1H, OH), 7.31 – 7.20 (m, 3H, ArH), 7.14 (d, *J* = 7.7 Hz, 1H, ArH), 2.34 (s, 3H, ArH).

^{13}C NMR (126 MHz, Chloroform-*d*) δ 182.78, 180.87, 151.18, 136.50, 134.23, 132.09, 131.88, 130.12, 128.84, 128.50, 126.86, 126.63, 126.25, 125.10, 122.76, 121.44, 20.49.

HRMS (TOF ES+): m/z calcd for $C_{17}H_{12}O_3$ $[M+H]^+$, 265.0859; found, 265.0860.

Spectroscopic Data of **4q**

2-hydroxy-3-(3-methoxyphenyl)naphthalene-1,4-dione

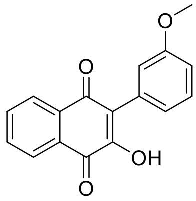

Red solid; Mp: 169.3-169.9 ^oC; 235 mg, yield: 84%;

^{1}H NMR (500 MHz, Chloroform-*d*) δ 8.11 (dd, *J* = 24.6, 7.7 Hz, 2H, ArH), 7.71 (dt, *J* = 36.9, 7.6 Hz, 2H, ArH), 7.52 (s, 1H, OH), 7.31 (t, *J* = 7.9 Hz, 1H, ArH), 7.05 – 6.96 (m, 2H, ArH), 6.92 – 6.85 (m, 1H, ArH), 3.77 (s, 3H, CH_3).

^{13}C NMR (126 MHz, Chloroform-*d*) δ 183.61, 181.85, 159.16, 152.27, 135.31, 133.14, 132.88, 131.18, 129.30, 128.95, 127.32, 126.16, 123.08, 122.11, 116.21, 114.50, 55.30.

HRMS (TOF ES+): m/z calcd for $C_{17}H_{12}O_4$ $[M+H]^+$, 281.0808; found, 281.0811.

Spectroscopic Data of **4r**

2-hydroxy-3-(3-(trifluoromethyl)phenyl)naphthalene-1,4-dione

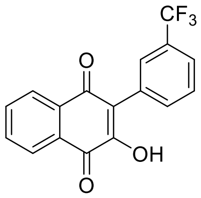

Red solid; Mp: 182.7-183.9 °C; 289 mg, yield: 91%;

^{1}H NMR (500 MHz, Chloroform-*d*) δ 8.13 (dd, *J* = 21.0, 7.6 Hz, 2H, ArH), 7.80 – 7.68 (m, 3H, ArH and OH), 7.61 (dd, *J* = 26.8, 9.0 Hz, 3H, ArH), 7.52 (d, *J* = 7.9 Hz, 1H, ArH).

^{13}C NMR (126 MHz, Chloroform-*d*) δ 182.24, 180.56, 151.55, 134.55, 133.07, 132.36, 131.70, 129.74, 128.20, 127.33, 126.67 (d, *J* = 4.1 Hz), 126.38, 125.32, 124.32 – 124.27 (m), 124.16, 119.49;

^{19}F NMR (471 MHz, Chloroform-*d*) δ -62.61;

HRMS (TOF ES+): m/z calcd for $C_{17}H_9F_3O_3$ $[M+H]^+$, 319.0577; found, 319.0577.

Spectroscopic Data of **4s**

3-(3-hydroxy-1,4-dioxo-1,4-dihydronaphthalen-2-yl)benzonitrile

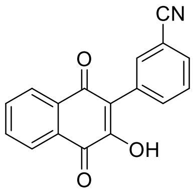

Red solid; Mp: 207.5-208.6 °C; 228 mg, yield: 85%;

^{1}H NMR (400 MHz, DMSO-*d*$_6$) δ 8.08 – 7.98 (m, 2H, ArH), 7.90 – 7.73 (m, 5H, ArH), 7.62 (t, *J* = 7.8 Hz, 1H, ArH).

^{13}C NMR (101 MHz, DMSO-*d*$_6$) δ 183.02, 182.23, 157.35, 136.18, 135.27, 134.71, 133.94, 133.61, 132.74, 131.33, 130.57, 129.19, 126.57, 126.14, 119.95, 119.36, 111.01.

HRMS (TOF ES+): m/z calcd for $C_{17}H_9NO_3$ $[M+H]^+$, 276.0655; found, 276.0658.

Spectroscopic Data of **4t**

2-hydroxy-3-(3-(4,4,5,5-tetramethyl-1,3,2-dioxaborolan-2-yl)phenyl)naphthalene-1,4-dione

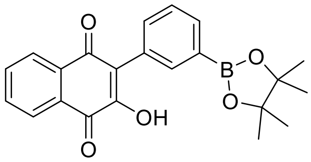

Red solid; Mp: 193.7-194.5 ^oC; 342 mg, yield: 91%;

^{1}H NMR (500 MHz, Chloroform-*d*) δ 8.09 (dd, *J* = 23.7, 7.8 Hz, 2H, ArH), 7.86 (s, 1H, OH), 7.75 (dd, *J* = 26.7, 7.6 Hz, 2H, ArH), 7.66 (t, *J* = 7.9 Hz, 1H, ArH), 7.51 (d, *J* = 6.7 Hz, 2H, ArH), 7.40 (t, *J* = 7.7 Hz, 1H, ArH), 1.27 (s, 12H, CH_3).

^{13}C NMR (126 MHz, Chloroform-*d*) δ 182.66, 180.80, 151.22, 135.80, 134.23, 134.04, 132.34, 132.09, 131.79, 128.38, 128.27, 126.34, 126.22, 125.11, 121.31, 82.83, 23.87.

HRMS (TOF ES+): m/z calcd for $C_{22}H_{21}BO_5$ $[M+H]^+$, 377.1555; found, 377.1561.

Spectroscopic Data of **4u**

2-(2-fluorophenyl)-3-hydroxynaphthalene-1,4-dione

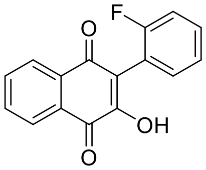

Red solid; Mp: 167.7-168.3 ^oC; 241 mg, yield: 90%;

^{1}H NMR (500 MHz, Chloroform-*d*) δ 8.12 (dd, *J* = 17.1, 7.7 Hz, 2H, ArH), 7.72 (dt, *J* = 35.7, 7.6 Hz, 2H, ArH), 7.54 (s, 1H, OH), 7.32 (dt, *J* = 22.6, 7.1 Hz, 2H, ArH), 7.19 – 7.07 (m, 2H, ArH).

^{13}C NMR (126 MHz, Chloroform-*d*) δ 181.68, 180.43, 159.16 (d, *J* = 249.6 Hz), 152.16, 134.39, 132.20, 131.89, 131.01 (d, *J* = 3.4 Hz), 129.73 (d, *J* = 8.3 Hz), 128.34, 126.32, 125.37, 122.74 (d, *J* = 3.5 Hz), 116.97 (d, *J* = 16.0 Hz), 116.63, 114.73 (d, *J* = 21.8 Hz);

^{19}F NMR (471 MHz, Chloroform-*d*) δ -110.61.

HRMS (TOF ES+): m/z calcd for $C_{16}H_9FO_3$ $[M+H]^+$, 269.0608; found, 269.0609.

Spectroscopic Data of **4v**

2-(2-chlorophenyl)-3-hydroxynaphthalene-1,4-dione

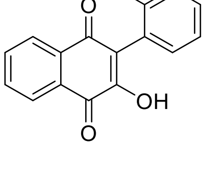

Red solid; Mp: 152.7-153.5 °C; 241 mg, yield: 85%;

^{1}H NMR (500 MHz, Chloroform-*d*) δ 8.12 (t, *J* = 8.8 Hz, 2H, ArH), 7.72 (dt, *J* = 34.9, 7.6 Hz, 2H, ArH), 7.60 – 7.35 (m, 2H, ArH and OH), 7.34 – 7.20 (m, 3H, ArH).

^{13}C NMR (126 MHz, Chloroform-*d*) δ 181.74, 180.58, 151.91, 134.41, 133.06, 132.21, 131.85, 130.57, 129.03, 128.68, 128.60, 128.38, 126.32, 125.54, 125.39, 120.05.

HRMS (TOF ES+): m/z calcd for $C_{16}H_9ClO_3$ $[M+H]^+$, 285.0313; found, 285.0315.

Spectroscopic Data of **4w**

2-(2-bromophenyl)-3-hydroxynaphthalene-1,4-dione

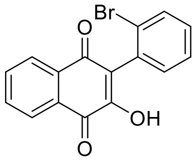

Red solid; Mp: 185.3-186.1 °C; 272 mg, yield: 83%;

^{1}H NMR (500 MHz, Chloroform-*d*) δ 8.12 (dd, *J* = 10.6, 7.8 Hz, 2H, ArH), 7.72 (dt, *J* = 35.2, 7.6 Hz, 2H, ArH), 7.62 (d, *J* = 8.0 Hz, 1H, ArH), 7.46 (d, *J* = 8.7 Hz, 1H, OH), 7.34 (t, *J* = 7.5 Hz, 1H, ArH), 7.23 – 7.17 (m, 2H, ArH).

^{13}C NMR (126 MHz, Chloroform-*d*) δ 182.75, 181.67, 152.73, 135.46, 133.26, 132.84, 132.78, 131.92, 131.52, 130.20, 129.42, 127.37, 127.22, 126.42, 123.96, 122.73.

HRMS (TOF ES+): m/z calcd for $C_{16}H_9BrO_3$ $[M+H]^+$, 328.9808; found, 328.9812.

Spectroscopic Data of **4x**

2-hydroxy-3-(o-tolyl)naphthalene-1,4-dione

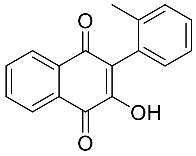

Red solid; Mp: 128.7-129.1 °C; 211 mg, yield: 80%;

^{1}H NMR (500 MHz, Chloroform-*d*) δ 8.11 (t, *J* = 7.2 Hz, 2H, ArH), 7.71 (dt, *J* = 32.8, 7.5 Hz, 2H, ArH), 7.40 (s, 1H, OH), 7.29 – 7.19 (m, 3H, ArH), 7.10 (d, *J* = 7.5 Hz, 1H, ArH), 2.13 (s, 3H, CH_3).

^{13}C NMR (126 MHz, Chloroform-*d*) δ 182.62, 180.76, 151.56, 135.98, 134.28, 132.12, 131.92, 129.16, 128.93, 128.80, 128.44, 127.82, 126.25, 125.26, 124.50, 122.12, 19.00.

HRMS (TOF ES+): m/z calcd for $C_{17}H_{12}O_3$ $[M+H]^+$, 265.0859; found, 265.0857.

Spectroscopic Data of **4y**

2-hydroxy-3-(2-methoxyphenyl)naphthalene-1,4-dione

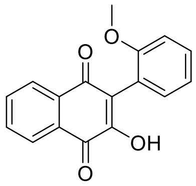

Red solid; Mp: 157.5-158.3 ^oC; 207 mg, yield: 74%;

^{1}H NMR (500 MHz, Chloroform-*d*) δ 8.09 (dd, *J* = 13.8, 7.7 Hz, 2H, ArH), 7.68 (dt, *J* = 34.1, 7.7 Hz, 2H, ArH), 7.37 (s, 1H, OH), 7.32 (d, *J* = 8.1 Hz, 1H, ArH), 7.21 – 7.12 (m, 1H, ArH), 7.02 – 6.89 (m, 2H, ArH), 3.71 (s, 3H, CH_3).

^{13}C NMR (126 MHz, Chloroform-*d*) δ 182.15, 180.76, 156.24, 151.80, 134.05, 132.14, 131.91, 130.28, 129.31, 128.54, 126.16, 125.17, 119.92, 119.46, 118.46, 110.33, 54.75.

HRMS (TOF ES+): m/z calcd for $C_{17}H_{12}O_4$ $[M+H]^+$, 281.0808; found, 281.0807.

Spectroscopic Data of **4z**

2-hydroxy-3-(2-(trifluoromethyl)phenyl)naphthalene-1,4-dione

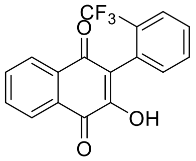

Red solid; Mp: 162.8-163.7 ^oC; 276 mg, yield: 87%;

^{1}H NMR (500 MHz, Chloroform-*d*) δ 8.10 (dd, *J* = 7.8, 3.5 Hz, 2H, ArH), 7.71 (dq, *J* = 20.1, 7.6 Hz, 3H, ArH), 7.56 (t, *J* = 7.5 Hz, 1H, ArH), 7.48 (d, *J* = 8.0 Hz, 1H, ArH), 7.38 (d, *J* = 16.0 Hz, 1H, OH), 7.23 (d, *J* = 7.6 Hz, 1H, ArH).

^{13}C NMR (126 MHz, Chloroform-*d*) δ 182.32, 180.39, 151.75, 134.46, 132.28, 131.68, 130.74, 130.31, 128.56 (d, *J* = 30.3 Hz), 128.32, 128.08, 127.85, 126.26, 125.49, 125.46, 122.86 (d, *J* = 273.8 Hz), 120.45.

^{19}F NMR (471 MHz, Chloroform-*d*) δ -60.76.

HRMS (TOF ES+): m/z calcd for $C_{17}H_9F_3O_3$ $[M+H]^+$, 319.0577; found, 319.0579.

Spectroscopic Data of **4aa**

2-(3,4-dimethylphenyl)-3-hydroxynaphthalene-1,4-dione

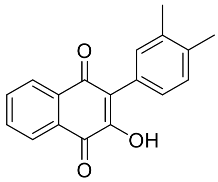

Red solid; Mp: 172.1-173.8 °C; 237 mg, yield: 85%;

^{1}H NMR (500 MHz, Chloroform-*d*) δ 8.08 (dd, *J* = 28.3, 7.6 Hz, 2H, ArH), 7.68 (dt, *J* = 35.9, 7.6 Hz, 2H, ArH), 7.49 (s, 1H, OH), 7.16 (q, *J* = 8.7, 7.0 Hz, 3H, ArH), 2.23 (d, *J* = 3.8 Hz, 6H, CH_3).

^{13}C NMR (126 MHz, Chloroform-*d*) δ 183.94, 181.88, 152.09, 137.47, 136.16, 135.16, 133.06, 132.92, 131.62, 129.39, 129.34, 128.09, 127.39, 127.25, 126.07, 122.53, 19.88, 19.73.

HRMS (TOF ES+): m/z calcd for $C_{18}H_{14}O_3$ $[M+H]^+$, 279.1016; found, 279.1012.

Spectroscopic Data of **4ab**

2-(3,5-dimethylphenyl)-3-hydroxynaphthalene-1,4-dione

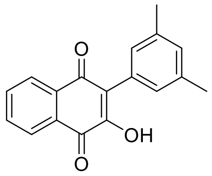

Yellow solid; Mp: 186.5-187.3 °C; 233 mg, yield: 84%;

^{1}H NMR (500 MHz, Chloroform-*d*) δ 8.08 (dd, *J* = 25.0, 7.6 Hz, 2H, ArH), 7.72 (t, *J* = 7.6 Hz, 1H, ArH), 7.64 (t, *J* = 7.5 Hz, 1H, ArH), 7.48 (s, 1H, OH), 7.01 (s, 2H, ArH), 6.96 (s, 1H, ArH), 2.29 (s, 6H, CH_3).

^{13}C NMR (126 MHz, Chloroform-*d*) δ 182.86, 180.85, 151.16, 136.42, 134.18, 132.05, 131.86, 129.50, 128.72, 128.32, 127.14, 126.21, 125.07, 121.69, 20.36.

HRMS (TOF ES+): m/z calcd for $C_{18}H_{14}O_3$ $[M+H]^+$, 279.1016; found, 279.1016.

Spectroscopic Data of **4ac**

2-(2,3-dichlorophenyl)-3-hydroxynaphthalene-1,4-dione

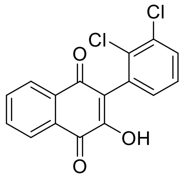

Yellow solid; Mp: 177.4-178.3 °C; 257 mg, yield: 81%;

^{1}H NMR (500 MHz, Chloroform-*d*) δ 8.11 (q, *J* = 5.3, 3.5 Hz, 2H, ArH), 7.73 (dt, *J* = 35.1, 7.7 Hz, 2H, ArH), 7.52 (s, 1H, OH), 7.46 (d, *J* = 7.7 Hz, 1H, ArH), 7.23 (t, *J* = 7.8 Hz, 1H, ArH), 7.13 (d, *J* = 7.2 Hz, 1H, ArH).

^{13}C NMR (126 MHz, Chloroform-*d*) δ 181.50, 180.44, 151.90, 134.54, 132.53, 132.34, 131.73, 131.50, 130.89, 129.74, 128.73, 128.31, 126.36, 126.17, 125.49, 119.75.

HRMS (TOF ES+): m/z calcd for $C_{16}H_8Cl_2O_3$ $[M+H]^+$, 318.9923; found, 318.9923.

Spectroscopic Data of **4ad**

2-hydroxy-3-(thiophen-3-yl)naphthalene-1,4-dione

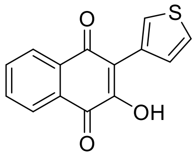

Yellow solid; Mp: 147.7-148.3 ^{o}C; 238 mg, yield: 93%;

^{1}H NMR (500 MHz, Chloroform-*d*) δ 8.12 (d, *J* = 7.7 Hz, 1H, ArH), 8.07 – 7.97 (m, 2H, ArH), 7.85 (s, 1H, OH), 7.76 – 7.60 (m, 3H, ArH), 7.31 (d, *J* = 3.2 Hz, 1H, ArH).

^{13}C NMR (126 MHz, Chloroform-*d*) δ 183.85, 181.69, 151.47, 135.24, 133.13, 133.04, 130.04, 129.75, 129.50, 129.13, 127.33, 126.02, 124.02, 116.99.

HRMS (TOF ES+): m/z calcd for $C_{14}H_8O_3S$ $[M+H]^+$, 257.0267; found, 257.0265.

Spectroscopic Data of **4ae**

3-hydroxy-[2,2'-binaphthalene]-1,4-dione

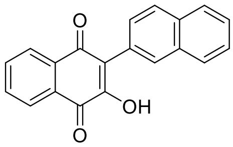

Red solid; Mp: 195.7-196.5 ^{o}C; 264 mg, yield: 88%;

^{1}H NMR (400 MHz, DMSO-*d*$_6$) δ 8.07 (ddd, *J* = 9.7, 7.4, 1.5 Hz, 2H, ArH), 7.97 – 7.91 (m, 4H, ArH), 7.91 – 7.81 (m, 2H, ArH), 7.59 – 7.48 (m, 3H, ArH).

^{13}C NMR (101 MHz, DMSO-*d*$_6$) δ 184.14, 182.00, 155.79, 135.24, 133.77, 132.88, 132.74, 132.57, 130.54, 130.40, 129.63, 129.13, 128.48, 127.93, 126.99, 126.80, 126.58, 126.49, 126.13, 122.55.

HRMS (TOF ES+): m/z calcd for $C_{20}H_{12}O_3$ $[M+Na]^+$, 323.0679; found, 323.0683.

Spectroscopic Data of **4af**

3'-hydroxy-[1,2'-binaphthalene]-1',4'-dione

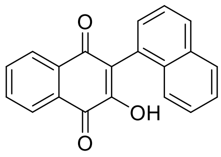

Red solid; Mp: 188.6.7-189.5 °C; 255 mg, yield: 85%;

^{1}H NMR (500 MHz, Chloroform-*d*) δ 8.10 (d, *J* = 7.4 Hz, 2H, ArH), 7.82 (dd, *J* = 13.4, 8.4 Hz, 2H, ArH), 7.68 (dt, *J* = 30.5, 7.8 Hz, 2H, ArH), 7.49 (dt, *J* = 15.4, 8.2 Hz, 3H, ArH and OH), 7.40 – 7.31 (m, 3H, ArH).

^{13}C NMR (126 MHz, Chloroform-*d*) δ 182.81, 180.53, 152.39, 134.33, 132.60, 132.20, 131.92, 130.32, 128.48, 128.24, 127.58, 127.22, 126.93, 126.32, 125.33, 125.18, 124.83, 124.33, 124.15, 120.97.

HRMS (TOF ES+): m/z calcd for $C_{20}H_{12}O_3$ $[M+H]^+$, 301.0859; found, 301.0863.

Spectroscopic Data of **4ag**

2-(2,3-dihydrobenzo[b][1,4]dioxin-6-yl)-3-hydroxynaphthalene-1,4-dione

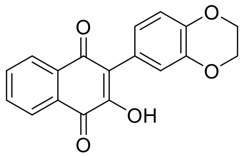

Red solid; Mp: 213.3-214.5 °C; 249 mg, yield: 81%;

^{1}H NMR (600 MHz, DMSO-*d*$_6$) δ 11.10 (s, 1H, OH), 8.08 (dd, *J* = 15.8, 7.6 Hz, 2H, ArH), 7.90 (dt, *J* = 28.4, 7.7 Hz, 2H, ArH), 6.98 – 6.88 (m, 3H, ArH), 4.33 (s, 4H, CH_2).

^{13}C NMR (151 MHz, DMSO-*d*$_6$) δ 184.06, 181.95, 155.20, 143.49, 142.88, 135.14, 133.67, 132.59, 130.46, 126.58, 126.01, 124.65, 124.52, 122.10, 120.05, 116.46, 64.71, 64.51.

HRMS (TOF ES+): m/z calcd for $C_{18}H_{12}O_5$ $[M+H]^+$, 309.0757; found, 309.0761.

Spectroscopic Data of **4ah**

2-(1,9-dihydropyren-4-yl)-3-hydroxynaphthalene-1,4-dione

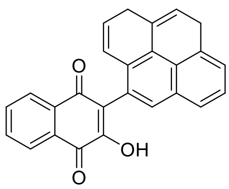

Red solid; Mp: 246.7-247.6 °C; 299 mg, yield: 80%;

^{1}H NMR (500 MHz, Chloroform-*d*) δ 8.12 (ddd, *J* = 31.0, 16.6, 7.7 Hz, 6H, ArH), 8.01 (s, 2H, ArH), 7.93 (dd, *J* = 13.0, 8.6 Hz, 2H, ArH), 7.84 (d, *J* = 7.8 Hz, 1H, ArH), 7.77 – 7.64 (m, 3H, ArH), 7.54 (s, 1H, OH).

^{13}C NMR (126 MHz, Chloroform-*d*) δ 183.04, 180.52, 152.48, 134.35, 132.23, 131.94, 130.75, 130.16, 129.78, 128.53, 128.28, 127.07, 126.99, 126.75, 126.38, 126.33, 125.34, 124.97, 124.48, 124.35, 124.10, 123.96, 123.82, 123.69, 123.49, 121.31.

HRMS (TOF ES+): m/z calcd for $C_{26}H_{14}O_3$ $[M+H]^+$, 375.1016; found, 375.1021.

Spectroscopic Data of **4ai**

2-hydroxy-3-(2-methylprop-1-en-1-yl)naphthalene-1,4-dione

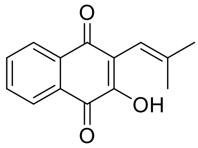

Red solid; Mp: 140.5-141.8 °C; 168 mg, yield: 74%;

^{1}H NMR (400 MHz, Chloroform-*d*) δ 8.04 (ddd, *J* = 14.1, 7.6, 1.4 Hz, 2H, ArH), 7.69 (td, *J* = 7.6, 1.4 Hz, 1H, ArH), 7.62 (td, *J* = 7.5, 1.4 Hz, 1H, ArH), 5.93 (p, *J* = 1.4 Hz, 1H, CH), 1.92 (s, 3H, CH_3), 1.61 (s, 3H, CH_3).

^{13}C NMR (101 MHz, Chloroform-*d*) δ 183.76, 180.59, 150.14, 142.59, 133.92, 131.98, 131.92, 128.53, 125.93, 125.08, 119.93, 112.64, 25.56, 20.71.

HRMS (TOF ES+): m/z calcd for $C_{14}H_{12}O_3$ $[M+H]^+$, 229.0859; found, 229.0860.

Spectroscopic Data of **4aj**

2-hydroxy-3-propylnaphthalene-1,4-dione

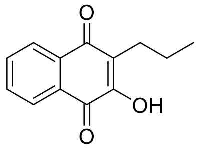

Red solid; Mp: 147.1-147.9 °C; 153 mg, yield: 71%;

^{1}H NMR (600 MHz, Chloroform-*d*) δ 8.05 (d, *J* = 7.7 Hz, 1H, ArH), 8.01 (d, *J* = 7.6 Hz, 1H, ArH), 7.68 (t, *J* = 7.3 Hz, 1H, ArH), 7.61 (t, *J* = 7.5 Hz, 1H, ArH), 7.25 (d, *J* = 21.7 Hz, 1H, OH), 2.54 – 2.48 (m, 2H, CH_2), 1.50 (p, *J* = 7.5 Hz, 2H, CH_2), 0.92 (t, *J* = 7.4 Hz, 3H, CH_3).

^{13}C NMR (151 MHz, Chloroform-*d*) δ 183.70, 180.49, 152.11, 133.81, 131.98, 131.81, 128.47, 125.78, 125.03, 123.56, 24.27, 20.58, 13.18.

HRMS (TOF ES+): m/z calcd for $C_{13}H_{12}O_3$ $[M+H]^+$, 217.0859; found, 217.0858.

Spectroscopic Data of **4ak**

2-(heptan-3-yl)-3-hydroxynaphthalene-1,4-dione

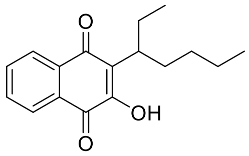

Red solid; Mp: 162.1-162.3 °C; 135 mg, yield: 50%;

^{1}H NMR (400 MHz, Chloroform-*d*) δ 8.02 (ddd, *J* = 15.3, 7.7, 1.3 Hz, 2H, ArH), 7.68 (td, *J* = 7.6, 1.4 Hz, 1H, ArH), 7.60 (td, *J* = 7.5, 1.4 Hz, 1H, ArH), 3.02 (tt, *J* = 9.5, 5.8 Hz, 1H, CH), 1.78 (ddq, *J* = 14.7, 11.7, 7.1 Hz, 2H, CH_2), 1.68 – 1.53 (m, 2H, CH_2), 1.20 (tdd, *J* = 13.4, 9.3, 6.1 Hz, 4H, CH_2), 0.77 (td, *J* = 7.3, 4.7 Hz, 6H, CH_3).

^{13}C NMR (101 MHz, Chloroform-*d*) δ 183.81, 180.53, 152.40, 133.85, 132.11, 131.71, 128.31, 125.96, 125.71, 124.94, 36.77, 31.42, 29.44, 24.89, 21.80, 13.03, 11.63.

HRMS (TOF ES+): m/z calcd for $C_{17}H_{20}O_3$ $[M+H]^+$, 273.1485; found, 273.1488.

Spectroscopic Data of **4al + 4al’**

2-hydroxy-6-methyl-2-phenylnaphthalene-1,4-dione

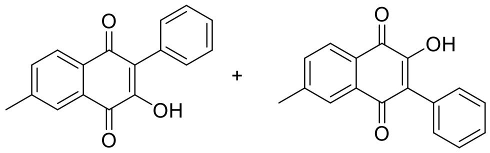

Red solid; Mp: 182.6-183.7 °C; 233 mg, yield: 88%;

^{1}H NMR (600 MHz, Chloroform-*d*) δ 7.99 (ddd, *J* = 26.1, 7.9, 2.2 Hz, 1H, ArH), 7.90 (d, *J* = 27.7 Hz, 1H, OH), 7.53 (s, 1H, ArH), 7.51 (d, *J* = 14.7 Hz, 1H, ArH), 7.43 (d, *J* = 7.9 Hz, 2H, ArH), 7.39 (t, *J* = 7.7 Hz, 2H, ArH), 7.32 (t, *J* = 7.3 Hz, 1H, ArH), 2.45 (d, *J* = 4.1 Hz, 3H, CH_3).

^{13}C NMR (151 MHz, Chloroform-*d*) δ 182.98, 182.69, 181.11, 180.53, 151.27, 151.08, 145.88, 143.16, 134.98, 134.95, 132.76, 131.83, 129.64, 129.62, 129.57, 129.06, 128.20, 127.58, 127.55, 126.90, 126.88, 126.78, 126.44, 125.99, 125.47, 125.39, 120.94, 120.81, 21.16, 20.63.

HRMS (TOF ES+): m/z calcd for $C_{17}H_{12}O_3$ $[M+H]^+$, 265.0859; found, 265.0860.

Spectroscopic Data of **4am+4am’**

2-hydroxy-6-methoxy-2-phenylnaphthalene-1,4-dione

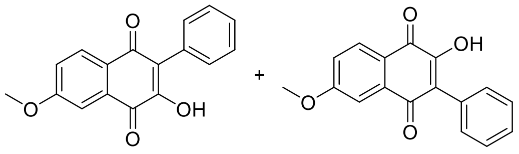

Red solid; Mp: 188.2-189.0 ^{o}C; 238 mg, yield: 85%;

^{1}**H NMR** (600 MHz, Chloroform-*d*) δ 8.02 (dd, *J* = 23.2, 7.6 Hz, 1H, ArH), 7.76 – 7.52 (m, 1H, OH), 7.51 – 7.44 (m, 1H, ArH), 7.43 (s, 2H, ArH), 7.37 (t, *J* = 7.7 Hz, 2H, ArH), 7.31 (t, *J* = 7.5 Hz, 1H, ArH), 7.18 – 7.06 (m, 1H, ArH), 3.87 (d, *J* = 6.0 Hz, 3H, CH3).

^{13}**C NMR** (151 MHz, Chloroform-*d*) δ 182.58, 182.12, 181.02, 179.34, 164.50, 162.45, 151.58, 150.97, 134.39, 129.98, 129.69, 129.56, 129.12, 129.06, 128.54, 127.83, 127.55, 127.47, 126.87, 126.84, 125.07, 121.49, 120.84, 120.48, 120.27, 118.30, 110.28, 108.66, 55.01, 54.94.

HRMS (TOF ES+): m/z calcd for $C_{17}H_{12}O_4$ [M+H]$^{+}$, 281.0808; found, 281.0813.

Spectroscopic Data of **5a**

ethyl 8-oxo-3,8a-diphenyl-8,8a-dihydroindeno[2,1-c]pyrazole-3a(3H)-carboxylate

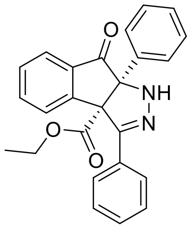

White solid; Mp: 212.4-213.7 ^{o}C; 328 mg, yield: 83%; > 95:5 dr;

IR (KBr): 3411, 2957, 2864, 1834, 1719, 1601, 1517, 1473, 1385, 1219, 772, 687 cm^{-1};

^{1}**H NMR** (600 MHz, Chloroform-*d*) δ 7.85 (d, *J* = 7.7 Hz, 1H, ArH), 7.75 (d, *J* = 7.9 Hz, 1H, ArH), 7.61 – 7.51 (m, 3H, ArH), 7.42 (t, *J* = 7.4 Hz, 1H, ArH), 7.29 – 7.19 (m, 8H, ArH), 6.58 (s, 1H, NH), 3.74 (dq, *J* = 10.6, 7.2 Hz, 1H, CH_2), 3.64 (dq, *J* = 10.6, 7.1 Hz, 1H, CH_2), 0.73 (t, *J* = 7.1 Hz, 3H, CH_3).

^{13}**C NMR** (151 MHz, Chloroform-*d*) δ 203.57, 167.55, 150.72, 148.39, 135.56, 134.67, 130.01, 128.47, 128.04, 127.95, 127.84, 127.70, 127.45, 127.33, 125.89, 125.65, 123.76, 83.85, 72.04, 60.99, 12.36;

HRMS (TOF ES+): m/z calcd for $C_{25}H_{20}N_2O_3$ [M+H]$^{+}$, 397.1547; found, 397.1556.

Spectroscopic Data of **5b**

ethyl8-oxo-3,8a-di-p-tolyl-8,8a-dihydroindeno[2,1-c]pyrazole-3a(3H)-carboxylate

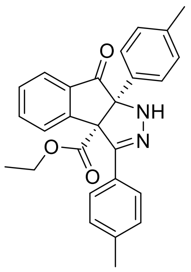

White solid; Mp: 217.1-217.8 ºC; 343 mg, yield: 81%; > 95:5 dr;

IR (KBr): 3410, 3020, 2931, 2805, 1830, 1717, 1608, 1524, 1476, 1385, 1220, 772, 690 cm^{-1};

^{1}H NMR (500 MHz, Chloroform-*d*) δ 7.82 (d, *J* = 7.6 Hz, 1H, ArH), 7.73 (d, *J* = 7.9 Hz, 1H, ArH), 7.56 (t, *J* = 7.6 Hz, 1H, ArH), 7.41 (dd, *J* = 14.9, 7.7 Hz, 3H, ArH), 7.14 – 6.97 (m, 6H, ArH), 6.51 (s, 1H, NH), 3.74 (dt, *J* = 14.2, 7.3 Hz, 1H, CH_2), 3.64 (dq, *J* = 13.7, 7.5 Hz, 1H, CH_2), 2.23 (d, *J* = 19.0 Hz, 6H, CH_3), 0.75 (t, *J* = 7.1 Hz, 3H, CH_3).

^{13}C NMR (126 MHz, Chloroform-*d*) δ 203.88, 167.66, 150.76, 148.51, 137.98, 137.46, 135.57, 134.56, 131.64, 128.13, 127.96, 127.92, 127.84, 127.22, 125.81, 125.57, 123.64, 83.76, 72.02, 60.88, 20.25, 20.08, 12.38;

HRMS (TOF ES+): m/z calcd for $C_{27}H_{24}N_2O_3$ $[M+H]^+$, 425.1860; found, 425.1863.

Spectroscopic Data of **5c**

ethyl 3,8a-bis(4-fluorop henyl)-8-oxo-8,8a-dihydroind eno[2,1-c]pyrazole -3a(3H)-ca rboxylate

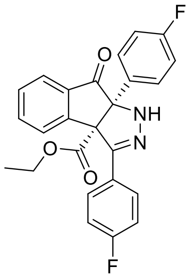

White solid; Mp: 234.1-235.2 ºC; 393 mg, yield: 91%; > 95:5 dr;

IR (KBr): 3403, 2943, 2855, 1817, 1712, 1578, 1538, 1465, 1374, 1209, 774, 685 cm^{-1};

^{1}H NMR (600 MHz, Chloroform-*d*) δ 7.86 (d, *J* = 7.7 Hz, 1H, ArH), 7.67 (d, *J* = 7.9 Hz, 1H, ArH), 7.60 (t, *J* = 7.6 Hz, 1H, ArH), 7.53 – 7.41 (m, 3H, ArH), 7.24 (dd, *J* = 8.5, 5.2 Hz, 2H, ArH), 6.94 (dt, *J* = 27.4, 8.5 Hz, 4H, ArH), 6.55 (d, *J* = 4.9 Hz, 1H, NH), 3.78 (dq, *J* = 10.7, 7.2 Hz, 1H, CH_2), 3.68 (dq, *J* = 10.5, 7.1 Hz, 1H, CH_2), 0.79 (t, *J* = 7.2 Hz, 3H, CH_3).

^{13}C NMR (151 MHz, Chloroform-*d*) δ 203.30, 167.32, 162.84 (d, *J* = 26.8 Hz), 161.18 (d, *J* = 25.4 Hz), 150.51, 147.62, 135.41, 134.86, 130.40, 128.25, 127.91, 127.85, 127.67, 127.60, 127.54, 126.11 (d, *J* = 3.2 Hz), 123.92, 114.69, 114.54, 114.40, 114.26, 83.37, 72.08, 61.16, 12.49;

^{19}F NMR (565 MHz, Chloroform-*d*) δ -111.28, -112.56;

HRMS (TOF ES+): m/z calcd for $C_{25}H_{18}F_2N_2O_3$ $[M+H]^+$, 433.1358; found, 433.1354.

Spectroscopic Data of **5d**

ethyl 3,8a-bis(4-chlorophenyl)-8-oxo-8,8a-dihydr oindeno[2,1-c]pyrazole -3a (3H)-c arboxylate

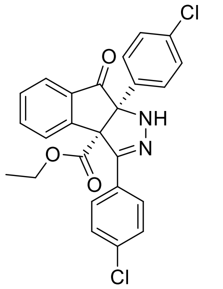

White solid; Mp: 195.3-196.1 ^{o}C; 408 mg, yield: 88%; > 95:5 dr;

IR (KBr): 3416, 2963, 2877, 1825, 1710, 1623, 1528, 1477, 1392, 1226, 771, 686 cm^{-1};

^{1}H NMR (600 MHz, Chloroform-*d*) δ 7.86 (d, *J* = 7.6 Hz, 1H, ArH), 7.69 (d, *J* = 7.9 Hz, 1H, ArH), 7.61 (td, *J* = 7.7, 1.3 Hz, 1H, ArH), 7.49 – 7.43 (m, 3H, ArH), 7.28 – 7.23 (m, 2H, ArH), 7.20 – 7.17 (m, 4H, ArH), 6.57 (s, 1H, NH), 3.79 (dq, *J* = 10.7, 7.2 Hz, 1H, CH_2), 3.68 (dq, *J* = 10.8, 7.2 Hz, 1H, CH_2), 0.79 (t, *J* = 7.1 Hz, 3H, CH_3).

^{13}C NMR (151 MHz, Chloroform-*d*) δ 202.84, 167.23, 150.41, 147.41, 135.35, 134.99, 134.04, 133.88, 133.14, 128.37, 128.33, 127.78, 127.67, 127.56, 127.39, 126.86, 124.01, 83.37, 73.93, 61.26, 12.47;

HRMS (TOF ES+): m/z calcd for $C_{25}H_{18}Cl_2N_2O_3$ $[M+H]^+$, 465.0767; found, 465.0764.

Spectroscopic Data of **5e**

ethyl 3,8a-bis(4-bromophenyl)-8-oxo-8,8a-dihydroindeno[2,1-c]pyrazole -3a(3H) -carboxylate

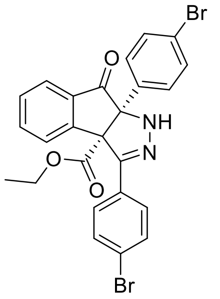

White solid; Mp: 207.3-208.5 °C; 464 mg, yield: 84%; > 95:5 dr;

IR (KBr): 3407, 2943, 2851, 1810, 1701, 1584, 1533, 1466, 1372, 1207, 686 cm^{-1};

^{1}H NMR (600 MHz, Chloroform-*d*) δ 7.86 (d, *J* = 7.7 Hz, 1H, ArH), 7.69 (d, *J* = 7.9 Hz, 1H, ArH), 7.61 (t, *J* = 7.6 Hz, 1H, ArH), 7.45 (t, *J* = 7.5 Hz, 1H, ArH), 7.40 (s, 2H, ArH), 7.37 – 7.34 (m, 2H, ArH), 7.19 (d, *J* = 1.6 Hz, 2H, ArH), 7.12 (d, *J* = 7.6 Hz, 2H, ArH), 6.56 (s, 1H, NH), 3.82 – 3.75 (m, 1H, CH_2), 3.72 – 3.65 (m, 1H, CH_2), 0.80 (t, *J* = 7.1 Hz, 3H, CH_3);

^{13}C NMR (151 MHz, Chloroform-*d*) δ 202.71, 167.21, 150.39, 147.45, 135.34, 135.01, 133.67, 130.73, 130.53, 128.81, 128.34, 127.67, 127.09, 124.03, 122.28, 122.04, 83.42, 71.85, 61.29, 12.46;

HRMS (TOF ES+): m/z calcd for $C_{25}H_{18}Br_2N_2O_3$ $[M+H]^+$, 554.9736; found, 554.9731.

Spectroscopic Data of **5f**

ethyl 3,8a-bis(4-methoxyphenyl)-8-oxo-8,8a-dihydroindeno[2,1-c]pyrazole -3a(3H)-carboxylate

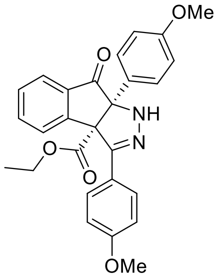

White solid; Mp: 231.7-232.3 °C; 360 mg, yield: 79%; > 95:5 dr;

IR (KBr): 3412, 3019, 2983, 2874, 2400, 1830, 1715, 1610, 1538, 1465, 1390, 1225, 824, 768, 626 cm^{-1};

^{1}H NMR (600 MHz, Chloroform-*d*) δ 7.84 (d, *J* = 7.6 Hz, 1H, ArH), 7.71 (d, *J* = 7.9 Hz, 1H, ArH), 7.58 (t, *J* = 7.6 Hz, 1H, ArH), 7.51 – 7.36 (m, 3H, ArH), 7.17 (d, *J* = 8.4 Hz, 2H, ArH), 6.77 (dd, *J* = 30.3, 8.4 Hz, 4H, ArH), 6.45 (s, 1H, NH), 3.83 – 3.63 (m, 8H, CH_2 and OCH_3), 0.80 (t, *J* = 7.1 Hz, 3H, CH_3).

^{13}C NMR (151 MHz, Chloroform-*d*) δ 204.14, 167.71, 159.19, 158.87, 150.78, 148.44, 135.54, 134.57, 127.95, 127.79, 127.26, 127.13, 126.63, 123.64, 122.60, 112.90, 112.73, 83.60, 72.11, 60.94, 54.28, 12.52;

HRMS (TOF ES+): m/z calcd for $C_{27}H_{24}N_2O_5$ $[M+H]^+$, 457.1758; found, 457.1758.

Spectroscopic Data of **5g**

ethyl 8-oxo-3,8a-di-m-tolyl-8,8a-dihydroindeno[2,1-c]pyrazole-3a(3H)-carboxylate

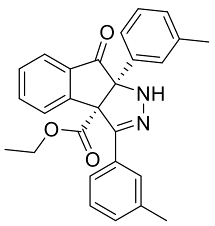

White solid; Mp: 213.3-214.5 ^{o}C; 339 mg, yield: 80%; > 95:5 dr;

IR (KBr): 3410, 3018, 2934, 2806, 1833, 1720, 1607, 1521, 1478, 1381, 1220, 680 cm^{-1};

^{1}H NMR (600 MHz, Chloroform-*d*) δ 7.85 (d, *J* = 7.7 Hz, 1H, ArH), 7.72 (d, *J* = 7.9 Hz, 1H, ArH), 7.58 (t, *J* = 7.6 Hz, 1H, ArH), 7.45 – 7.37 (m, 2H, ArH), 7.20 – 6.94 (m, 7H, ArH), 6.54 (s, NH), 3.75 (dq, *J* = 10.5, 7.2 Hz, 1H, CH_2), 3.69 – 3.61 (m, 1H, CH_2), 2.23 (d, *J* = 23.3 Hz, 6H, CH_3), 0.74 (t, *J* = 7.1 Hz, 3H, CH_3).

^{13}C NMR (151 MHz, Chloroform-*d*) δ 203.65, 167.59, 150.77, 148.44, 137.21, 136.99, 135.55, 134.63, 134.57, 129.92, 128.78, 128.42, 127.98, 127.82, 127.21, 126.57, 126.50, 123.73, 122.92, 122.59, 83.77, 72.06, 60.89, 20.41, 20.40, 12.33;

HRMS (TOF ES+): m/z calcd for $C_{27}H_{24}N_2O_3$ $[M+H]^+$, 425.1860; found, 425.1860.

Spectroscopic Data of **5h**

ethyl 3,8a-bis(3-chlorophenyl)-8-oxo-8,8a-dihydroind eno[2,1-c]pyrazole
-3a(3H)-carboxylate

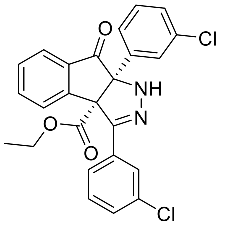

White solid; Mp: 185.6-186.2 ^{o}C; 394 mg, yield: 85%; > 95:5 dr;

IR (KBr): 3416, 2957, 2869, 1826, 1713, 1620, 1476, 1395, 1219, 872, 776, 684 cm^{-1};

^{1}H NMR (600 MHz, Chloroform-*d*) δ 7.88 (d, *J* = 7.7 Hz, 1H, ArH), 7.70 (d, *J* = 7.9 Hz, 1H, ArH), 7.63 (t, *J* = 7.6 Hz, 1H, ArH), 7.55 (d, *J* = 2.4 Hz, 1H, ArH), 7.50 –

7.41 (m, 2H, ArH), 7.40 – 7.35 (m, 1H, ArH), 7.22 (q, *J* = 4.0, 3.0 Hz, 3H, ArH), 7.14 (t, *J* = 7.9 Hz, 1H, ArH), 6.97 (d, *J* = 7.8 Hz, 1H, ArH), 6.62 (s, 1H, NH), 3.80 (dq, *J* = 10.8, 7.1 Hz, 1H, CH_2), 3.72 (dq, *J* = 10.7, 7.1 Hz, 1H, CH_2), 0.82 (t, *J* = 6.1 Hz, 3H, CH_3).

^{13}C NMR (151 MHz, Chloroform-*d*) δ 202.51, 167.03, 150.32, 146.92, 136.61, 135.28, 135.12, 133.66, 133.56, 131.61, 128.68, 128.64, 128.40, 128.08, 128.06, 127.67, 126.63, 125.83, 124.08, 123.89, 123.47, 83.28, 71.93, 61.35, 12.41;

HRMS (TOF ES+): m/z calcd for $C_{25}H_{18}Cl_2N_2O_3$ $[M+H]^+$, 465.0767; found, 465.0763.

Spectroscopic Data of **5i**

ethyl 3,8a-bis(3-fluorophenyl)-8-oxo-8,8a-dihydroindeno[2,1-c]pyrazole
-3a(1H)-carboxylate

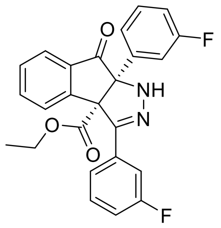

White solid; Mp: 201.3-202.8 ^{o}C; 388 mg, yield: 90%; > 95:5 dr;

IR (KBr): 3402, 2942, 2857, 1814, 1711, 1570, 1534, 1459, 1370, 1210, 683 cm^{-1};

^{1}H NMR (600 MHz, Acetone-d_6) δ 8.16 (s, 1H, ArH), 7.79 (dd, *J* = 16.0, 7.8 Hz, 2H, ArH), 7.70 – 7.64 (m, 1H, ArH), 7.50 (t, *J* = 7.5 Hz, 1H, ArH), 7.39 – 7.22 (m, 4H, ArH), 7.00 (dddd, *J* = 18.5, 16.0, 9.3, 2.7 Hz, 3H, ArH), 6.88 (d, *J* = 7.9 Hz, 1H, NH), 3.69 (dq, *J* = 10.8, 7.1 Hz, 1H, CH_2), 3.60 (dq, *J* = 10.8, 7.1 Hz, 1H, CH_2), 0.68 (t, *J* = 7.1 Hz, 3H, CH_3).

^{13}C NMR (151 MHz, Acetone-d_6) δ 201.74, 167.95,163.44 (d, *J* = 24 Hz), 161.83 (d, *J* = 25.5 Hz), 151.10, 145.50, 139.03 (d, *J* = 7.5 Hz), 135.97, 133.82 (d, J = 7.5 Hz), 130.51(d, *J* = 7.5 Hz), 130.15(d, *J* = 7.5Hz), 129.46, 128.63, 124.62, 123.07(d, *J* = 1.5Hz), 122.26 (d, *J* = 1.5Hz), 115.47, 115.33, 115.18, 114.49 (d, *J* = 24 Hz), 112.78 (d, *J* = 24 Hz), 84.69, 73.15, 61.79, 12.81;

^{19}F NMR (565 MHz, Acetone-*d6*) δ -112.59, -113.30;

HRMS (TOF ES+): m/z calcd for $C_{25}H_{18}F_2N_2O_3$ $[M+H]^+$, 433.1358; found, 433.1356.

Spectroscopic Data of **5j**

ethyl 3,8a-bis(3-bromophenyl)-8-oxo-8, 8a-dihydroi ndeno[2,1-c]pyrazole -3a(3H) -carboxylate

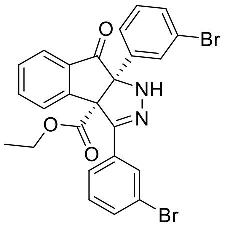

White solid; Mp: 175.8-176.1 °C; 447 mg, yield: 81%; > 95:5 dr;

IR (KBr): 3404, 2955, 2860, 1852, 1731, 1631, 1528, 1470, 1381, 1251, 865, 767, 682 cm^{-1};

^{1}H NMR (600 MHz, Chloroform-*d*) δ 7.87 (d, *J* = 7.6 Hz, 1H, ArH), 7.73 – 7.66 (m, 2H, ArH), 7.64 – 7.59 (m, 2H, ArH), 7.48 – 7.35 (m, 4H, ArH), 7.15 (t, *J* = 7.9 Hz, 1H, ArH), 7.06 (t, *J* = 7.9 Hz, 1H, ArH), 6.99 (d, *J* = 7.9 Hz, 1H, ArH), 6.64 (s, 1H, NH), 3.80 (dq, *J* = 10.8, 7.1 Hz, 1H, CH_2), 3.71 (dq, *J* = 10.8, 7.1 Hz, 1H, CH_2), 0.83 (t, *J* = 7.1 Hz, 3H, CH_3).

^{13}C NMR (151 MHz, Chloroform-*d*) δ 202.41, 167.00, 150.31, 146.71, 136.89, 135.31, 135.12, 131.89, 131.01, 130.97, 129.57, 128.91, 128.88, 128.80, 128.41, 127.65, 124.37, 124.09, 123.92, 121.76, 121.67, 83.25, 72.02, 61.37, 12.46;

HRMS (TOF ES+): m/z calcd for $C_{25}H_{18}Br_2N_2O_3$ $[M+H]^+$, 554.9736; found, 554.9734.

Spectroscopic Data of **5k**

ethyl 3,8a-bis(3-methoxyphenyl)-8-oxo-8,8a-dihydroindeno[2,1-c]pyrazole-3a(3H) -carboxylate

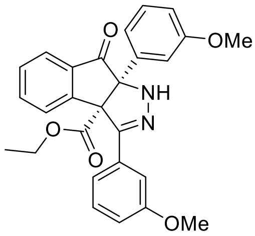

White solid; Mp: 218.3-219.1 °C; 351 mg, yield: 77%; > 95:5 dr;

IR (KBr):3418, 3023, 2887, 1834, 1717, 1605, 1508, 1470, 1385, 1220, 827, 766, 631 cm^{-1};

^{1}H NMR (600 MHz, Chloroform-*d*) δ 7.84 (d, *J* = 7.7 Hz, 1H, ArH), 7.78 (d, *J* = 7.9 Hz, 1H, ArH), 7.59 (t, *J* = 7.6 Hz, 1H, ArH), 7.42 (t, *J* = 7.5 Hz, 1H, ArH), 7.19 – 7.17 (m, 1H, ArH), 7.13 – 7.07 (m, 3H, ArH), 6.95 (s, 1H, ArH), 6.80 (dd, *J* = 8.3, 2.6 Hz, 1H, ArH), 6.75 (dd, *J* = 8.2, 2.6 Hz, 1H, ArH), 6.67 (d, *J* = 7.8 Hz, 1H, ArH), 6.57 (s, 1H, NH), 3.81 – 3.72 (m, 2H, CH_2), 3.71 (s, 3H, CH_3), 3.68 (s, 3H, CH_3), 0.75 (t, *J* = 7.2 Hz, 3H, CH_3);

^{13}C NMR (151 MHz, Chloroform-*d*) δ 203.33, 167.45, 158.66, 158.63, 150.74, 148.40, 136.16, 135.51, 134.67, 131.28, 128.41, 128.34, 128.04, 128.01, 123.75, 117.94, 117.91, 114.32, 113.39, 111.82, 110.80, 83.73, 71.98, 60.99, 54.26, 54.23, 12.40;

HRMS (TOF ES+): m/z calcd for $C_{27}H_{24}N_2O_5$ $[M+H]^+$, 457.1758; found, 457.1756.

Spectroscopic Data of **5l**

ethyl 3,8a-bis(2-chlorophenyl)-8-oxo-8,8a-dihydroindeno[2,1-c]pyrazole
-3a(3H)-carboxylate

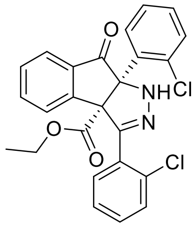

White solid; Mp: 195.2-195.8 °C; 338 mg, yield: 73%; > 95:5 dr;

IR (KBr): 3411, 2966, 1833, 1721, 1647, 1543, 1485, 1407, 1233, 836, 693 cm^{-1};

^{1}H NMR (600 MHz, Chloroform-*d*) δ 8.22 (d, *J* = 7.6 Hz, 1H, ArH), 7.89 (d, *J* = 7.6 Hz, 1H, ArH), 7.52 – 7.40 (m, 2H, ArH), 7.35 (t, *J* = 7.5 Hz, 1H, ArH), 7.27 – 7.16 (m, 6H, ArH), 7.05 (d, *J* = 7.7 Hz, 1H, ArH), 6.41 (s, 1H, NH), 3.86 – 3.73 (m, 2H, CH_2), 0.77 (t, *J* = 7.1 Hz, 3H, CH_3).

^{13}C NMR (151 MHz, Chloroform-*d*) δ 200.61, 166.09, 149.05, 143.80, 135.88, 134.81, 134.55, 134.18, 131.34, 130.84, 129.43, 129.38, 129.23, 128.85, 128.70, 127.91, 126.04, 125.82, 125.47, 123.83, 81.15, 73.97, 60.95, 12.29;

HRMS (TOF ES+): m/z calcd for $C_{25}H_{18}Cl_2N_2O_3$ $[M+H]^+$, 465.0767; found, 465.0760.

Spectroscopic Data of **5m**

ethyl 3,8a-bis(2-fluorophenyl)-8-oxo-8,8a-dihydroindeno[2,1-c]pyrazole
-3a(3H)-carboxylate

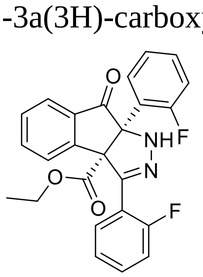

White solid; Mp: 227.8-228.5 °C; 349 mg, yield: 81%; > 95:5 dr;

IR (KBr): 3413, 3023, 2930, 2801, 1835, 1718, 1605, 1520, 1479, 1378, 1216, 785,

697 cm^{-1};

^{1}H NMR (600 MHz, Chloroform-*d*) δ 7.98 – 7.91 (m, 1H, ArH), 7.84 (d, *J* = 7.7 Hz, 1H, ArH), 7.60 (dd, *J* = 9.4, 7.2 Hz, 2H, ArH), 7.56 – 7.51 (m, 1H, ArH), 7.39 (t, *J* = 7.4 Hz, 1H, ArH), 7.23 (tdd, *J* = 7.4, 5.1, 2.0 Hz, 2H, ArH), 7.14 (td, *J* = 7.7, 1.2 Hz, 1H, ArH), 7.04 – 6.98 (m, 2H, ArH), 6.87 – 6.81 (m, 1H, ArH), 6.50 (dd, *J* = 6.1, 3.2 Hz, 1H, NH), 3.76 (dq, *J* = 10.8, 7.1 Hz, 1H, CH_2), 3.60 (dq, *J* = 10.8, 7.2 Hz, 1H, CH_2), 0.81 (t, *J* = 7.1 Hz, 3H, CH_3);

^{13}C NMR (151 MHz, Chloroform-*d*) δ 202.46, 166.51, 159.50 (d, J = 114.7 Hz), 157.85 (d, *J* = 114.0 Hz), 149.77, 144.48, 135.17 (d, *J* = 4.2 Hz), 134.83, 130.05 (d, *J* = 3.7 Hz), 129.77 (d, *J* = 8.8 Hz), 129.69 (d, *J* = 8.3 Hz), 129.37 (d, *J* = 3.7 Hz), 128.00, 126.01 (d, *J* = 7.6 Hz), 123.80, 123.49 (d, *J* = 3.3 Hz), 122.96 (d, *J* = 3.2 Hz), 122.62 (d, *J* = 13.8 Hz), 117.68 (d, *J* = 12.3 Hz), 115.23 (d, *J* = 23.2 Hz), 114.70 (d, *J* = 21.7 Hz), 80.96, 72.43, 60.69, 12.49;

^{19}F NMR (565 MHz, Chloroform-*d*) δ -109.50, -109.51;

HRMS (TOF ES+): m/z calcd for $C_{25}H_{18}F_2N_2O_3$ $[M+H]^+$, 433.1358; found, 433.1354.

Spectroscopic Data of **5n**

ethyl 3,8a-bis(2-methoxyphenyl)-8-oxo-8,8a-dihydroindeno[2,1-c]pyrazole
-3a(3H)-carboxylate

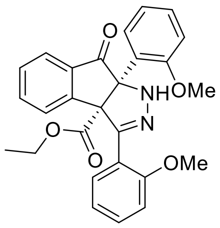

White solid; Mp: 231.6-232.3 °C; 319 mg, yield: 70%; > 95:5 dr;

IR (KBr): 3433, 3027, 2980, 2870, 2407, 1834, 1718, 1613, 1532, 1457, 1382, 1227, 778, 621 cm^{-1};

^{1}H NMR (600 MHz, Chloroform-*d*) δ 7.97 (d, *J* = 7.8 Hz, 1H, ArH), 7.75 (d, *J* = 7.6 Hz, 1H, ArH), 7.46 (d, *J* = 7.7 Hz, 1H, ArH), 7.43 – 7.36 (m, 2H, ArH), 7.29 (t, *J* = 7.3 Hz, 1H, ArH), 7.18 (q, *J* = 8.1 Hz, 2H, ArH), 6.94 (t, *J* = 7.6 Hz, 1H, ArH), 6.80 – 6.74 (m, 2H, ArH), 6.61 (d, *J* = 8.1 Hz, 1H, ArH), 6.32 (s, 1H, NH), 3.65 (s, 4H, CH_2 and OCH_3), 3.46 (dq, *J* = 10.6, 7.1 Hz, 1H, CH_2), 3.09 (s, 3H, OCH_3), 0.74 (t, *J* = 7.2 Hz, 3H, CH_3).

^{13}C NMR (151 MHz, Chloroform-*d*) δ 204.66, 167.00, 155.83, 154.92, 150.05, 147.11, 136.37, 133.49, 129.70, 129.55, 129.24, 128.71, 126.99, 125.64, 125.02, 122.41, 119.86, 119.66, 119.19, 110.37, 109.81, 81.74, 72.42, 59.82, 53.90, 52.97,

12.50;

HRMS (TOF ES+): m/z calcd for $C_{27}H_{24}N_2O_5$ $[M+H]^+$, 457.1758; found, 457.1756.

Spectroscopic Data of **5o**

methyl 8-oxo-3,8a-diphenyl-8,8a-dihydroindeno[2,1-c]pyrazole-3a(3H)-carboxylate

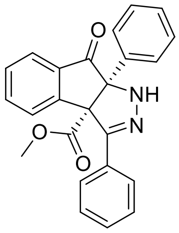

White solid; Mp: 205.3-206.1 °C; 332 mg, yield: 87%; > 95:5 dr;

IR (KBr): 3410, 2951, 1834, 1720, 1603, 1511, 1381, 1131, 763, 677 cm^{-1};

^{1}H NMR (600 MHz, DMSO-d_6) δ 9.12 (s, 1H, NH), 7.95 (d, *J* = 7.7 Hz, 1H, ArH), 7.80 (d, *J* = 7.1 Hz, 2H, ArH), 7.62 (dd, *J* = 10.3, 7.3 Hz, 3H, ArH), 7.42 – 7.36 (m, 5H, ArH), 7.32 (t, *J* = 7.3 Hz, 1H, ArH), 7.19 – 7.15 (m, 2H, ArH), 3.12 (s, 3H, CH_3);

^{13}C NMR (151 MHz, DMSO-*d6*) δ 202.14, 169.00, 150.86, 144.48, 136.69, 136.25, 136.24, 131.39, 129.94, 129.17, 129.02, 128.65, 128.49, 127.61, 126.33, 125.00, 85.37, 73.70, 52.70;

HRMS (TOF ES+): m/z calcd for $C_{24}H_{18}N_2O_3$ $[M+H]^+$, 383.1390; found, 383.1385.

Spectroscopic Data of **5p**

propyl 8-oxo-3,8a-diphenyl-8,8a-dihydroindeno[2,1-c]pyrazole-3a(3H)-carboxylate

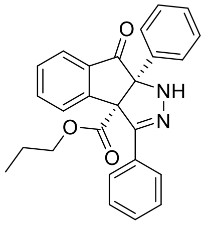

White solid; Mp: 217.5-218.1 °C; 266 mg, yield: 65%; > 95:5 dr;

IR (KBr): 3419, 3012, 2925, 2843, 1831, 1719, 1602, 1507, 1482, 1381, 1230, 751, 681 cm^{-1};

^{1}H NMR (600 MHz, Chloroform-*d*) δ 7.85 (d, *J* = 7.7 Hz, 1H, ArH), 7.74 (d, *J* = 7.9 Hz, 1H, ArH), 7.61 – 7.52 (m, 3H, ArH), 7.42 (t, *J* = 7.4 Hz, 1H, ArH), 7.29 – 7.20 (m, 8H, ArH), 6.58 (s, 1H, NH), 3.67 (dt, *J* = 10.6, 6.8 Hz, 1H, CH_2), 3.40 (dt, *J* = 10.6, 6.7 Hz, 1H, CH_2), 1.18 – 1.11 (m, 2H, CH_2), 0.57 (t, *J* = 7.4 Hz, 3H, CH_3).

^{13}C NMR (151 MHz, Chloroform-*d*) δ 203.60, 167.62, 150.70, 148.36, 135.55,

134.69, 134.61, 129.99, 128.04, 127.94, 127.81, 127.69, 127.43, 127.32, 125.87, 125.64, 123.75, 83.91, 72.15, 66.55, 20.27, 9.19;

HRMS (TOF ES+): m/z calcd for $C_{26}H_{22}N_2O_3$ $[M+H]^+$, 411.1703; found, 411.1701.

Spectroscopic Data of **5q**

ethyl 8-oxo-3,8a-di(thiophen-3-yl)-8,8a-dihydroindeno[2,1-c]pyrazole-3a(3H)-carboxylate

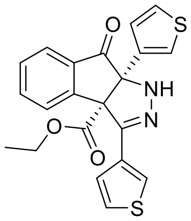

White solid; Mp: 187.3-188.1 ^{o}C; 346 mg, yield: 85%; > 95:5 dr;

IR (KBr): 3416, 2951, 2860, 1828, 1758, 1583, 1511, 1473, 1378, 1215, 815, 764, 617 cm^{-1};

^{1}H NMR (500 MHz, Chloroform-*d*) δ 7.86 (d, *J* = 7.7 Hz, 1H, ArH), 7.75 (d, *J* = 7.9 Hz, 1H, ArH), 7.63 (t, *J* = 7.6 Hz, 1H, ArH), 7.49 – 7.37 (m, 2H, ArH), 7.31 (dd, *J* = 13.4, 4.1 Hz, 2H, ArH), 7.24 – 7.16 (m, 2H, ArH), 6.74 (d, *J* = 5.1 Hz, 1H, ArH), 6.57 (s, 1H, NH), 3.81 (pd, *J* = 10.8, 5.4 Hz, 2H, CH_2), 0.83 (t, *J* = 7.1 Hz, 3H, CH_3).

^{13}C NMR (126 MHz, Chloroform-*d*) δ 202.10, 167.28, 149.87, 145.38, 134.93, 134.85, 134.62, 131.59, 128.23, 127.55, 125.78, 125.14, 124.99, 124.79, 123.99, 123.29, 122.06, 81.40, 71.99, 61.15, 12.51;

HRMS (TOF ES+): m/z calcd for $C_{21}H_{16}N_2O_3S_2$ $[M+H]^+$, 409.0675; found, 409.0678.

Spectroscopic Data of **5r**

methyl 8-oxo-3,8a-di(thiophen-3-yl)-8,8a-dihydroindeno[2,1-c]pyrazole -3a(3H)-carboxylate

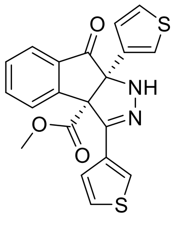

White solid; Mp: 191.4-192.7 ^{o}C; 346 mg, yield: 88%; > 95:5 dr;

IR (KBr): 3410, 2955, 1830, 1724, 1604, 1524, 1380, 1137, 814, 767, 679 cm^{-1};

^{1}H NMR (600 MHz, Chloroform-*d*) δ 7.86 (d, *J* = 7.7 Hz, 1H, ArH), 7.72 (d, *J* = 7.9 Hz, 1H, ArH), 7.62 (t, *J* = 7.6 Hz, 1H, ArH), 7.45 (t, *J* = 7.5 Hz, 1H, ArH), 7.38 (d, *J* = 2.8 Hz, 1H, ArH), 7.30 (s, 2H, ArH), 7.20 (ddd, *J* = 19.6, 4.9, 3.0 Hz, 2H, ArH), 6.75 (d, *J* = 5.1 Hz, 1H, ArH), 6.60 (s, 1H, NH), 3.31 (s, 3H, CH_3).

^{13}C NMR (151 MHz, Chloroform-*d*) δ 201.95, 167.75, 149.59, 145.06, 145.05, 134.91, 134.71, 131.61, 128.31, 127.45, 125.76, 125.09, 125.07, 124.82, 124.00, 123.35, 122.11, 81.52, 72.24, 51.66;

HRMS (TOF ES+): m/z calcd for $C_{20}H_{14}N_2O_3S_2$ $[M+H]^+$, 395.0519; found, 395.0518.

Spectroscopic Data of **5s**

propyl 8-oxo-3,8a-di(thiophen-3-yl)-8,8a-dihydroindeno[2,1-c]pyrazole
-3a(3H)-carboxylate

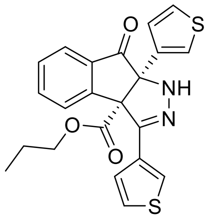

White solid; Mp: 197.1-198.3 ^oC; 295 mg, yield: 70%; > 95:5 dr;

IR (KBr): 3414, 3008, 2921, 2839, 1830, 1712, 1607, 1511, 1485, 1383, 1233, 828, 754, 652 cm^{-1};

^{1}H NMR (600 MHz, Chloroform-*d*) δ 7.85 (dd, *J* = 7.7, 1.2 Hz, 1H, ArH), 7.74 (d, *J* = 7.9 Hz, 1H, ArH), 7.63 (td, *J* = 7.6, 1.3 Hz, 1H, ArH), 7.45 (dd, *J* = 6.4, 2.1 Hz, 1H, ArH), 7.39 (dd, *J* = 2.9, 1.3 Hz, 1H, ArH), 7.31 (dd, *J* = 3.3, 1.7 Hz, 1H, ArH), 7.21 (dd, *J* = 5.1, 2.9 Hz, 1H, ArH), 7.19 – 7.16 (m, 2H, ArH), 6.73 (dd, *J* = 5.1, 1.3 Hz, 1H, ArH), 6.57 (s, 1H, NH), 3.74 (dt, *J* = 10.7, 6.8 Hz, 1H, CH_2), 3.57 (dt, *J* = 10.7, 6.7 Hz, 1H, CH_2), 1.27 – 1.24 (m, 2H, CH_2), 0.61 (t, *J* = 7.4 Hz, 3H, CH_3);

^{13}C NMR (151 MHz, Chloroform-*d*) δ 203.08, 167.33, 149.89, 145.35, 134.81, 134.68, 131.67, 128.52, 128.23, 127.52, 125.80, 125.10, 124.95, 124.81, 123.98, 123.28, 122.07, 81.48, 72.17, 66.68, 20.42, 9.15;

HRMS (TOF ES+): m/z calcd for $C_{22}H_{18}N_2O_3S_2$ $[M+Na]^+$, 445.0651; found, 445.0655;

Spectroscopic Data of **5t**

ethyl 3,8a-di(naphthalen-2-yl)-8-oxo-8,8a-dihydroindeno[2,1-c]pyrazole
-3a(3H)-carboxylate

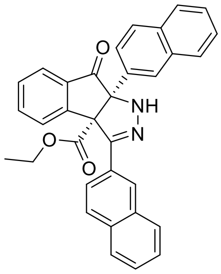

White solid; Mp: 241.3-242.7 °C; 401 mg, yield: 81%; > 95:5 dr;

IR (KBr): 3435, 2864, 2869, 1840, 1724, 1604, 1507, 1470, 1382, 1215, 782, 619 cm^{-1};

^{1}H NMR (600 MHz, Chloroform-*d*) δ 7.94 (d, *J* = 2.1 Hz, 2H, ArH), 7.90 (d, *J* = 7.7 Hz, 1H, ArH), 7.85 (d, *J* = 7.9 Hz, 1H, ArH), 7.78 – 7.70 (m, 6H, ArH), 7.66 (d, *J* = 8.6 Hz, 1H, ArH), 7.56 (t, *J* = 7.7 Hz, 1H, ArH), 7.42 – 7.36 (m, 5H, ArH), 7.19 (d, *J* = 8.6 Hz, 1H, ArH), 6.79 (s, 1H, NH), 3.65 (dq, *J* = 10.9, 7.1 Hz, 1H, CH_2), 3.50 (dq, *J* = 10.8, 7.1 Hz, 1H, CH_2), 0.55 (t, *J* = 7.1 Hz, 3H, CH_3).

^{13}C NMR (151 MHz, Chloroform-*d*) δ 203.30, 167.71, 150.80, 148.26, 135.54, 134.86, 132.39, 132.29, 132.01, 131.92, 131.86, 128.14, 127.87, 127.53, 127.34, 127.26, 127.22, 127.10, 126.71, 126.55, 125.91, 125.74, 125.55, 125.51, 125.38, 124.60, 123.91, 123.50, 123.03, 84.07, 72.18, 61.02, 12.26;

HRMS (TOF ES+): m/z calcd for $C_{33}H_{24}N_2O_5$ $[M+H]^+$, 497.1860; found, 497.1856.

Spectroscopic Data of **5u**

methyl 3,8a-di(naphthalen-2-yl)-8-oxo-8,8a-dihydroindeno[2,1-c]pyrazole
-3a(3H)-carboxylate

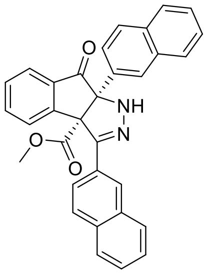

White solid; Mp: 243.7-244.9 °C; 405 mg, yield: 84%; > 95:5 dr;

IR (KBr): 3425, 2984, 1844, 1740, 1610, 1513, 1392, 1133, 780, 623 cm^{-1};

^{1}H NMR (600 MHz, Chloroform-*d*) δ 7.90 (t, *J* = 7.1 Hz, 3H, ArH), 7.84 (d, *J* = 8.0 Hz, 1H, ArH), 7.77 (td, *J* = 5.9, 3.0 Hz, 2H, ArH), 7.72 – 7.64 (m, 5H, ArH), 7.55 (t, *J* = 7.6 Hz, 1H, ArH), 7.43 – 7.34 (m, 5H, ArH), 7.20 (d, *J* = 8.6 Hz, 1H, ArH), 6.84 (s, 1H, NH), 3.07 (s, 3H, CH_3).

^{13}C NMR (151 MHz, Chloroform-*d*) δ 203.23, 168.17, 150.51, 148.03, 135.58,

134.92, 132.38, 132.26, 131.95, 131.87, 131.85, 128.20, 127.79, 127.46, 127.39, 127.29, 127.27, 127.07, 126.69, 126.55, 125.83, 125.77, 125.56, 125.37, 124.55, 123.89, 123.41, 122.99, 84.23, 72.34, 51.53;

HRMS (TOF ES+): m/z calcd for $C_{32}H_{22}N_2O_3$ $[M+H]^+$, 483.1703; found, 483.1698.

Spectroscopic Data of **5v**

propyl 3,8a-di(naphthalen-2-yl)-8-oxo-8,8a-dihydroindeno[2,1-c]pyrazole -3a(3H)-carboxylate

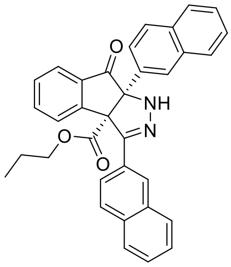

White solid; Mp: 235.3-236.1 °C; 336 mg, yield: 66%; > 95:5 dr;

IR (KBr): 3442, 3037, 2934, 2852, 1838, 1725, 1611, 1534, 1482, 1388, 1237, 841, 774, 653 cm^{-1};

^{1}H NMR (600 MHz, Chloroform-*d*) δ 7.94 (s, 2H, ArH), 7.89 (d, *J* = 7.7 Hz, 1H, ArH), 7.84 (d, *J* = 8.0 Hz, 1H, ArH), 7.77 (ddd, *J* = 12.8, 6.4, 3.9 Hz, 2H, ArH), 7.71 (td, *J* = 7.7, 6.1, 2.7 Hz, 4H, ArH), 7.66 (d, *J* = 8.7 Hz, 1H, ArH), 7.56 (td, *J* = 7.7, 1.3 Hz, 1H, ArH), 7.43 – 7.37 (m, 5H, ArH), 7.19 (d, *J* = 8.6 Hz, 1H, ArH), 6.79 (s, 1H, NH), 3.61 (dt, *J* = 10.8, 6.7 Hz, 1H, CH_2), 3.25 (dt, *J* = 10.7, 6.6 Hz, 1H, CH_2), 1.02 (q, *J* = 7.1 Hz, 2H, CH_2), 0.40 (t, *J* = 7.4 Hz, 3H, CH_3).

^{13}C NMR (151 MHz, Chloroform-*d*) δ 203.31, 167.76, 150.82, 148.28, 135.57, 134.83, 132.39, 132.31, 131.99, 131.93, 131.87, 128.13, 127.85, 127.57, 127.32, 127.24, 127.09, 126.71, 126.55, 125.92, 125.72, 125.55, 125.51, 125.35, 124.64, 123.88, 123.52, 123.04, 84.15, 72.32, 66.58, 20.22, 9.05;

HRMS (TOF ES+): m/z calcd for $C_{34}H_{26}N_2O_3$ $[M+H]^+$, 511.2016; found, 511.2010.

Spectroscopic Data of **5w**

ethyl 3,8a-bis(3,4-dimethylphenyl)-8-oxo-8,8a-dihydroindeno[2,1-c]pyrazole -3a(3H)-carboxylate

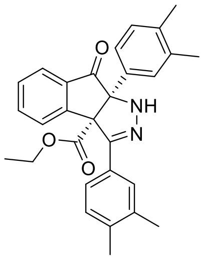

White solid; Mp: 233.8-234.7 °C; 361 mg, yield: 80%; > 95:5 dr;

IR (KBr): 3427, 3125, 3012, 2871, 1851, 1722, 1619, 1523, 1472, 1384, 1225, 833, 774, 626 cm^{-1};

^{1}H NMR (600 MHz, Chloroform-*d*) δ 7.83 (d, *J* = 7.6 Hz, 1H, ArH), 7.73 (d, *J* = 7.9 Hz, 1H, ArH), 7.56 (t, *J* = 7.6 Hz, 1H, ArH), 7.42 – 7.34 (m, 2H, ArH), 7.22 (d, *J* = 7.9 Hz, 1H, ArH), 7.06 – 7.00 (m, 2H, ArH), 6.94 (d, *J* = 7.9 Hz, 1H, ArH), 6.89 (d, *J* = 7.9 Hz, 1H, ArH), 6.48 (s, 1H, NH), 3.75 (dq, *J* = 10.7, 7.1 Hz, 1H, CH_2), 3.66 (dq, *J* = 10.8, 7.1 Hz, 1H, CH_2), 2.16 (d, *J* = 4.4 Hz, 6H, CH_3), 2.12 (d, *J* = 5.7 Hz, 6H, CH_3), 0.74 (t, *J* = 7.1 Hz, 3H, CH_3).

^{13}C NMR (151 MHz, Chloroform-*d*) δ 203.98, 167.70, 150.79, 148.53, 136.74, 136.06, 135.81, 135.54, 135.46, 134.56, 131.91, 128.50, 128.49, 127.88, 127.82, 127.53, 127.06, 126.97, 123.62, 123.25, 122.89, 83.67, 71.97, 60.80, 18.77, 18.74, 18.59, 18.41, 12.32;

HRMS (TOF ES+): m/z calcd for $C_{29}H_{28}N_2O_3$ $[M+H]^+$, 453.2173; found, 453.2170.

Spectroscopic Data of **5x**

methyl3,8a-bis(3,4-dimethylphenyl)-8-oxo-8,8a-dihydroindeo[2,1-c]pyrazole -3a(3H)-carboxylate

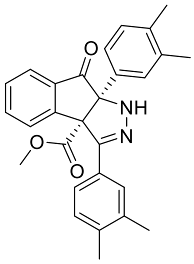

White solid; Mp: 237.3-238.5 °C; 372 mg, yield: 85%; > 95:5 dr;

IR (KBr): 3433, 3122, 3013, 2865, 1847, 1720, 1620, 1522, 1390, 1137, 831, 761, 615 cm^{-1};

^{1}H NMR (600 MHz, Chloroform-*d*) δ 7.84 (d, *J* = 7.6 Hz, 1H, ArH), 7.72 (d, *J* = 7.9 Hz, 1H, ArH), 7.57 (t, *J* = 7.6 Hz, 1H, ArH), 7.40 (t, *J* = 7.5 Hz, 1H, ArH), 7.36 (d, *J* = 1.9 Hz, 1H, ArH), 7.21 (dd, *J* = 7.8, 1.9 Hz, 1H, ArH), 7.02 (d, *J* = 8.5 Hz, 2H,

ArH), 6.95 (d, *J* = 7.9 Hz, 1H, ArH), 6.89 (dd, *J* = 7.9, 2.1 Hz, 1H, ArH), 6.51 (s, 1H, NH), 3.22 (s, 3H, CH_3), 2.16 (d, *J* = 2.7 Hz, 6H, CH_3), 2.12 (d, *J* = 7.8 Hz, 6H, CH_3).

^{13}C NMR (151 MHz, Chloroform-*d*) δ 203.91, 168.18, 150.51, 148.32, 136.81, 136.13, 135.89, 135.62, 135.52, 134.63, 131.85, 128.55, 128.48, 127.95, 127.77, 127.49, 126.99, 126.93, 123.61, 123.27, 122.84, 83.88, 72.15, 51.32, 18.78, 18.73, 18.58, 18.43;

HRMS (TOF ES+): m/z calcd for $C_{28}H_{26}N_2O_3$ $[M+H]^+$, 439.2016; found, 439.2016.

Spectroscopic Data of **5y**

propyl 3,8a-bis(3,4-dimethylphenyl)-8-oxo-8,8a-dihydroindeno[2,1-c]pyrazole-3a(3H)-carboxylate

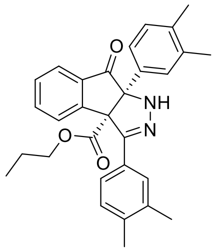

White solid; Mp: 253.1-254.2 °C; 279 mg, yield: 60%; > 95:5 dr;

IR (KBr): 3437, 3207, 3046, 2962, 2867, 1851, 1733, 1624, 1547, 1485, 1384, 1244, 863, 781, 663 cm^{-1};

^{1}H NMR (600 MHz, Chloroform-*d*) δ 7.83 (d, *J* = 7.6 Hz, 1H, ArH), 7.72 (d, *J* = 7.9 Hz, 1H, ArH), 7.56 (t, *J* = 7.6 Hz, 1H, ArH), 7.43 – 7.34 (m, 2H, ArH), 7.22 (d, *J* = 7.9 Hz, 1H, ArH), 7.06 – 7.00 (m, 2H, ArH), 6.94 (d, *J* = 7.9 Hz, 1H, ArH), 6.88 (d, *J* = 7.8 Hz, 1H, ArH), 6.48 (s, 1H, NH), 3.69 (dt, *J* = 10.6, 6.7 Hz, 1H, CH_2), 3.43 (dt, *J* = 10.6, 6.6 Hz, 1H, CH_2), 2.16 (d, *J* = 5.1 Hz, 6H, CH_3), 2.12 (d, *J* = 7.7 Hz, 6H, CH_3), 1.20 (d, *J* = 7.2 Hz, 2H, CH_2), 0.59 (t, *J* = 7.4 Hz, 3H, CH_3).

^{13}C NMR (151 MHz, Chloroform-*d*) δ 204.01, 167.76, 150.81, 148.56, 136.70, 136.05, 135.77, 135.56, 135.46, 134.55, 131.90, 128.49, 127.87, 127.80, 127.55, 127.07, 127.00, 123.60, 123.26, 122.94, 83.75, 72.11, 66.41, 20.33, 18.76, 18.75, 18.59, 18.41, 9.24;

HRMS (TOF ES+): m/z calcd for $C_{30}H_{30}N_2O_3$ $[M+H]^+$, 467.2329; found, 467.2326.

Spectroscopic Data of **5z**

ethyl 3,8a-bis(3,5-dimethylphenyl)-8-oxo-8,8a-dihydroindeno[2,1-c]pyrazole-3a(3H)-carboxylate

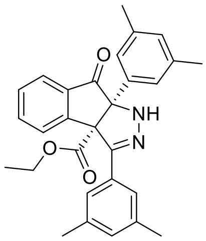

White solid; Mp: 217.5-218.3 °C; 352 mg, yield: 78%; > 95:5 dr;

IR (KBr): 3425, 3127, 3010, 2877, 1855, 1727, 1625, 1524, 1481, 1382, 1220, 821, 781, 617 cm^{-1};

^{1}H NMR (600 MHz, Chloroform-*d*) δ 7.83 (d, *J* = 7.6 Hz, 1H, ArH), 7.69 (d, *J* = 7.9 Hz, 1H, ArH), 7.57 – 7.53 (m, 1H, ArH), 7.38 (t, *J* = 7.5 Hz, 1H, ArH), 7.14 (s, 2H, ArH), 6.87 – 6.80 (m, 4H, ArH), 6.54 (s, 1H, NH), 3.75 (dq, *J* = 10.7, 7.1 Hz, 1H, CH_2), 3.66 (dq, *J* = 10.8, 7.1 Hz, 1H, CH_2), 2.21 (s, 6H, CH_3), 2.14 (s, 6H, CH_3), 0.75 (t, *J* = 7.1 Hz, 3H, CH_3).

^{13}C NMR (151 MHz, Chloroform-*d*) δ 203.65, 167.60, 150.78, 148.33, 136.88, 136.76, 135.51, 134.59, 134.50, 129.88, 129.64, 129.29, 127.92, 127.72, 123.70, 123.66, 123.50, 83.69, 72.06, 60.78, 20.31, 20.24, 12.30;

HRMS (TOF ES+): m/z calcd for $C_{29}H_{28}N_2O_3$ $[M+H]^+$, 453.2173; found, 453.2172.

Spectroscopic Data of **5aa**

methyl 3,8a-bis(3,5-dimethylphenyl)-8-oxo-8,8a-dihydroindeno[2,1-c]pyrazole-3a(3H)-carboxylate

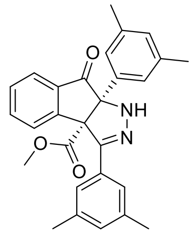

White solid; Mp: 227.7-228.5 °C; 359 mg, yield: 82%; > 95:5 dr;

IR (KBr): 3435, 3125, 3012, 2867, 1841, 1713, 1621, 1527, 1385, 1135, 827, 766, 617 cm^{-1};

^{1}H NMR (600 MHz, Chloroform-*d*) δ 7.85 (d, *J* = 7.6 Hz, 1H, ArH), 7.68 (d, *J* = 7.9 Hz, 1H, ArH), 7.57 (t, *J* = 7.6 Hz, 1H, ArH), 7.41 (t, *J* = 7.5 Hz, 1H, ArH), 7.13 (s, 2H, ArH), 6.89 – 6.80 (m, 4H, ArH), 6.54 (s, 1H, NH), 3.22 (s, 3H, CH_3), 2.22 (s, 6H, CH_3), 2.16 (s, 6H, CH_3).

^{13}C NMR (151 MHz, Chloroform-*d*) δ 203.67, 168.08, 150.46, 148.15, 136.97, 136.78, 135.56, 134.70, 134.39, 129.79, 129.73, 129.40, 128.01, 127.65, 123.70, 123.61, 123.41, 83.88, 72.23, 51.31, 20.35, 20.26;

HRMS (TOF ES+): m/z calcd for $C_{28}H_{26}N_2O_3$ $[M+H]^+$, 439.2016; found, 439.2014.

Spectroscopic Data of **5ab**

propyl 3,8a-bis(3,4-dimethylphenyl)-8-oxo-8,8a-dihydroindeno[2,1-c]pyrazole-3a(3H)-carboxylate

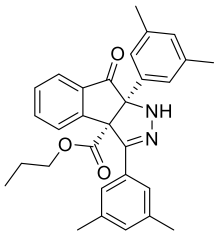

White solid; Mp: 253.1-254.2 °C; 256 mg, yield: 55%; > 95:5 dr;

IR (KBr): 3437, 3207, 3046, 2962, 2867, 1851, 1733, 1624, 1547, 1485, 1384, 1244, 863, 781, 663 cm^{-1};

^{1}H NMR (600 MHz, Chloroform-*d*) δ 7.85 (d, *J* = 7.7 Hz, 1H, ArH), 7.68 (d, *J* = 7.9 Hz, 1H, ArH), 7.58 (t, *J* = 7.6 Hz, 1H, ArH), 7.42 (t, *J* = 7.5 Hz, 1H, ArH), 7.15 (s, 2H, ArH), 6.88 (s, 1H, ArH), 6.83 (d, *J* = 6.1 Hz, 3H, ArH), 6.49 (s, 1H, NH), 3.71 (dt, *J* = 10.6, 6.7 Hz, 1H, CH_2), 3.42 (dt, *J* = 10.6, 6.5 Hz, 1H, CH_2), 2.23 (s, 6H, CH_3), 2.16 (s, 6H, CH_3), 1.24 (d, *J* = 7.3 Hz, 2H, CH_2), 0.61 (t, *J* = 7.4 Hz, 3H, CH_3).

^{13}C NMR (151 MHz, Chloroform-*d*) δ 204.01, 167.76, 150.81, 148.56, 136.70, 136.05, 135.77, 135.56, 135.46, 134.55, 131.90, 128.49, 127.87, 127.80, 127.55, 127.07, 127.00, 123.60, 123.26, 122.94, 83.75, 72.11, 66.41, 20.33, 18.76, 18.75, 18.59, 18.41, 9.24;

HRMS (TOF ES+): m/z calcd for $C_{30}H_{30}N_2O_3$ $[M+H]^+$, 467.2329; found, 467.2326.

Spectroscopic Data of **5ac**

methyl 3,8a-bis(2-methoxyphenyl)-8-oxo-8,8a-dihydroindeno[2,1-c]pyrazole-3a(3H)-carboxylate

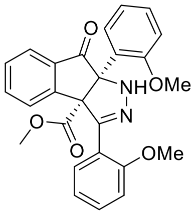

White solid; Mp: 205.2-206.3 °C; 336 mg, yield: 76%; > 95:5 dr;

IR (KBr): 3415, 3028, 2871, 2413, 1832, 1721, 1615, 1537, 1392, 1121, 755, 624 cm^{-1};

^{1}H NMR (500 MHz, Chloroform-*d*) δ 7.96 (dd, *J* = 7.8, 1.7 Hz, 1H, ArH), 7.77 (dd, *J* = 7.6, 1.1 Hz, 1H, ArH), 7.50 (dd, *J* = 7.8, 1.8 Hz, 1H, ArH), 7.46 – 7.39 (m, 2H,

ArH), 7.32 (ddd, *J* = 8.1, 6.6, 1.7 Hz, 1H, ArH), 7.21 – 7.15 (m, 2H, ArH), 6.96 (td, *J* = 7.6, 1.1 Hz, 1H, ArH), 6.84 – 6.76 (m, 2H, ArH), 6.64 (dd, *J* = 8.2, 1.1 Hz, 1H, ArH), 6.32 (s, 1H, NH), 3.70 (s, 3H, CH_3), 3.11 (d, *J* = 13.0 Hz, 6H, CH_3).

^{13}C NMR (126 MHz, Chloroform-*d*) δ 204.44, 167.47, 155.80, 154.71, 149.69, 146.97, 136.53, 133.63, 129.71, 129.43, 129.30, 128.77, 127.12, 125.55, 124.90, 122.48, 120.01, 119.67, 119.08, 110.52, 109.85, 81.89, 72.68, 53.99, 52.92, 50.33;

HRMS (TOF ES+): m/z calcd for $C_{26}H_{22}N_2O_5$ $[M+H]^+$, 443.1601; found, 443.1600.

Spectroscopic Data of **5ad**

ethyl 5,6-dimethoxy-8-oxo-3,8a-diphenyl-8,8a-dihydroindeno[2,1-c]pyrazole -3a(3H)-carboxylate

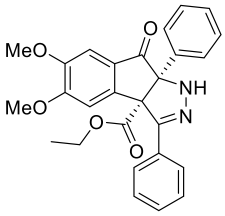

White solid; Mp: 234.4-235.1 °C; 387 mg, yield: 85%; > 95:5 dr;

IR (KBr): 3415, 3032, 3017, 2871, 1827, 1719, 1604, 1532, 1459, 1392, 1226, 828, 757, 615 cm^{-1};

^{1}H NMR (600 MHz, Chloroform-*d*) δ 7.54 – 7.46 (m, 2H, ArH), 7.31 – 7.20 (m, 9H, ArH), 7.07 (s, 1H, ArH), 6.53 (s, 1H, NH), 3.84 (s, 3H, OCH_3), 3.80 – 3.69 (m, 4H, CH_2 and OCH_3), 3.64 (dq, *J* = 10.7, 7.1 Hz, 1H, CH_2), 0.74 (t, *J* = 7.1 Hz, 3H, CH_3).

^{13}C NMR (151 MHz, Chloroform-*d*) δ 201.64, 167.86, 154.75, 149.57, 146.64, 135.11, 130.34, 128.80, 127.97, 127.61, 127.36, 127.28, 126.83, 125.89, 125.73, 108.91, 103.83, 83.91, 71.51, 60.93, 55.29, 55.21, 12.39;

HRMS (TOF ES+): m/z calcd for $C_{27}H_{24}N_2O_5$ $[M+H]^+$, 457.1758; found, 457.1759.

Spectroscopic Data of **5ae**

Ethyl 5,6-dimethoxy-8-oxo-3,8a-di(thiophen-3-yl)-8,8a-dihydroindeno[2,1-c]pyrazole -3a(3H)-carboxylate

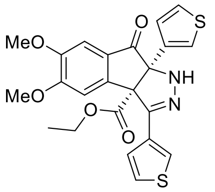

White solid; Mp: 217.8-218.4 °C; 398 mg, yield: 87%; > 95:5 dr;

IR (KBr): 3407, 3015, 2980, 2867, 2503, 1821, 1701, 1612, 1531, 1448, 1385, 1218,

835, 775, 638 cm^{-1};

^{1}H NMR (600 MHz, Chloroform-*d*) δ 7.36 (t, *J* = 3.1 Hz, 2H, ArH), 7.27 (dd, *J* = 5.1, 1.3 Hz, 1H, ArH), 7.23 (d, *J* = 4.5 Hz, 2H, ArH), 7.18 (dd, *J* = 5.1, 3.0 Hz, 1H, ArH), 7.07 (s, 1H, ArH), 6.77 (dd, *J* = 5.0, 1.3 Hz, 1H, ArH), 6.50 (s, 1H, NH), 3.86 (s, 3H, OCH_3), 3.84 – 3.75 (m, 5H, CH_2 and OCH_3), 0.85 (t, *J* = 7.1 Hz, 3H, CH_3).

^{13}C NMR (151 MHz, Chloroform-*d*) δ 200.29, 167.63, 154.95, 149.73, 145.85, 145.68, 135.63, 131.95, 127.87, 125.90, 124.99, 124.88, 123.21, 121.80, 108.58, 104.00, 81.51, 71.50, 61.08, 55.39, 55.24, 12.54;

HRMS (TOF ES+): m/z calcd for $C_{23}H_{20}N_2O_5S_2$ $[M+H]^+$, 469.0886; found, 469.0881.

Spectroscopic Data of **5o-*d*$_3$**

methyl-*d*$_3$ 8-oxo-3,8a-diphenyl-8,8a-dihydroindeno[2,1-c]pyrazole
-3a(3H)-carboxylate

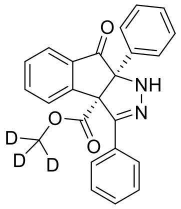

White solid; Mp: 213.5-214.3 °C; 45 mg, yield: 86%; > 95:5 dr;

^{1}H NMR (600 MHz, Chloroform-*d*) δ 7.86 (d, *J* = 7.7 Hz, 1H, ArH), 7.72 (d, *J* = 7.9 Hz, 1H, ArH), 7.58 (t, *J* = 7.6 Hz, 1H, ArH), 7.55 – 7.52 (m, 2H, ArH), 7.43 (t, *J* = 7.5 Hz, 1H, ArH), 7.28 – 7.22 (m, 8H, ArH), 6.60 (s, 1H, CH);

^{13}C NMR (151 MHz, Chloroform-*d*) δ 203.47, 168.04, 150.42, 148.06, 135.62, 134.77, 134.64, 129.93, 128.12, 127.99, 127.77, 127.75, 127.50, 127.31, 125.89, 125.61, 123.75, 84.00, 72.30, 13.08;

HRMS (TOF ES+): m/z calcd for $C_{24}H_{15}D_3N_2O_3$ $[M+H]^+$, 386.1578; found, 386.1578.

Supplementary Method 1

Gram-scale Reactions

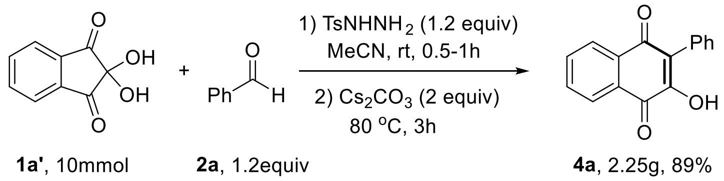

Gram-scale reaction A: A mixture of aldehydes (**2a**, 12 mmol) and ptoluenesulfonyl hydrazide (12 mmol) in acetonitrile (40 ml) was stirred at room temperature for 0.5-1h until complete consumption of starting materials (monitored by TLC). Then, 1,2,3-Indantrione Monohydrate (**1a'**, 10 mmol) and Cs_2CO_3 (2 equiv) was added to the crude product and stirred at 80°C for 3 h. After the reaction was finished, the solvent was removed under reduced pressure and the residue was purified by silica gel column chromatography (Petroleum ether/Ethyl acetate 8:1) to afford the desired product **4a** (2.25g, 89%).

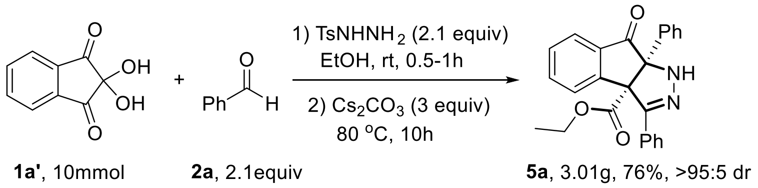

Gram-scale reaction B: A mixture of aldehydes (**2a**, 2.1equiv) and ptoluenesulfonyl hydrazide (2.1 equiv) in alcohol (60 ml) was stirred at room temperature for 0.5-1h until complete consumption of starting materials (monitored by TLC). Then, 1,2,3-Indantrione Monohydrate (**1a'**, 10 mmol) and Cs_2CO_3 (3 equiv) was added to the crude product and stirred at 80°C for 10 h. After the reaction was finished, the solvent was removed under reduced pressure and the residue was purified by silica gel column chromatography (Petroleum ether/Ethyl acetate 25:1) to afford the desired product **5a** (3.01g, 76%).

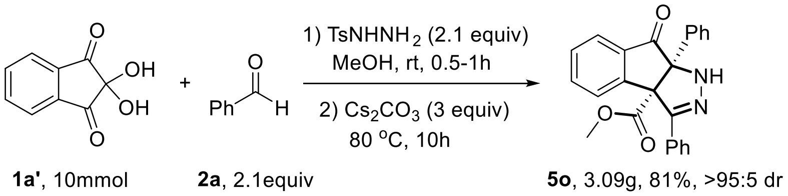

Gram-scale reaction C: A mixture of aldehydes (**2a**, 2.1equiv) and ptoluenesulfonyl hydrazide (2.1 equiv) in methanol (60 ml) was stirred at room temperature for 0.5-1h until complete consumption of starting materials (monitored by TLC). Then, 1,2,3-Indantrione Monohydrate (**1a'**, 10 mmol) and Cs_2CO_3 (3 equiv) was added to

the reaction mixture and stirred at 80°C in for 10 h. After the reaction was finished, the solvent was removed under reduced pressure and the residue was purified by silica gel column chromatography (Petroleum ether/Ethyl acetate 25:1) to afford the desired product **5o** (3.09g, 81%).

Supplementary Method 2

Synthetic Transformations

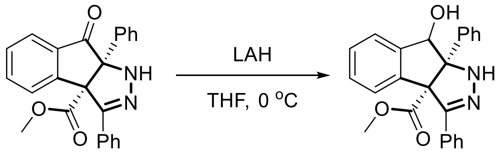

5o, >95:5 dr **6a**, >95:5 dr

Transformation A: A mixture of **5o** (0.5 mmol) and $LiAlH_4$ (3 equiv) in THF (10ml) was stirred at 0 °C for 10h until complete consumption of starting materials (monitored by TLC). After completion of the reaction, diluted with H_2O (20 mL) and compound was extracted with ethyl acetate (3 x 20 mL). The combined organic layers were dried (Na_2SO_4) and concentrated. the residue was purified by silica gel column chromatography (Petroleum ether/Ethyl acetate 10:1) to afford the desired product **6a**. **methyl 8-hydroxy-3,8a-diphenyl-8,8a-dihydroindeno[2,1-c]pyrazole-3a(3*H*)-carboxylate: ^{1}H NMR** (600 MHz, DMSO-*d*$_6$) δ 8.06 (s, 1H, NH), 7.63 (d, *J* = 7.5 Hz, 1H, ArH), 7.56 – 7.53 (m, 2H, ArH), 7.39 (d, *J* = 7.4 Hz, 1H, ArH), 7.36 – 7.31 (m, 4H, ArH), 7.26 (t, *J* = 7.3 Hz, 1H, ArH), 7.19 (s, 5H, ArH), 5.67 (d, *J* = 5.2 Hz, 1H, CH), 5.28 (d, *J* = 5.2 Hz, 1H, OH), 3.14 (s, 3H, CH_3); **^{13}C NMR** (151 MHz, DMSO-*d*$_6$) δ 169.64, 146.78, 145.84, 136.92, 135.88, 132.43, 128.93, 128.55, 128.43, 128.22, 128.19, 128.15, 127.82, 127.28, 126.25, 122.71, 92.28, 84.18, 74.65, 52.19; **HRMS** (TOF ES+): m/z calcd for $C_{24}H_{20}N_2O_3$ $[M+H]^+$, 385.1547; found, 385.1553.

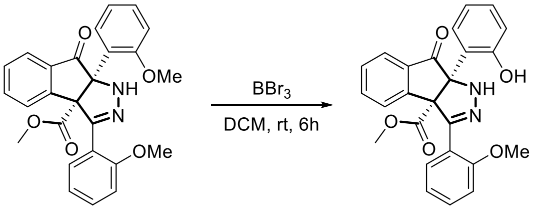

5n, >95:5 dr **6b**, >95:5 dr

Transformation B: A mixture of **5n** (0.5 mmol) and BBr_3(3 equiv) in DCM (10ml) was stirred at room temperature for 6h until complete consumption of starting materials (monitored by TLC). After completion of the reaction, diluted with H_2O (20 mL) and compound was extracted with ethyl acetate (3 x 20 mL). The combined organic layers were dried (Na_2SO_4) and concentrated. the residue was purified by silica gel column chromatography (Petroleum ether/Ethyl acetate 10:1) to afford the desired product **6b**. **methyl 8a-(2-hydroxyphenyl)-3-(2-methoxyphenyl)-8-oxo-8, 8a -dihydroindeno [2,1-c]pyrazole-3a(1H)-carboxylate: ^{1}H NMR** (600 MHz,

DMSO-d_6) δ 10.78 (s, 1H, NH), 9.07 (s, 1H, OH), 7.89 (dd, *J* = 7.6, 1.1 Hz, 1H, ArH), 7.81 (d, *J* = 7.9 Hz, 1H, ArH), 7.77 – 7.74 (m, 1H, ArH), 7.66 (dd, *J* = 7.7, 1.8 Hz, 1H, ArH), 7.59 (t, *J* = 7.4 Hz, 1H, ArH), 7.41 (dd, *J* = 7.9, 1.6 Hz, 1H, ArH), 7.35 (td, *J* = 7.8, 1.7 Hz, 1H, ArH), 7.23 – 7.20 (m, 1H, ArH), 7.07 (t, *J* = 7.6 Hz, 1H, ArH), 6.92 (t, *J* = 8.5 Hz, 3H, ArH), 3.25 (s, 3H, CH_3), 3.13 (s, 3H, CH_3); **^{13}C NMR** (151 MHz, DMSO-d_6) δ 199.43, 168.68, 156.77, 156.47, 148.65, 146.40, 136.09, 135.72, 130.39, 130.06, 129.79, 129.59, 127.94, 127.09, 125.60, 124.54, 120.91, 119.82, 117.29, 115.80, 111.87, 81.75, 73.56, 55.52, 52.49; **HRMS** (TOF ES+): m/z calcd for $C_{25}H_{20}N_2O_5$ $[M+H]^+$, 429.1445; found, 429.1451.

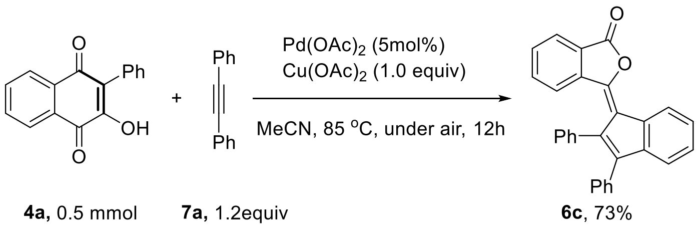

Transformation C: A mixture of 2-hydroxy-3-phenylnaphthalene-1,4-dione (**4a**, 0.5 mmol), alkyne (**7a**, 0.6 mmol), $Pd(OAc)_2$ (5.0 mol %) and $Cu(OAc)_2$ (1.0 equiv) in MeCN (20mL) was stirred at 85 °C under open air for 12 hours. The solvent was removed under vacuo and the crude product thus obtained was purified by silica gel (100-200 mesh) column chromatography using EtOAc/Hexane as the eluant to afford **6c. (E)-3-(2,3-diphenyl-1H-inden-1-ylidene)isobenzofuran-1(3H)-one: ^{1}H NMR** (500 MHz, Chloroform-*d*) δ 8.47 (d, *J* = 7.6 Hz, 1H), 7.83 (d, *J* = 7.6 Hz, 1H), 7.32 (t, *J* = 7.5 Hz, 1H), 7.28 – 7.18 (m, 11H), 7.14 (d, *J* = 7.2 Hz, 2H), 7.02 (t, *J* = 7.9 Hz, 1H), 5.64 (d, *J* = 8.2 Hz, 1H); **^{13}C NMR** (126 MHz, Chloroform-*d*) δ 165.12, 146.73, 144.64, 141.02, 136.69, 135.73, 135.02, 133.74, 133.31, 132.63, 129.73, 129.19, 128.49, 127.98, 127.17, 127.10, 126.59, 126.46, 126.27, 125.81, 125.77, 125.08, 124.87, 124.35, 119.72; **HRMS** (TOF ES+): m/z calcd for $C_{29}H_{19}O_2$ $[M+H]^+$, 399.1380; found, 399.1383.

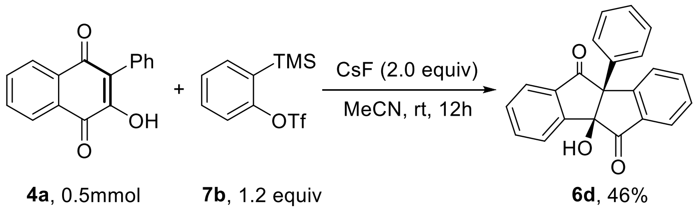

Transformation D: A mixture of 2-hydroxy-3-phenylnaphthalene-1,4-dione (**4a**, 0.5 mmol), 2-(Trimethylsilyl)phenyl trifluoromethanesulfonate (**7b**, 0.6 mmol), CsF (2 equiv) in MeCN (20mL) was stirred at room temperature for 12 hours. After completion of the reaction, diluted with H_2O (20 mL) and compound was extracted with ethyl acetate (3 x 20 mL). The combined organic layers were dried (Na_2SO_4) and concentrated. Pure product **6d** was obtained by column chromatography (silica gel, mixture of EtOAc/Hexane).

(4bS,9bR)-4b-hydroxy-9b-phenyl-4b,9b-dihydroindeno[2,1-a]indene-5,10-dione: **^{1}H NMR** (600 MHz, Chloroform-*d*) δ 7.85 (d, *J* = 7.8 Hz, 1H), 7.73 (dd, *J* = 7.8, 4.3 Hz, 2H), 7.66 (p, *J* = 7.5 Hz, 3H), 7.48 (dt, *J* = 19.7, 7.4 Hz, 2H), 7.25 (p, *J* = 3.7 Hz, 3H), 6.91 – 6.86 (m, 2H), 2.74 (s, 1H, OH); **^{13}C NMR** (151 MHz, Chloroform-*d*) δ 201.49, 200.89, 150.65, 149.82, 136.80, 135.68, 135.36, 133.40, 131.81, 130.06, 129.02, 128.48, 127.65, 127.08, 126.65, 124.97, 124.10, 123.56, 84.66, 69.35; **HRMS** (TOF ES+): m/z calcd for $C_{22}H_{15}O_3$ $[M+H]^+$, 327.1016; found, 327.1015.

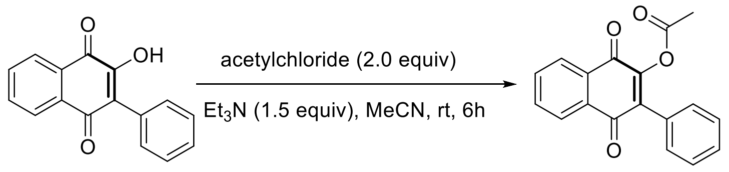

4a, 1mmol **6e**, 93%

Transformation E: A mixture of 2-hydroxy-3-phenylnaphthalene-1,4-dione (**4a**, 1 mmol), acetylchloride (2 equiv) and Et_3N (1.5 equiv) in MeCN (20mL) was stirred at room temperature for 6 hours. After completion of the reaction, diluted with H_2O (20 mL) and compound was extracted with ethyl acetate (3 x 20 mL). Crude product thus obtained was purified by silica gel (100-200 mesh) column chromatography using EtOAc/Hexane as the eluant to afford **6e.** **1,4-dioxo-3-phenyl-1,4-dihydronaphthalen-2-yl acetate:** **^{1}H NMR** (500 MHz, Chloroform-*d*) δ 8.08 – 8.02 (m, 2H), 7.67 (td, *J* = 6.7, 6.1, 3.8 Hz, 2H), 7.38 – 7.33 (m, 3H), 7.26 (dd, *J* = 6.9, 3.0 Hz, 2H), 2.14 (s, 3H); **^{13}C NMR** (126 MHz, Chloroform-*d*) δ 183.88, 178.65, 168.29, 150.21, 137.41, 134.44, 134.05, 131.98, 130.85, 129.78, 129.49, 129.12, 128.14, 127.13, 126.60, 20.41; **HRMS** (TOF ES+): m/z calcd for $C_{18}H_{13}O_4$ $[M+H]^+$, 293.0808; found, 293.0809.

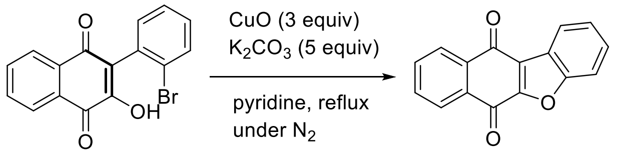

4a, 0.54mmol **6f**, 75%

Benzofuronaphthoquinone

Transformation F: A mixture of 2-hydroxy-3-phenylnaphthalene-1,4-dione (**4a**, 0.54 mmol), CuO (3 equiv) and K_2CO_3 (5 equiv) in dry deoxygenated pyridine (20 mL) was refluxed under N_2 for 4 h. The mixture was then added to 20% aq HCl solution (50 mL) and the resulting suspension was extracted with CH_2Cl_2 (3 x 30 mL). The combined organic layers were washed with 10% aq NaOH (3 x 30 mL), dried, filtered and concentrated in vacuo. The solid residue was submitted to flash column chromatography (eluant: ethyl acetate/hexane, 1:9) to give the title compound **6f** (100 mg, 75% yield) as yellow crystals. **naphtho[2,3-b]benzofuran-6,11-dione:** **^{1}H NMR** (600 MHz, Chloroform-*d*) δ 8.24 (d, *J* = 7.9 Hz, 1H), 8.17 (d, *J* = 17.5 Hz, 2H), 7.72 (dt, *J* = 5.9, 2.6 Hz, 2H), 7.63 (d, *J* = 8.4 Hz, 1H), 7.55 – 7.49 (m, 1H), 7.43 (t, *J* = 7.6

Hz, 1H); **^{13}C NMR** (151 MHz, Chloroform-*d*) δ 180.41, 174.48, 155.43, 152.51, 133.21, 132.85, 132.30, 131.36, 128.61, 125.90, 125.80, 125.07, 123.23, 123.01, 121.72, 111.87; **HRMS** (TOF ES+): m/z calcd for $C_{16}H_9O_3$ $[M+H]^+$, 249.0546; found, 249.0547

Supplementary Note 3

X-ray Structure and Data of Products

X-ray Structure and Data of **4h**

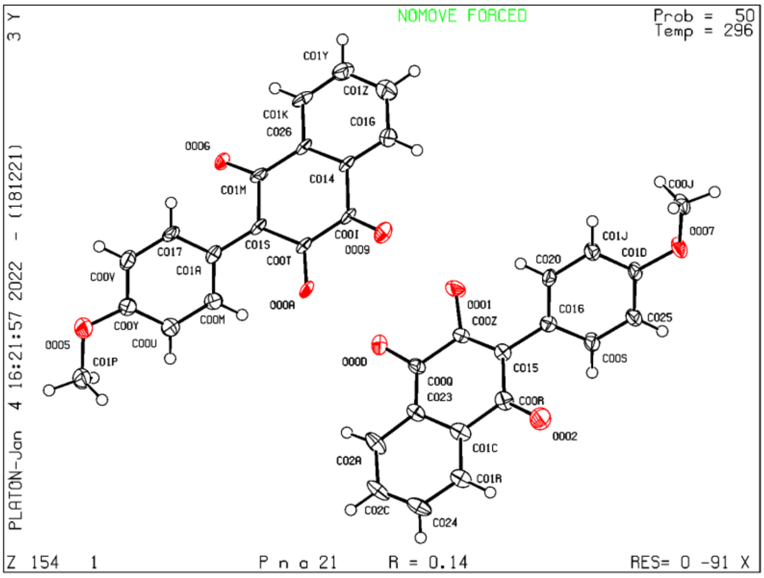

Figure S1. X-Ray crystal structure of 4h

Datablock: 1

Bond precision:	C-C = 0.0145 A		Wavelength=0.71073
Cell:	a=25.4601(16)	b=3.8772(3)	c=26.2598(16)
	alpha=90	beta=90	gamma=90
Temperature:	296 K		

	Calculated	Reported
Volume	2592.2(3)	2592.2(3)
Space group	P n a 21	P n a 21
Hall group	P 2c -2n	P 2c -2n
Moiety formula	C17 H11 O4	C17 H11 O4
Sum formula	C17 H11 O4	C17 H11 O4
Mr	279.26	279.26
Dx,g cm-3	1.431	1.431
Z	8	8
Mu (mm-1)	0.103	0.103
F000	1160.0	1160.0
F000'	1160.66	
h,k,lmax	30,4,31	30,4,31
Nref	4605[2358]	4581
Tmin,Tmax	0.987,0.990	
Tmin'	0.987	

Correction method= Not given

Data completeness= 1.94/0.99 Theta(max)= 25.024

R(reflections)= 0.1390(4332) wR2(reflections)= 0.3428(4581)

S = 1.225 Npar= 334

Table S1. Crystal data and structure refinement for 4h

X-ray Structure and Data of **4ae**

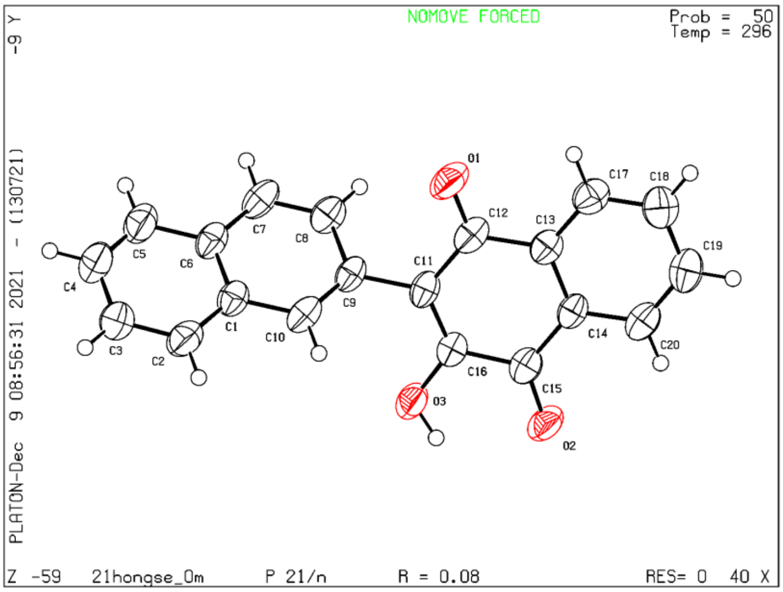

Figure S2. X-Ray crystal structure of **4ae**

Datablock: 21hongse_0m

Bond precision:	C-C = 0.0056 A		Wavelength=0.71073
Cell:	a=6.984(4)	b=6.878(4)	c=29.840(16)
	alpha=90	beta=96.585(7)	gamma=90
Temperature:	296 K		

	Calculated	Reported
Volume	1423.9(14)	1423.8(13)
Space group	P 21/n	P 21/n
Hall group	-P 2yn	-P 2yn
Moiety formula	C20 H12 O3	?
Sum formula	C20 H12 O3	C20 H12 O3
Mr	300.30	300.30
Dx,g cm-3	1.401	1.401
Z	4	4
Mu (mm-1)	0.094	0.094
F000	624.0	624.0
F000'	624.32	
h,k,lmax	9,8,38	8,8,38
Nref	3265	3098
Tmin,Tmax	0.983,0.991	
Tmin'	0.983	

Correction method= Not given

Data completeness= 0.949 Theta(max)= 27.513

R(reflections)= 0.0781(1390) wR2(reflections)= 0.2413(3098)

S = 0.907 Npar= 210

Table S2. Crystal data and structure refinement for **4ae**

X-ray Structure and Data of **5a**

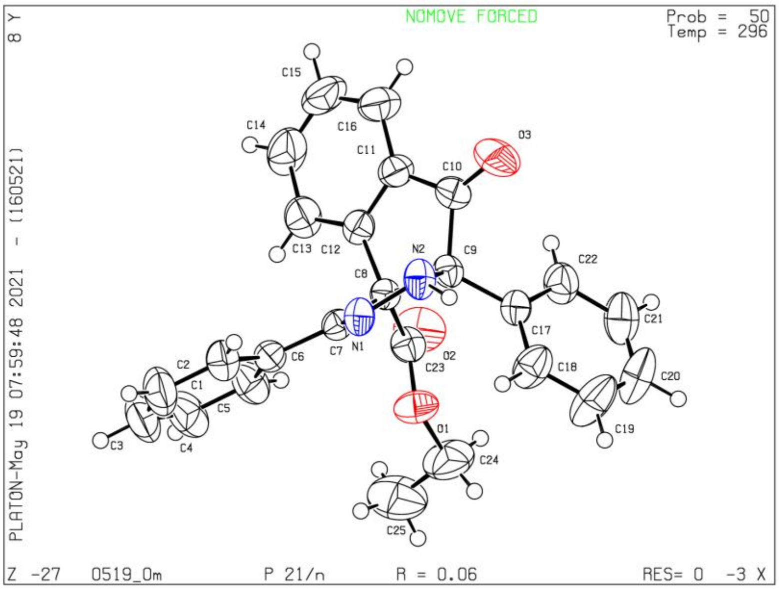

Figure S3. X-Ray crystal structure of **5a**

Datablock: 0519_0m

Bond precision: C-C = 0.0037 A Wavelength=0.71073

Cell:	a=11.574(3)	b=14.784(4)	c=13.021(3)
	alpha=90	beta=110.118(4)	gamma=90
Temperature:	296 K		

	Calculated	Reported
Volume	2092.1(9)	2092.0(9)
Space group	P 21/n	P 21/n
Hall group	-P 2yn	-P 2yn
Moiety formula	C25 H20 N2 O3	?
Sum formula	C25 H20 N2 O3	C25 H20 N2 O3
Mr	396.43	396.43
Dx,g cm-3	1.259	1.259
Z	4	4
Mu (mm-1)	0.083	0.083
F000	832.0	832.0
F000’	832.37	
h,k,lmax	15,19,16	15,19,16
Nref	4831	4802
Tmin,Tmax	0.985,0.988	
Tmin’	0.985	

Correction method= Not given

Data completeness= 0.994 Theta(max)= 27.549

R(reflections)= 0.0594(2689) wR2(reflections)= 0.1951(4802)

S = 1.007 Npar= 276

Table S3. Crystal data and structure refinement for **5a**

X-ray Structure and Data of **5n**

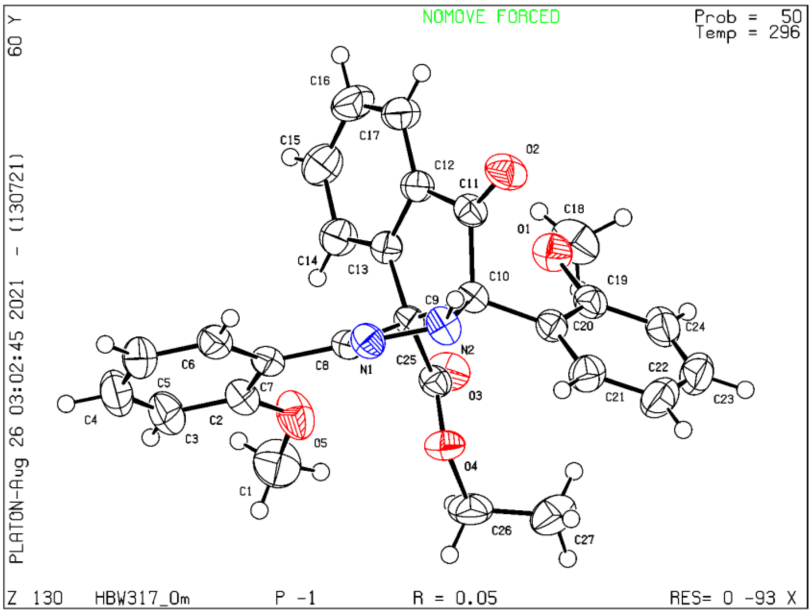

Figure S4. X-Ray crystal structure of **5n**

Datablock: HBW317_0m

Bond precision: C-C = 0.0026 A Wavelength=0.71073

Cell:	a=8.806(4)	b=11.419(5)	c=12.136(5)
	alpha=101.824(5)	beta=109.470(5)	gamma=91.079(5)
Temperature:	296 K		

	Calculated	Reported
Volume	1121.1(8)	1121.0(8)
Space group	P -1	P -1
Hall group	-P 1	-P 1
Moiety formula	C27 H24 N2 O5	?
Sum formula	C27 H24 N2 O5	C27 H24 N2 O5
Mr	456.48	456.48
Dx,g cm-3	1.352	1.352
Z	2	2
Mu (mm-1)	0.094	0.094
F000	480.0	480.0
F000'	480.24	
h,k,lmax	11,14,15	11,14,15
Nref	5248	5091
Tmin,Tmax	0.983,0.986	
Tmin'	0.983	

Correction method= Not given

Data completeness= 0.970 Theta(max)= 27.665

R(reflections)= 0.0473(3633) wR2(reflections)= 0.1432(5091)

S = 1.001 Npar= 314

Table S4. Crystal data and structure refinement for **5n**

X-ray Structure and Data of **5q**

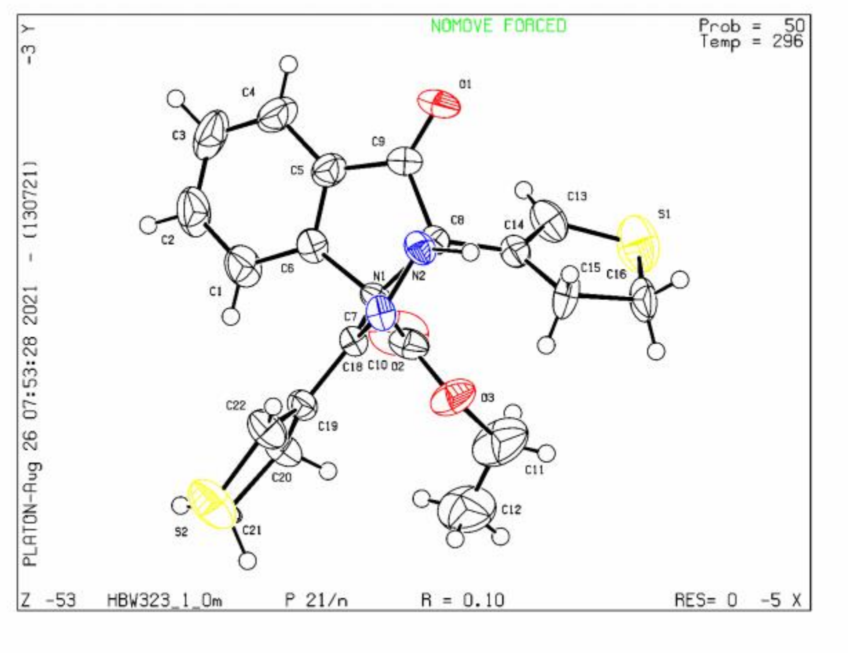

Figure S5. X-Ray crystal structure of **5q**

Datablock: HBW323_1_0m

Bond precision:	C-C = 0.0061 A		Wavelength=0.71073
Cell:	a=10.924(7)	b=14.835(10)	c=12.719(8)
	alpha=90	beta=108.221(9)	gamma=90
Temperature:	296 K		

	Calculated	Reported
Volume	1958(2)	1958(2)
Space group	P 21/n	P 21/n
Hall group	-P 2yn	-P 2yn
Moiety formula	C21 H20 N2 O3 S2	?
Sum formula	C21 H20 N2 O3 S2	C21 H20 N2 O3 S2
Mr	412.51	412.51
Dx,g cm-3	1.399	1.399
Z	4	4
Mu (mm-1)	0.297	0.297
F000	864.0	864.0
F000'	865.33	
h,k,lmax	14,19,16	14,19,16
Nref	4463	4395
Tmin,Tmax	0.948,0.956	
Tmin'	0.948	

Correction method= Not given

Data completeness= 0.985 Theta(max)= 27.443

R(reflections)= 0.1031(3033) wR2(reflections)= 0.3340(4395)

S = 1.507 Npar= 253

Table S5. Crystal data and structure refinement for **5q**

X-ray Structure and Data of **5r**

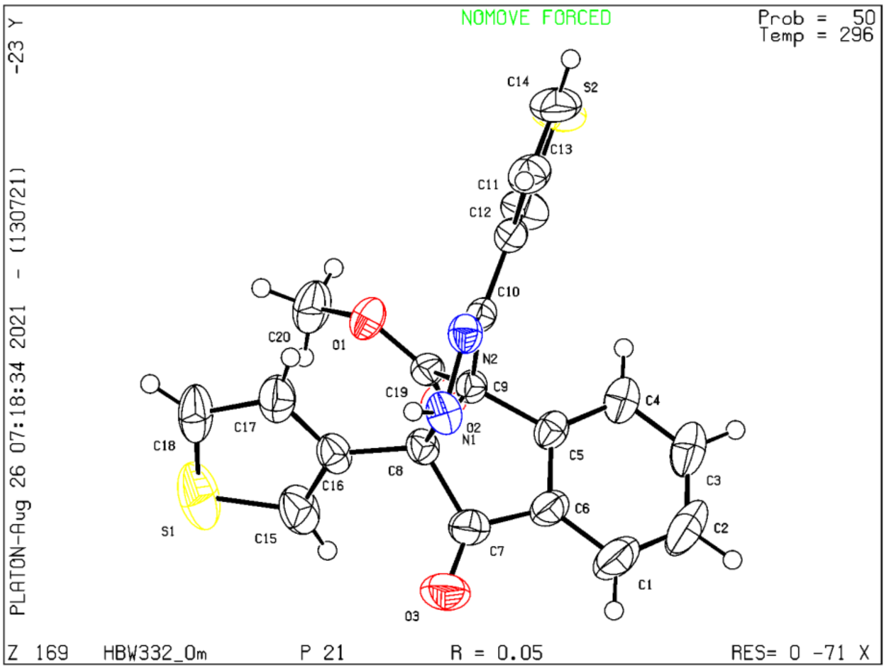

Figure S6. X-Ray crystal structure of **5r**

Datablock: HBW332_0m

Bond precision:	C-C = 0.0066 A		Wavelength=0.71073
Cell:	a=8.181(10)	b=13.952(16)	c=8.984(11)
	alpha=90	beta=116.337(14)	gamma=90
Temperature:	296 K		

	Calculated	Reported
Volume	919.0(19)	918.9(19)
Space group	P 21	P 21
Hall group	P 2yb	P 2yb
Moiety formula	C20 H14 N2 O3 S2	?
Sum formula	C20 H14 N2 O3 S2	C20 H14 N2 O3 S2
Mr	394.45	394.45
Dx,g cm-3	1.426	1.426
Z	2	2
Mu (mm-1)	0.313	0.313
F000	408.0	408.0
F000'	408.66	
h,k,lmax	10,18,11	10,18,11
Nref	4243[2208]	3287
Tmin,Tmax	0.951,0.957	
Tmin'	0.951	

Correction method= Not given

Data completeness= 1.49/0.77 Theta(max)= 27.537

R(reflections)= 0.0468(2792) wR2(reflections)= 0.1418(3287)

S = 0.978 Npar= 249

Table S6. Crystal data and structure refinement for **5r**
