# Supplementary material for: Switchable synthesis of natural-product-like lawsones and indenopyrazoles through regioselective ring-expansion of indantrione
Source: Commun Chem. 2023 Jan 18;6:17. doi: 10.1038/s42004-022-00807-z (PMC9849474; doi:10.1038/s42004-022-00807-z)
Supplement: Supplementary file 3 — Description of Additional Supplementary Files [file 42004_2022_807_MOESM3_ESM.pdf]

Description of Additional Supplementary Files

File name: Supplementary Data 1

Description: X-ray data file of compound **4h** (CCDC 2149716)

File name: Supplementary Data 2

Description: X-ray data file of compound **4ae** (CCDC 2149715)

File name: Supplementary Data 3

Description: X-ray data file of compound **5a** (CCDC 2149717)

File name: Supplementary Data 4

Description: X-ray data file of compound **5n** (CCDC 2149718)

File name: Supplementary Data 5

Description: X-ray data file of compound **5q** (CCDC 2149719)

File name: Supplementary Data 6

Description: X-ray data file of compound 5r (CCDC 2149720)

File name: Supplementary Data 7

Description: ^{1}H NMR and ^{13}C NMR Copies of Products

File name: Supplementary Data 8

Description: DFT calculation data
