# Supplementary material for: Switchable synthesis of natural-product-like lawsones and indenopyrazoles through regioselective ring-expansion of indantrione
Source: Commun Chem. 2023 Jan 18;6:17. doi: 10.1038/s42004-022-00807-z (PMC9849474; doi:10.1038/s42004-022-00807-z)
Supplement: Supplementary file 10 — Supplementary Data 7 [file 42004_2022_807_MOESM10_ESM.zip › Supplementary Data 7.doc]

**^1^H NMR and ^13^C NMR Copies of Products:**

# ^1^H-NMR spectrum of 4a

^13^C-NMR spectrum of 4a

# ^1^H-NMR spectrum of 4b

 ^13^C-NMR spectrum of 4b

# ^19^F-NMR spectrum of 4b

# ^1^H-NMR spectrum of 4c

 ^13^C-NMR spectrum of 4c

# ^1^H-NMR spectrum of 4d

^13^C-NMR spectrum of 4d

## ^1^H-NMR spectrum of 4e

 ^13^C-NMR spectrum of 4e

# ^1^H-NMR spectrum of 4f

 ^13^C-NMR spectrum of 4f

# ^1^H-NMR spectrum of 4g

 ^13^C-NMR spectrum of 4g

# ^1^H-NMR spectrum of 4h

 ^13^C-NMR spectrum of 4h

# ^1^H-NMR spectrum of 4i

 ^13^C-NMR spectrum of 4i

# ^1^H-NMR spectrum of 4j

^13^C-NMR spectrum of 4j

# ^19^F-NMR spectrum of 4j

# ^1^H-NMR spectrum of 4k

# ^13^C-NMR spectrum of 4k

# ^1^H-NMR spectrum of 4l

 ^13^C-NMR spectrum of 4l

# ^1^H-NMR spectrum of 4m

 ^13^C-NMR spectrum of 4m

# ^19^F-NMR spectrum of 4m

# ^1^H-NMR spectrum of 4n

 ^13^C-NMR spectrum of 4n

# ^1^H-NMR spectrum of 4o

 ^13^C-NMR spectrum of 4o

# ^1^H-NMR spectrum of 4p

 ^13^C-NMR spectrum of 4p

# ^1^H-NMR spectrum of 4q

 ^13^C-NMR spectrum of 4q

# ^1^H-NMR spectrum of 4r

# ^13^C-NMR spectrum of 4r

# ^19^F-NMR spectrum of 4r

# ^1^H-NMR spectrum of 4s

^13^C-NMR spectrum of 4s

# ^1^H-NMR spectrum of 4t

^13^C-NMR spectrum of 4t

# ^1^H-NMR spectrum of 4u

 ^13^C-NMR spectrum of 4u

# ^19^F-NMR spectrum of 4u

# ^1^H-NMR spectrum of 4v

 ^13^C-NMR spectrum of 4v

# ^1^H-NMR spectrum of 4w

 ^13^C-NMR spectrum of 4w

# ^1^H-NMR spectrum of 4x

 ^13^C-NMR spectrum of 4x

# ^1^H-NMR spectrum of 4y

 ^13^C-NMR spectrum of 4y

# ^1^H-NMR spectrum of 4z

# ^13^C-NMR spectrum of 4z

# ^19^F-NMR spectrum of 4z

# ^1^H-NMR spectrum of 4aa

 ^13^C-NMR spectrum of 4aa

# ^1^H-NMR spectrum of 4ab

 ^13^C-NMR spectrum of 4ab

# ^1^H-NMR spectrum of 4ac

^13^C-NMR spectrum of 4ac

# ^1^H-NMR spectrum of 4ad

 ^13^C-NMR spectrum of 4ad

# ^1^H-NMR spectrum of 4ae

 ^13^C-NMR spectrum of 4ae

# ^1^H-NMR spectrum of 4af

^13^C-NMR spectrum of 4af

# ^1^H-NMR spectrum of 4ag

^13^C-NMR spectrum of 4ag

# ^1^H-NMR spectrum of 4ah

^13^C-NMR spectrum of 4ah

# ^1^H-NMR spectrum of 4ai

^13^C-NMR spectrum of 4ai

# ^1^H-NMR spectrum of 4aj

^13^C-NMR spectrum of 4aj

# ^1^H-NMR spectrum of 4ak

^13^C-NMR spectrum of 4ak

# ^1^H-NMR spectrum of 4al + 4al’

^13^C-NMR spectrum of 4al + 4al’

# ^1^H-NMR spectrum of 4am + 4am’

 ^13^C-NMR spectrum of 4am + 4am’

^1^H-NMR spectrum of 5a

^13^C-NMR spectrum of 5a

# ^1^H-NMR spectrum of 5b

^13^C-NMR spectrum of 5b

# ^1^H-NMR spectrum of 5c

^13^C-NMR spectrum of 5c

# ^19^F-NMR spectrum of 5c

# ^1^H-NMR spectrum of 5d

^13^C-NMR spectrum of 5d

# ^1^H-NMR spectrum of 5e

 ^13^C-NMR spectrum of 5e

# ^1^H-NMR spectrum of 5f

^13^C-NMR spectrum of 5f

# ^1^H-NMR spectrum of 5g

^13^C-NMR spectrum of 5g

# ^1^H-NMR spectrum of 5h

^13^C-NMR spectrum of 5h

# ^1^H-NMR spectrum of 5i

^13^C-NMR spectrum of 5i

# ^19^F-NMR spectrum of 5i

# ^1^H-NMR spectrum of 5j

^13^C-NMR spectrum of 5j

# ^1^H-NMR spectrum of 5k

^13^C-NMR spectrum of 5k

# ^1^H-NMR spectrum of 5l

^13^C-NMR spectrum of 5l

# ^1^H-NMR spectrum of 5m

^13^C-NMR spectrum of 5m

# ^19^F-NMR spectrum of 5m

# ^1^H-NMR spectrum of 5n

^13^C-NMR spectrum of 5n

# ^1^H-NMR spectrum of 5o

^13^C-NMR spectrum of 5o

# ^1^H-NMR spectrum of 5p

^13^C-NMR spectrum of 5p

# ^1^H-NMR spectrum of 5q

^13^C-NMR spectrum of 5q

# ^1^H-NMR spectrum of 5r

^13^C-NMR spectrum of 5r

# ^1^H-NMR spectrum of 5s

^13^C-NMR spectrum of 5s

# ^1^H-NMR spectrum of 5t

^13^C-NMR spectrum of 5t

# ^1^H-NMR spectrum of 5u

^13^C-NMR spectrum of 5u

# ^1^H-NMR spectrum of 5v

^13^C-NMR spectrum of 5v

# ^1^H-NMR spectrum of 5w

^13^C-NMR spectrum of 5w

# ^1^H-NMR spectrum of 5x

^13^C-NMR spectrum of 5x

# ^1^H-NMR spectrum of 5y

^13^C-NMR spectrum of 5y

# ^1^H-NMR spectrum of 5z

^13^C-NMR spectrum of 5z

# ^1^H-NMR spectrum of 5aa

^13^C-NMR spectrum of 5aa

# ^1^H-NMR spectrum of 5ab

^13^C-NMR spectrum of 5ab

# ^1^H-NMR spectrum of 5ac

^13^C-NMR spectrum of 5ac

# ^1^H-NMR spectrum of 5ad

^13^C-NMR spectrum of 5ad

# ^1^H-NMR spectrum of 5ae

^13^C-NMR spectrum of 5ae

# ^1^H-NMR spectrum of 5o-d_3_

#  ^13^C-NMR spectrum of 5o-d_3_

# ^1^H-NMR spectrum of 6a

^13^C-NMR spectrum of 6a

# ^1^H-NMR spectrum of 6b

#  ^13^C-NMR spectrum of 6b

# ^1^H-NMR spectrum of 6c

^13^C-NMR spectrum of 6c

# ^1^H-NMR spectrum of 6d

# ^13^C-NMR spectrum of 6d

# ^1^H-NMR spectrum of 6e

^13^C-NMR spectrum of 6e

# ^1^H-NMR spectrum of 6f

^13^C-NMR spectrum of 6f
