# Supplementary material for: Switchable synthesis of natural-product-like lawsones and indenopyrazoles through regioselective ring-expansion of indantrione
Source: Commun Chem. 2023 Jan 18;6:17. doi: 10.1038/s42004-022-00807-z (PMC9849474; doi:10.1038/s42004-022-00807-z)
Supplement: Supplementary file 11 — Supplementary Data 8 [file 42004_2022_807_MOESM11_ESM.zip › Supplementary Data 8.doc]

# 1. **Theoretical calculations on the reaction pathways**

Computational Methods**:** All DFT calculations were performed with Gaussian 16 package.^[1]^ The geometry optimizations of minima and transition states were carried out at the B3LYP-D3(BJ) functional^[2]^ with a mixed basis set of Lanl2DZ for Cs and 6-31G(d) for other atoms. The vibrational frequencies were computed at the same level to check whether each optimized structure is an energy minimum (zero imaginary frequency) or a transition state (one imaginary frequency) and to evaluate its zero-point vibration energy (ZPE) and thermal corrections at 353.15 K in kcal‧mol^-1^. The single-point energies and solvent effects in acetonitrile and ethanol were computed with the B3LYP-D3(BJ) function with a mixed basis set of Lanl2DZ for Cs and 6-311++G(d,p) for other atoms using the SMD solvation model.^[3]^ DFT-optimized structures were illustrated using CYLView.^[4]^ The computed output file is in the open database (<https://zenodo.org/>) link (DOI:10.5281/zenodo.7296393).

**Table S7**. Electronic energies, enthalpies and free energies (in hartrees) computed at the level of B3LYP-D3(BJ)-SMD(acetonitrile)/6-311++G(d,p)//B3LYP-D3(BJ)/6-31G(d)

| **Structure** | **ZPE** | **ΔH_0→T_** | **ΔG_0→T_** | **E** | **H** | **G** | **Imaginary** |
| --- | --- | --- | --- | --- | --- | --- | --- |
|  |  |  |  |  |  |  | **Frequency** |
| **1a** | 0.108128 | 0.121265 | 0.065219 | -571.222908 | -571.101643 | -571.157689 |  |
| **b** | 0.114973 | 0.126075 | 0.075079 | -379.938914 | -379.812839 | -379.863835 |  |
| **N_2_** | 0.005598 | 0.009513 | -0.016918 | -109.555117 | -109.545604 | -109.572035 |  |
| **INT-1** | 0.224168 | 0.249652 | 0.163766 | -951.175516 | -950.925864 | -951.01175 |  |
| **TS-1** | 0.224584 | 0.248165 | 0.166558 | -951.143223 | -951.13371 | -950.976665 | -462.43*i* |
| **INT-2** | 0.227975 | 0.250897 | 0.170897 | -951.160957 | -950.912792 | -950.99006 |  |
| **TS-2** | 0.223758 | 0.24761 | 0.166541 | -951.139371 | -950.888474 | -950.97283 | -353.35*i* |
| **INT-3** | 0.218186 | 0.238291 | 0.163081 | -841.691764 | -841.444154 | -841.528683 |  |
| **4b** | 0.21855 | 0.239697 | 0.164911 | -841.700147 | -841.461856 | -841.535236 |  |
| **TS-3** | 0.22506 | 0.248479 | 0.168317 | -951.154862 | -950.906383 | -950.986545 | -447.37*i* |
| **INT-4** | 0.228376 | 0.251205 | 0.171126 | -951.171237 | -950.920032 | -951.000111 |  |
| **TS-4** | 0.22453 | 0.248229 | 0.166842 | -951.1531 | -950.904871 | -950.986258 | -388.62*i* |
| **INT-5** | 0.218664 | 0.239671 | 0.164294 | -841.704058 | -841.464387 | -841.539764 |  |
| **4a** | -841.46091 | 0.24009 | 0.166237 | -841.713494 | -841.473404 | -841.547257 |  |

**Table S8**. Electronic energies, enthalpies and free energies (in hartrees) computed at the level of B3LYP-D3(BJ)-SMD(ethanol)/[other:6-311++G(d,p);Cs: Lanl2DZ] //B3LYP-D3(BJ)/[other:6-31G(d); Cs: Lanl2DZ]

| **Structure** | **ZPE** | **ΔH_0→T_** | **ΔG_0→T_** | **E** | **H** | **G** | **Imaginary Frequency** |
| --- | --- | --- | --- | --- | --- | --- | --- |
|  |  |  |  |  |  |  |  |
| **1a** | 0.108128 | 0.121265 | 0.065219 | -571.218864 | -571.097599 | -571.153645 |  |
| **EtO^-^** | 0.064168 | 0.070198 | 0.033656 | -154.599268 | -154.529070 | -154.565612 |  |
| **CsCO_3_** | 0.015360 | 0.025590 | -0.030552 | -303.855413 | -303.829823 | -303.885965 |  |
| **Cs_2_HCO_3_^+^** | 0.028249 | 0.039169 | -0.018837 | -304.357284 | -304.318115 | -304.376121 |  |
| **b** | 0.114973 | 0.126075 | 0.075079 | -379.937289 | -379.811214 | -379.862210 |  |
| **N_2_** | 0.005598 | 0.009513 | -0.016918 | -109.555378 | -109.545865 | -109.572296 |  |
| **H_2_O** | 0.021167 | 0.025648 | -0.000517 | -76.472253 | -76.446605 | -76.472770 |  |
| **INT-6** | 0.178226 | 0.196992 | 0.127684 | -725.854484 | -725.657492 | -725.726800 |  |
| **TS-5** | 0.176813 | 0.195423 | 0.125982 | -725.812643 | -725.617220 | -725.686661 | -86.37*i* |
| **INT-7** | 0.176582 | 0.196286 | 0.123325 | -725.836383 | -725.640097 | -725.713058 |  |
| **INT-8** | 0.293308 | 0.324960 | 0.222669 | -1105.778395 | -1105.453435 | -1105.555726 |  |
| **TS-6** | 0.293548 | 0.323495 | 0.227848 | -1105.743662 | -1105.420167 | -1105.515814 | -330.16 |
| **INT-9** | 0.320039 | 0.341375 | 0.240319 | -1300.718913 | -1300.377538 | -1300.478594 |  |
| **TS-7** | 0.315209 | 0.352873 | 0.236632 | -1300.706038 | -1300.584773 | -1300.469406 | -1105.06*i* |
| **INT-10** | 0.319412 | 0.358220 | 0.237852 | -1300.730398 | -1300.372178 | -1300.492546 |  |
| **TS-8** | 0.316991 | 0.355619 | 0.235671 | -1300.712583 | -1300.356964 | -1300.476912 | -128.17*i* |
| **INT-11** | 0.275398 | 0.300594 | 0.215821 | -920.366162 | -920.065568 | -920.150341 |  |
| **INT-12** | 0.392079 | 0.429412 | 0.316008 | -1300.318984 | -1299.889572 | -1300.002976 |  |
| **TS-9** | 0.392473 | 0.427878 | 0.321728 | -1300.298958 | -1299.871080 | -1299.977230 | -340.1*i* |
| **INT-13** | 0.395915 | 0.430798 | 0.323392 | -1300.347672 | -1299.916874 | -1300.024280 |  |
| **5a** | 0.397135 | 0.431936 | 0.326635 | -1300.365179 | -1299.933243 | -1300.038544 |  |

2. **Calculated total energies and geometrical coordinates for all the structures calculated in this work:**

**1a(acetonitrile)**

E(RB3LYP) = -571.222908 Hartree

Sum of electronic and thermal Free Energies = -571.157689 Hartree

C	0.41761200	0.70505000	-0.00004100

C	0.41761200	-0.70505000	-0.00004100

C	1.61469200	-1.42107900	-0.00001700

C	2.80874100	-0.70358500	0.00001900

C	2.80874100	0.70358500	0.00001900

C	1.61469200	1.42107900	-0.00001700

C	-0.96358500	1.24023300	-0.00000100

C	-1.89407400	0.00000000	-0.00000800

C	-0.96358500	-1.24023300	-0.00000100

H	1.59880300	-2.50621300	0.00000000

H	3.75651500	-1.23367100	0.00002600

H	3.75651600	1.23367100	0.00002600

H	1.59880300	2.50621200	0.00000000

O	-1.31801600	2.39992700	0.00003700

O	-1.31801600	-2.39992700	0.00003700

O	-3.09842900	0.00000000	-0.00001400

**b(acetonitrile)**

E(RB3LYP) =-379.938914 Hartree

Sum of electronic and thermal Free Energies =-379.863835Hartree

C	-2.54671400	0.50321100	-0.00000600

C	-2.25574200	-0.86200000	-0.00008900

C	-0.93531100	-1.30203600	0.00022600

C	0.12656900	-0.38008300	0.00006600

C	-0.17464300	0.99416300	-0.00009300

C	-1.49734800	1.42526300	0.00009000

H	-3.57723700	0.84509900	-0.00001500

H	-3.06123300	-1.59118400	0.00004900

H	-0.71721000	-2.36687900	0.00048500

H	0.62965800	1.72474300	-0.00036900

H	-1.70982100	2.49079300	-0.00013000

C	1.49225200	-0.88473500	-0.00002800

H	1.72110400	-1.94285800	-0.00000400

N	2.51912400	-0.08800700	-0.00077400

N	3.40378500	0.64194800	0.00062900

**N_2_(acetonitrile)**

E(RB3LYP) =-109.555117 Hartree

Sum of electronic and thermal Free Energies = -109.572035 Hartree

N	0.00000000	0.00000000	0.55274900

N	0.00000000	0.00000000	-0.55274900

**INT-1**

E(RB3LYP) = -951.175516 Hartree

Sum of electronic and thermal Free Energies = -951.01175 Hartree

C	1.43001500	-0.61036500	-0.91594900

C	1.61773100	-0.93690500	0.44047500

C	2.81315100	-0.63255600	1.09116200

C	3.81710800	-0.00099600	0.36186600

C	3.62962200	0.32517200	-0.99411600

C	2.43559600	0.02539500	-1.64419900

C	0.08481300	-1.01667800	-1.38012200

C	-0.60361100	-1.66659100	-0.15707100

C	0.41669800	-1.59667200	1.00448800

H	2.94002600	-0.88790700	2.13832100

H	4.75944000	0.24638100	0.84185900

H	4.43069700	0.81857400	-1.53678500

H	2.27388600	0.27450600	-2.68802500

O	-0.40101400	-0.88680400	-2.48586500

O	0.25576900	-2.02053700	2.13004900

O	-1.70992300	-2.14698500	-0.12764800

C	0.70890900	2.23412900	1.21033900

C	0.48612200	2.52531700	-0.13867500

C	-0.65199400	2.05742300	-0.78472900

C	-1.60396400	1.28033600	-0.09405800

C	-1.36340700	0.98070000	1.26312400

C	-0.22432800	1.46253200	1.90365100

H	1.60428500	2.59215600	1.70823900

H	1.21115600	3.11255200	-0.69468800

H	-0.80987700	2.27505200	-1.83701800

H	-2.06430400	0.35893000	1.81180800

H	-0.05634100	1.20597900	2.94533800

C	-2.76842900	0.80160100	-0.81040400

H	-2.86956300	0.89223800	-1.88362400

N	-3.71898100	0.14426200	-0.20819300

N	-4.55562600	-0.40781800	0.34070500

**TS-1**

E(RB3LYP) = -951.143223 Hartree

Sum of electronic and thermal Free Energies = -950.976665 Hartree

C	1.80859800	-0.32125700	0.07041400

C	1.58085700	0.99737800	0.50528400

C	2.44333500	2.03519900	0.14092500

C	3.53972300	1.73574500	-0.66225700

C	3.77531600	0.41461800	-1.07999300

C	2.91691800	-0.62375300	-0.71488400

C	0.73020400	-1.25596700	0.55808900

C	-0.08065000	-0.40776300	1.55761400

C	0.41034800	1.06056900	1.40397300

H	2.25261300	3.04323600	0.49648500

H	4.22675600	2.52180700	-0.96107200

H	4.64508200	0.19942700	-1.69462600

H	3.10246600	-1.64810400	-1.02382400

O	0.93846800	-2.50284800	0.77757200

O	-0.09022200	2.03307600	1.93146100

O	-0.92011600	-0.79887100	2.32964600

C	-3.51358900	1.77642400	-0.53790200

C	-2.36115200	2.06363200	-1.26878300

C	-1.39851700	1.07656300	-1.46660200

C	-1.57471800	-0.20101400	-0.91521000

C	-2.73283700	-0.48258300	-0.17457700

C	-3.69909600	0.50069200	0.00230100

H	-4.26486700	2.54496100	-0.38386200

H	-2.21156300	3.05400100	-1.68748200

H	-0.49796000	1.29742600	-2.03294400

H	-2.86004900	-1.46101000	0.27778100

H	-4.59193800	0.27757700	0.57792200

C	-0.46135700	-1.16194800	-1.01865100

H	0.21900500	-0.95629600	-1.84712200

N	-0.74768400	-2.53554000	-1.02733400

N	-0.35872800	-3.45918600	-0.46254200

**INT-2**

E(RB3LYP) = -951.160957 Hartree

Sum of electronic and thermal Free Energies = -950.99006 Hartree

C	-1.71051700	0.34417600	-0.14529600

C	-1.83189000	-0.90775700	0.48008800

C	-2.88831300	-1.76982400	0.17339600

C	-3.82854900	-1.35669000	-0.76450900

C	-3.71592800	-0.09711600	-1.37680000

C	-2.65892200	0.76270100	-1.07445000

C	-0.46101600	1.06499600	0.28202000

C	0.02506600	0.23360500	1.49588800

C	-0.76982400	-1.09863500	1.48566100

H	-2.96062900	-2.73056300	0.67360100

H	-4.66078400	-2.00429500	-1.02303800

H	-4.46682900	0.21308400	-2.09764800

H	-2.58214600	1.73887500	-1.54358500

O	-0.68468400	2.42795900	0.66028500

O	-0.54281400	-2.06184800	2.18624300

O	0.83236400	0.58167000	2.31721600

C	3.62596400	-1.86408400	-0.64454700

C	2.43672000	-2.08977200	-1.33825000

C	1.48050000	-1.07883900	-1.42629600

C	1.70457500	0.15827400	-0.81256100

C	2.89430400	0.37962500	-0.11340000

C	3.85346200	-0.62816600	-0.03648100

H	4.37285800	-2.64950500	-0.57630300

H	2.25410300	-3.04890800	-1.81361100

H	0.55478400	-1.25085100	-1.97014900

H	3.05959700	1.33515100	0.37279600

H	4.77641700	-0.45022000	0.50712800

C	0.60962200	1.19153300	-0.84515700

H	0.11227900	1.16189400	-1.82336400

N	1.07402300	2.58446800	-0.67495400

N	0.37024800	3.22109300	0.09615400

**TS-2**

E(RB3LYP) = -951.139371 Hartree

Sum of electronic and thermal Free Energies = -950.97283 Hartree

C	-1.49107000	0.03457400	-0.47096900

C	-1.62153900	-0.68108500	0.72554700

C	-2.39530800	-1.84636900	0.78095600

C	-3.02573800	-2.28239900	-0.37781900

C	-2.90535200	-1.54719000	-1.57032800

C	-2.14604900	-0.37890600	-1.62688700

C	-0.61220600	1.31068700	-0.30647900

C	-0.42577700	1.33952200	1.25870400

C	-0.96124500	0.00526000	1.84526300

H	-2.49690400	-2.37446300	1.72418400

H	-3.62685700	-3.18644900	-0.36436700

H	-3.41909400	-1.89256600	-2.46302300

H	-2.07695900	0.20638400	-2.53773600

O	-1.17497100	2.39153100	-0.81875500

O	-0.80912700	-0.36710500	2.99095900

O	0.01916400	2.25824700	1.89607600

C	3.29360600	-2.24671400	0.18355000

C	2.78360700	-2.21881600	-1.11662600

C	1.94543600	-1.18063200	-1.50689400

C	1.58202800	-0.16950900	-0.59648200

C	2.12058700	-0.19836900	0.70508500

C	2.96828100	-1.23176000	1.08749800

H	3.95327800	-3.05331500	0.48964800

H	3.04349600	-3.00128900	-1.82288200

H	1.54322400	-1.15478000	-2.51575300

H	1.90326100	0.59973400	1.40398000

H	3.37913800	-1.24366700	2.09197900

C	0.64004900	0.84852100	-1.03966900

H	0.47862200	0.86983000	-2.11750500

N	1.43242300	2.63835900	-1.05384700

N	0.64506400	3.45140000	-0.98636300

**INT-3**

E(RB3LYP) = -841.691764 Hartree

Sum of electronic and thermal Free Energies = -841.528683 Hartree

C	-1.09701800	-0.55740000	-0.73106300

C	-1.80931400	0.11327500	0.28360700

C	-3.02514300	-0.40778400	0.75495500

C	-3.54275600	-1.57761800	0.21729200

C	-2.84157600	-2.24108800	-0.79620100

C	-1.62963300	-1.73532000	-1.26130700

C	0.33262300	1.45498800	-1.17352100

C	-0.14479500	2.08942200	0.14725200

C	-1.30518600	1.37404900	0.88254800

H	-3.54150400	0.13384500	1.54048900

H	-4.48698900	-1.97413100	0.57755700

H	-3.24118100	-3.15616100	-1.22362500

H	-1.08237800	-2.26212100	-2.03791200

O	0.77405400	2.12999800	-2.07518100

O	-1.79335300	1.89409300	1.86812300

O	0.33308600	3.11398400	0.56985500

C	3.65451500	-1.36263200	1.15225900

C	2.36627400	-1.56024300	1.64575800

C	1.25614400	-1.16123000	0.89861400

C	1.42922800	-0.56561900	-0.35424600

C	2.72752300	-0.36198600	-0.84232200

C	3.83269800	-0.76112400	-0.09617000

H	4.51626800	-1.67187800	1.73637700

H	2.21879300	-2.02562400	2.61596300

H	0.25896800	-1.32739500	1.29096900

H	2.86446800	0.12108600	-1.80604600

H	4.83312100	-0.59939500	-0.48673100

C	0.25252300	-0.06774100	-1.20516100

H	0.41350100	-0.37452500	-2.24318300

**4b**

E(RB3LYP) = -841.700147 Hartree

Sum of electronic and thermal Free Energies = -841.535236 Hartree

C	0.70260200	-0.78588000	0.06011200

C	2.09758100	-0.54836300	0.01411400

C	3.00563000	-1.60867300	0.06290100

C	2.55557500	-2.92229600	0.16462600

C	1.18298500	-3.16660500	0.22631200

C	0.26795200	-2.11431200	0.17768400

C	0.15437200	1.62510400	-0.04616800

C	1.58935300	1.99151300	-0.07769100

C	2.61718000	0.83363700	-0.06603800

H	4.06401400	-1.37250400	0.02330500

H	3.26382500	-3.74409300	0.20196900

H	0.81549000	-4.18492200	0.31682700

H	-0.79298100	-2.32717000	0.23647300

O	-0.67019600	2.69545300	-0.10464200

O	3.80356500	1.10409700	-0.11614100

O	1.96832000	3.14484800	-0.12440400

C	-4.49289900	-0.41867400	-0.04479600

C	-3.69460500	-0.83832800	-1.11167700

C	-2.32115800	-0.60610700	-1.09333100

C	-1.72214200	0.04559400	-0.00342600

C	-2.53371900	0.46308600	1.06380100

C	-3.91049300	0.23285700	1.04187700

H	-5.56362800	-0.59929600	-0.06206500

H	-4.14410500	-1.34217400	-1.96244800

H	-1.70095200	-0.92531500	-1.92563500

H	-2.07561600	0.95400400	1.91820400

H	-4.52390100	0.55777700	1.87715800

C	-0.25922100	0.32661300	0.00707300

H	-1.58960700	2.37351400	-0.11452400

**TS-3**

E(RB3LYP) = -951.154862 Hartree

Sum of electronic and thermal Free Energies = -950.986545 Hartree

C	0.72675600	-1.75920300	-0.16457400

C	0.80859000	-0.92432100	0.96139500

C	2.04078400	-0.53782500	1.48184800

C	3.19397200	-1.01154600	0.85707900

C	3.11349800	-1.85335300	-0.26513200

C	1.87832200	-2.23446500	-0.78911600

C	-0.68724900	-1.94172800	-0.59149900

C	-1.53007600	-1.04956100	0.34262200

C	-0.54934000	-0.51609300	1.40106200

H	2.08454400	0.12049800	2.34344100

H	4.17050300	-0.72819800	1.23917300

H	4.02909000	-2.20730300	-0.73032100

H	1.79999900	-2.87626000	-1.66118700

O	-1.08583700	-2.61352300	-1.52204800

O	-0.83866000	0.13388900	2.38587500

O	-2.74666800	-1.29373900	0.63848300

C	1.76617300	3.09171700	-0.32056700

C	1.77062800	2.15562500	-1.35553700

C	0.65112500	1.35856000	-1.57741400

C	-0.47681700	1.47708300	-0.75208600

C	-0.47501600	2.41737300	0.28936900

C	0.63936100	3.22426200	0.49502000

H	2.63701400	3.71760300	-0.14942800

H	2.64302500	2.04816300	-1.99286300

H	0.65514300	0.62380300	-2.37762600

H	-1.33458700	2.49591300	0.94699100

H	0.63286700	3.95053300	1.30202400

C	-1.56554500	0.49852100	-0.91867300

H	-1.60835700	0.02808400	-1.90333500

N	-2.87221300	0.85687100	-0.56140600

N	-3.72363400	0.37796700	0.04738500

**INT-4**

E(RB3LYP) = -951.171237 Hartree

Sum of electronic and thermal Free Energies = -951.000111 Hartree

C	2.11712000	-0.19940500	-0.40842400

C	1.62530000	0.34228400	0.78843900

C	2.27369900	1.40343100	1.41764500

C	3.42704000	1.90935700	0.82011600

C	3.92040400	1.36595000	-0.37961400

C	3.26979300	0.30599500	-1.00873600

C	1.23505500	-1.28854600	-0.88647700

C	0.05179900	-1.38177500	0.09774300

C	0.39932400	-0.37334000	1.21514300

H	1.88092700	1.81168300	2.34320900

H	3.95712500	2.73526900	1.28523100

H	4.82258500	1.78152800	-0.81861800

H	3.63749500	-0.12326700	-1.93518500

O	1.35744100	-1.95010300	-1.89619600

O	-0.21136200	-0.23206000	2.25285000

O	-0.09635600	-2.68891200	0.65035800

C	-2.85697900	2.84500200	-0.31611300

C	-2.04647800	2.48647600	-1.39376100

C	-1.56940800	1.18052200	-1.50014600

C	-1.89074100	0.23004100	-0.52620000

C	-2.69877100	0.59390300	0.55524700

C	-3.18371600	1.89628300	0.65490900

H	-3.23504500	3.86000000	-0.23398300

H	-1.79232800	3.21842100	-2.15488600

H	-0.94552800	0.89561800	-2.34419500

H	-2.93625100	-0.14160100	1.31607400

H	-3.81387200	2.17165400	1.49539100

C	-1.29996000	-1.14948800	-0.63132500

H	-1.17061100	-1.42109400	-1.68754800

N	-2.14144500	-2.22347000	-0.05544000

N	-1.49329400	-3.03649400	0.58225800

**TS-4**

E(RB3LYP) = -951.1531 Hartree

Sum of electronic and thermal Free Energies = -950.986258 Hartree

C	-1.98190500	0.25139400	0.71406500

C	-1.91990400	0.08336300	-0.67259900

C	-2.84263000	0.70566700	-1.51054200

C	-3.83190700	1.49530900	-0.92541800

C	-3.89273400	1.66256900	0.46850300

C	-2.96547000	1.04347700	1.30552300

C	-0.89992700	-0.47935700	1.41112800

C	-0.12438500	-1.31312300	0.25235700

C	-0.75467800	-0.75215500	-1.05252800

H	-2.77892300	0.56835400	-2.58516900

H	-4.56791500	1.99045800	-1.55212900

H	-4.67432900	2.28324200	0.89659200

H	-2.99757700	1.16142000	2.38368500

O	-0.67334600	-0.51332500	2.59303700

O	-0.40524400	-1.01426000	-2.18751300

O	-0.50846300	-2.57806400	0.42809400

C	3.34472400	2.60884800	-0.34403900

C	3.36813700	2.07289100	0.94543400

C	2.72140800	0.86889700	1.20456000

C	2.01774900	0.20041000	0.18540500

C	2.01009700	0.74338600	-1.11319600

C	2.67426300	1.93817600	-1.37066800

H	3.85750000	3.54368600	-0.55165900

H	3.89519700	2.58818000	1.74264600

H	2.73633200	0.44481400	2.20465200

H	1.51052300	0.21120600	-1.91318000

H	2.67357900	2.34669700	-2.37674000

C	1.31357500	-1.03276100	0.53016500

H	1.58690000	-1.43498700	1.50574200

N	1.95561800	-2.54196900	-0.42207400

N	1.19247800	-3.37800700	-0.51968100

**INT-5**

E(RB3LYP) = -841.704058 Hartree

Sum of electronic and thermal Free Energies = -841.539764 Hartree

C	-1.48523900	-0.62251100	-0.45125700

C	-1.63124600	0.49404200	0.39795100

C	-2.61158300	0.48489500	1.39714400

C	-3.43753000	-0.62289400	1.55321700

C	-3.29965600	-1.72725800	0.70290800

C	-2.33066800	-1.72572500	-0.29566200

C	-0.43596700	-0.66615800	-1.50932100

C	0.12297700	1.72453800	-1.01685400

C	-0.80507400	1.71425900	0.21503500

H	-2.70748600	1.35957200	2.03171600

H	-4.19435600	-0.62935300	2.33193600

H	-3.95034900	-2.58828000	0.82359100

H	-2.20390500	-2.57001700	-0.96469100

O	-0.42490400	-1.52186600	-2.37670500

O	-0.85424100	2.67930600	0.95460400

O	0.37302900	2.75643300	-1.59645200

C	3.59129200	-1.01632500	1.50215700

C	3.03568700	-1.90300700	0.57811500

C	2.09868700	-1.45448600	-0.35041000

C	1.70877900	-0.10921800	-0.37021500

C	2.27703100	0.77852700	0.55126000

C	3.21129200	0.32453900	1.48346600

H	4.31832100	-1.36821800	2.22821600

H	3.33101400	-2.94833600	0.57891100

H	1.67287900	-2.14814700	-1.06830900

H	2.00624500	1.82983800	0.54438100

H	3.64201600	1.02561200	2.19228900

C	0.68739800	0.37147800	-1.41296500

H	1.15746400	0.45815800	-2.39703200

**4a**

E(RB3LYP) = -841.713494 Hartree

Sum of electronic and thermal Free Energies = -841.547257 Hartree

H	1.73751800	2.21364500	-0.34789800

C	-1.80219100	-0.82006400	0.07780800

C	-2.32009900	0.48042500	-0.03089900

C	-3.70206500	0.68885400	-0.02147900

C	-4.56590300	-0.39714100	0.09672800

C	-4.05125200	-1.69313500	0.20450100

C	-2.67368800	-1.90457100	0.19428100

C	-0.32859000	-1.06561700	0.07282800

C	0.06140700	1.35858600	-0.15560100

C	-1.41041500	1.64384500	-0.16453400

H	-4.07315400	1.70438800	-0.10876900

H	-5.63991800	-0.23642500	0.10448700

H	-4.72659900	-2.53878400	0.29643800

H	-2.25008800	-2.89956500	0.27607300

O	0.11075700	-2.21037400	0.14222100

O	-1.81617000	2.79008900	-0.27825500

O	0.79674800	2.47157700	-0.31175400

C	4.82963800	-0.48598900	0.05892700

C	4.03324200	-1.23253400	-0.81304100

C	2.65270400	-1.05573700	-0.83461100

C	2.04148500	-0.12283100	0.01857700

C	2.85082800	0.61341400	0.90224400

C	4.23483100	0.43507500	0.91963800

H	5.90633300	-0.62816300	0.07121900

H	4.49106600	-1.95717000	-1.48023400

H	2.03805400	-1.64185100	-1.50722100

H	2.38750900	1.30130100	1.60528700

H	4.84247600	1.00818400	1.61393900

C	0.57608700	0.10144000	-0.01553700+

**1a(ethanol)**

E(RB3LYP) = -571.218864 Hartree

Sum of electronic and thermal Free Energies = -571.153645 Hartree

C	0.41761200	0.70505000	-0.00004100

C	0.41761200	-0.70505000	-0.00004100

C	1.61469200	-1.42107900	-0.00001700

C	2.80874100	-0.70358500	0.00001900

C	2.80874100	0.70358500	0.00001900

C	1.61469200	1.42107900	-0.00001700

C	-0.96358500	1.24023300	-0.00000100

C	-1.89407400	0.00000000	-0.00000800

C	-0.96358500	-1.24023300	-0.00000100

H	1.59880300	-2.50621300	0.00000000

H	3.75651500	-1.23367100	0.00002600

H	3.75651600	1.23367100	0.00002600

H	1.59880300	2.50621200	0.00000000

O	-1.31801600	2.39992700	0.00003700

O	-1.31801600	-2.39992700	0.00003700

O	-3.09842900	0.00000000	-0.00001400

**EtOH**

E(RB3LYP) = -155.112076 Hartree

Sum of electronic and thermal Free Energies = -155.062855 Hartree

C	1.22173300	-0.22203400	0.00004400

C	-0.08674300	0.54915700	-0.00004000

H	1.28676100	-0.86059200	0.88693500

H	1.28676200	-0.86075200	-0.88673300

H	2.07517300	0.46494100	-0.00003200

H	-0.13874300	1.20085600	-0.88759700

H	-0.13884500	1.20089300	0.88747500

O	-1.14947000	-0.39984100	-0.00009000

H	-1.98528600	0.09064600	0.00065000

**EtO^-^**

E(RB3LYP) = -154.599268 Hartree

Sum of electronic and thermal Free Energies = -154.565612 Hartree

C	1.18134900	-0.20067500	0.00005200

C	-0.23495900	0.47938200	-0.00003500

H	1.27315100	-0.84933700	0.88499000

H	1.27320600	-0.84943400	-0.88480700

H	2.02492100	0.51773400	0.00004000

H	-0.16972000	1.21799200	-0.89122200

H	-0.16978100	1.21808200	0.89108200

O	-1.23876500	-0.36591000	-0.00002400

**Cs_2_CO_3_**

E(RB3LYP) = -303.855413 Hartree

Sum of electronic and thermal Free Energies = -303.885965 Hartree

C	0.00008200	1.22095200	0.00004300

O	0.00012400	-0.11615100	0.00023400

O	-1.12419800	1.85159000	-0.00006400

O	1.12416900	1.85183000	0.00001800

Cs	2.86860100	-0.32749800	-0.00001800

Cs	-2.86862400	-0.32748100	-0.00001400

**Cs_2_HCO_3_^+^**

E(RB3LYP) = -304.357284 Hartree

Sum of electronic and thermal Free Energies = -304.376121 Hartree

C	0.10063900	1.20281200	0.00023900

O	-0.05957700	-0.04690200	0.00081400

O	-1.10806800	1.93391300	0.00001600

O	1.14569000	1.87800400	-0.00012300

Cs	-3.05135600	-0.37053700	-0.00006000

Cs	3.05864100	-0.36040400	-0.00005500

H	-0.82884100	2.86476200	-0.00074700

**b(ethanol)**

E(RB3LYP) = -379.937289 Hartree

Sum of electronic and thermal Free Energies = -379.862210 Hartree

C	-2.54671400	0.50321100	-0.00000600

C	-2.25574200	-0.86200000	-0.00008900

C	-0.93531100	-1.30203600	0.00022600

C	0.12656900	-0.38008300	0.00006600

C	-0.17464300	0.99416300	-0.00009300

C	-1.49734800	1.42526300	0.00009000

H	-3.57723700	0.84509900	-0.00001500

H	-3.06123300	-1.59118400	0.00004900

H	-0.71721000	-2.36687900	0.00048500

H	0.62965800	1.72474300	-0.00036900

H	-1.70982100	2.49079300	-0.00013000

C	1.49225200	-0.88473500	-0.00002800

H	1.72110400	-1.94285800	-0.00000400

N	2.51912400	-0.08800700	-0.00077400

N	3.40378500	0.64194800	0.00062900

**N_2_(ethanol)**

E(RB3LYP) = -109.555378 Hartree

Sum of electronic and thermal Free Energies = -109.572296 Hartree

N	0.00000000	0.00000000	0.55274900

N	0.00000000	0.00000000	-0.55274900

**H_2_O**

E(RB3LYP) = -76.472253 Hartree

Sum of electronic and thermal Free Energies = -76.472770 Hartree

O	0.00000000	0.00000000	0.11972000

H	0.00000000	0.76154500	-0.47888000

H	0.00000000	-0.76154500	-0.47888000

**INT-6**

E(RB3LYP) = -725.854484 Hartree

Sum of electronic and thermal Free Energies = -725.726800 Hartree

C	0.74271500	-0.59532400	0.04610700

C	1.53505100	0.55826100	-0.06260400

C	2.92384300	0.48138600	-0.20680600

C	3.51905800	-0.77566000	-0.25287200

C	2.72659000	-1.93476800	-0.15246000

C	1.34374500	-1.85274400	0.00470500

C	-0.71398600	-0.25107900	0.36663800

C	-0.78101500	1.25975900	0.03062200

C	0.68970800	1.76738700	-0.01152700

H	3.50852100	1.39483100	-0.28379600

H	4.59661800	-0.86909100	-0.37027700

H	3.20714000	-2.91057800	-0.20437200

H	0.72680500	-2.74323500	0.08214700

O	-0.92527100	-0.32585100	1.66212900

O	1.05611700	2.93080400	-0.09831200

O	-1.73962600	2.00314700	-0.09210900

C	-3.88957600	-0.43435200	-0.64330300

C	-2.84238100	-1.34855100	-0.00833900

H	-3.64947500	0.60719100	-0.41229800

H	-3.89378900	-0.55021100	-1.73490100

H	-4.89668300	-0.66941700	-0.26766200

H	-3.07515200	-2.40225700	-0.23118500

H	-2.82591900	-1.22064500	1.08038900

O	-1.54379300	-1.09340800	-0.51158400

**TS-5**

E(RB3LYP) = -725.812643 Hartree

Sum of electronic and thermal Free Energies = -725.686661 Hartree

C	0.77361700	-0.62716100	-0.45917300

C	1.62679500	0.34664700	0.08502400

C	2.95981600	0.10592800	0.44609900

C	3.46247600	-1.18095100	0.28073300

C	2.63937200	-2.18237200	-0.26303000

C	1.32326300	-1.90271100	-0.64938200

C	-1.20207000	0.27418100	0.17925400

C	-0.49387300	1.47224700	-0.40541400

C	0.94876500	1.64828600	0.13412000

H	3.57739500	0.91592900	0.83063600

H	4.48885200	-1.41433900	0.55882700

H	3.04105000	-3.19033800	-0.38200200

H	0.72751300	-2.70720100	-1.08721400

O	-1.23582900	0.04596200	1.39309500

O	1.41300400	2.73874900	0.43800100

O	-0.98815200	2.25833600	-1.19970100

C	-4.09354700	-0.90894800	0.62285600

C	-2.87417900	-1.34651200	-0.18254300

H	-3.76279800	-0.35666000	1.50521000

H	-4.73656500	-0.25826500	0.01899800

H	-4.68247100	-1.77922000	0.94418400

H	-3.17766000	-1.92528100	-1.06343300

H	-2.21234900	-1.96306600	0.43516300

O	-2.14971900	-0.23221900	-0.69284900

**INT-7**

E(RB3LYP) = -726.326932 Hartree

Sum of electronic and thermal Free Energies = -726.187395 Hartree

C	-1.26074200	-0.46064600	0.34588200

C	-1.60251100	0.80949400	-0.12713500

C	-2.82889400	1.07418200	-0.74033600

C	-3.72507200	0.00718100	-0.84671400

C	-3.39911700	-1.26350200	-0.33602100

C	-2.16926900	-1.51229200	0.28395800

C	1.26282000	-0.59650600	0.01028100

C	0.14674900	-0.10766200	0.95581400

C	-0.30488900	1.44209900	0.29555800

H	-3.08879000	2.06451500	-1.10910600

H	-4.69623400	0.15560600	-1.31653800

H	-4.12390800	-2.07047500	-0.43936000

H	-1.93294100	-2.49985700	0.67444800

O	1.13204300	-1.01101000	-1.13267200

O	0.06811200	2.59345200	0.36816800

O	0.31098200	-0.18757200	2.22652700

C	3.92966000	0.47667000	-1.16212100

C	3.60873700	-0.71555700	-0.26829400

H	3.09530200	0.66046600	-1.84377900

H	4.08879700	1.37522700	-0.55692600

H	4.83415400	0.28514700	-1.75485100

H	4.43960400	-0.93258600	0.41184100

H	3.39825200	-1.60177200	-0.87582300

O	2.49397900	-0.43950000	0.58108700

**INT-8**

E(RB3LYP) = -1106.276052 Hartree

Sum of electronic and thermal Free Energies = -1106.038252 Hartree

C	0.51890800	1.66443500	-0.40058800

C	-0.03392200	1.46780600	0.86853800

C	-0.88884300	2.39231000	1.46795900

C	-1.18266600	3.54035400	0.72879600

C	-0.64753000	3.73121700	-0.55972100

C	0.20149800	2.78971400	-1.15318100

C	2.72991700	0.37631800	-0.33400600

C	1.20909000	0.26274500	-0.53148300

C	0.55516100	0.09637100	1.01456600

H	-1.33236800	2.22218600	2.44561400

H	-1.85244500	4.29357400	1.13888900

H	-0.90810500	4.63769600	-1.10409800

H	0.59397000	2.94432900	-2.15524000

O	3.33074500	1.34259200	0.11073700

O	0.43483400	-0.79520000	1.82265600

O	0.77727100	-0.53761300	-1.47501700

C	4.98433300	-1.19586900	1.10650300

C	4.75244300	-0.84579200	-0.35798100

H	4.60021800	-0.39555000	1.74440500

H	4.46399600	-2.12458400	1.36175500

H	6.05590200	-1.32317700	1.30853300

H	5.14787000	-1.62430000	-1.01806900

H	5.22147700	0.11189700	-0.60467000

O	3.35424800	-0.78560600	-0.65498300

C	-4.37922600	0.43079200	-0.27265600

C	-4.80683400	-0.73947500	0.35847700

C	-3.95443100	-1.83601500	0.46281400

C	-2.65003400	-1.79038800	-0.06949400

C	-2.23200600	-0.61482500	-0.72862600

C	-3.08827400	0.47808800	-0.80969300

H	-5.03900000	1.29276800	-0.34046200

H	-5.80812600	-0.79808400	0.78201700

H	-4.29087900	-2.73792100	0.97180700

H	-1.22361500	-0.56060000	-1.14980900

H	-2.72940800	1.38575200	-1.28888300

C	-1.78651200	-2.94589400	0.07952900

H	-2.13386800	-3.85462400	0.55742200

N	-0.52832300	-2.94840000	-0.27943200

N	0.56420500	-2.88504200	-0.61983700

**TS-6**

E(RB3LYP) = -1106.244359 Hartree

Sum of electronic and thermal Free Energies = -1106.002103 Hartree

C	-0.05530100	1.33146100	-0.52457000

C	-0.31639200	1.16329200	0.83435400

C	-1.25143700	1.93598100	1.51365500

C	-1.96420500	2.87619200	0.75967100

C	-1.72528200	3.03229000	-0.61336100

C	-0.76238900	2.26045600	-1.27682800

C	2.40661900	0.71065300	-0.65853300

C	0.99100100	0.25248100	-0.77441200

C	0.63579800	0.02302100	1.10373800

H	-1.44573600	1.80227000	2.57469900

H	-2.72669900	3.48742600	1.23873700

H	-2.30171900	3.76924900	-1.16966700

H	-0.58052400	2.39058800	-2.34096200

O	2.77841000	1.82554900	-0.32335100

O	1.33933300	-0.20050200	2.08104300

O	0.71746800	-0.76320600	-1.56311300

C	4.69643600	-0.45418600	1.09474200

C	4.60550300	-0.10971300	-0.38701600

H	3.99892800	0.16557800	1.66384400

H	4.41823400	-1.50000000	1.25994800

H	5.71676800	-0.29342100	1.46839200

H	5.23878700	-0.76806400	-0.99155700

H	4.89157000	0.93108500	-0.56505300

O	3.27311400	-0.32767200	-0.85902800

C	-4.50741100	-0.70706500	-0.42302200

C	-4.14513200	-0.98336800	0.89696400

C	-2.82824800	-1.30327800	1.21223800

C	-1.83073300	-1.35457500	0.21844700

C	-2.20339100	-1.06439700	-1.10894900

C	-3.52408500	-0.74971400	-1.41471800

H	-5.53521100	-0.45418600	-0.67233100

H	-4.89194100	-0.94892200	1.68775200

H	-2.55533000	-1.51060200	2.24474800

H	-1.42519900	-1.04190400	-1.86306300

H	-3.78626000	-0.52098700	-2.44576500

C	-0.46096200	-1.65427100	0.64243800

H	-0.36160700	-2.11446000	1.62316100

N	0.36884300	-2.32742800	-0.25717800

N	1.04787200	-3.22950900	-0.45229500

**INT-9**

E(RB3LYP) = -1300.718913 Hartree

Sum of electronic and thermal Free Energies = -1300.478594 Hartree

C	1.97517200	0.96668700	1.73364300

C	1.20999900	-0.05144000	2.30591900

C	1.77264700	-0.98897300	3.17399700

C	3.13550600	-0.89317100	3.45384200

C	3.90735400	0.13238900	2.88183800

C	3.33438100	1.07275400	2.02045800

C	1.75245300	2.05966300	-0.54482300

C	1.12566700	1.85250900	0.84095000

C	-0.19247600	0.04064500	1.82495500

H	1.15118000	-1.76824600	3.60527800

H	3.60644300	-1.60884600	4.12234100

H	4.96630800	0.19704500	3.11767000

H	3.93445500	1.85863700	1.57462200

O	2.71360100	1.44265700	-0.97012100

O	-1.13967800	-0.59365400	2.27419400

O	0.96518600	3.10649700	1.49154200

C	0.15674300	2.55942100	-3.40264800

C	1.32268700	3.15634500	-2.63486200

H	0.03495400	1.50038100	-3.13365900

H	-0.77044400	3.09181500	-3.16522300

H	0.34450900	2.65520800	-4.47963600

H	1.42792600	4.23464300	-2.78531100

H	2.26754600	2.66369900	-2.87043700

O	1.07859900	3.00045700	-1.20674400

C	-3.86870800	3.25250200	0.16144500

C	-3.38622200	2.43392200	-0.86327400

C	-2.19528400	1.72035200	-0.70631300

C	-1.47608800	1.81687600	0.49683000

C	-1.97420500	2.62920100	1.52919200

C	-3.16016600	3.34642700	1.36119100

H	-4.79275000	3.80990800	0.02896200

H	-3.93741100	2.34617800	-1.79628500

H	-1.84797800	1.04985900	-1.49098000

H	-1.43049600	2.69239300	2.46675200

H	-3.53146700	3.97254500	2.16855400

C	-0.20578700	1.02940200	0.67763000

H	-0.04333600	0.41655100	-0.25252600

H	0.32750000	3.60742200	0.95260100

C	-0.71246300	-1.18674700	-1.67096700

O	0.16201500	-0.23033400	-1.92915300

O	-0.34883500	-2.13025600	-0.84070200

Cs	2.54424200	-1.71385500	-1.18315200

O	-1.89362100	-1.12626900	-2.15103200

Cs	-3.07392000	-2.07095500	0.32423200

**TS-7**

E(RB3LYP) = -1300.706038 Hartree

Sum of electronic and thermal Free Energies = -1300.469406 Hartree

C	2.01444700	0.84347100	1.66755700

C	1.28081900	-0.24829900	2.13132900

C	1.87941400	-1.26528100	2.87283000

C	3.24982600	-1.17404000	3.13304300

C	3.98979800	-0.07487900	2.66838900

C	3.37619400	0.94766700	1.93403000

C	1.68879500	2.15569500	-0.47029300

C	1.11306800	1.79659100	0.90471300

C	-0.14466400	-0.11158800	1.70439400

H	1.28023700	-2.09878400	3.22856400

H	3.74824400	-1.95011000	3.70851200

H	5.05190500	-0.01306700	2.89124800

H	3.95019000	1.79435600	1.57157900

O	2.61696300	1.57737700	-1.01133300

O	-1.05765700	-0.84353800	2.11116500

O	0.97411500	2.98659600	1.68211800

C	0.09314400	2.83793300	-3.22815400

C	1.21214000	3.46579100	-2.41420500

H	0.04805000	1.75934100	-3.03983600

H	-0.87269400	3.27161600	-2.95001400

H	0.26425400	3.02368700	-4.29564700

H	1.21527900	4.55735700	-2.47503400

H	2.19338600	3.08313500	-2.70091600

O	1.00386400	3.17136300	-1.00682300

C	-3.92058500	3.16510500	0.46402400

C	-3.43896900	2.46672000	-0.64626800

C	-2.23182400	1.76571900	-0.58030200

C	-1.48716500	1.74412300	0.61249200

C	-1.98993400	2.44059100	1.72773800

C	-3.19146400	3.14805500	1.65473700

H	-4.85700600	3.71436300	0.40377700

H	-4.00395300	2.46557100	-1.57551000

H	-1.88600400	1.19359500	-1.43648000

H	-1.43125700	2.41434600	2.65824800

H	-3.55901200	3.67998400	2.52892300

C	-0.20026500	0.97511600	0.72352700

H	-0.03165000	0.36893800	-0.46537300

H	0.27526500	3.50637000	1.24777700

C	-0.75790500	-1.14206700	-1.73132100

O	0.06951100	-0.05556800	-1.67852800

O	-0.34496900	-2.20288300	-1.13581200

Cs	2.56103600	-1.58514600	-1.16108000

O	-1.88607500	-1.01162700	-2.27799900

Cs	-3.06286200	-2.09054500	0.22414100

**INT-10**

E(RB3LYP) = -1300.730398 Hartree

Sum of electronic and thermal Free Energies = -1300.492546 Hartree

C	0.50427000	-2.11069700	-1.92677100

O	0.22074500	-0.99886400	-2.74623800

O	-0.31042800	-2.29255100	-0.96668200

Cs	-3.00013000	-1.26487700	-0.99881500

O	1.51908200	-2.75870700	-2.21009600

Cs	2.11083800	-2.50090000	0.84389300

C	-1.90128200	1.45598600	0.84297700

C	-1.54917100	0.57259800	1.86426800

C	-2.49466300	0.13027200	2.78360200

C	-3.81425800	0.59191800	2.65748800

C	-4.16603400	1.48233100	1.63470200

C	-3.20070500	1.92252900	0.71017700

C	-0.31283500	3.18479300	-0.08215000

C	-0.69740000	1.70476400	-0.04661100

C	-0.08914400	0.24176700	1.76200100

H	-2.19643600	-0.54796000	3.57806900

H	-4.57071800	0.27117100	3.36986300

H	-5.18731200	1.84801400	1.56433200

H	-3.46666600	2.61869600	-0.08144700

O	-0.45861400	3.97466600	0.82219600

O	0.48816700	-0.53977800	2.56533500

C	2.33235300	4.69686900	-0.64259900

C	0.97757200	4.76202000	-1.32913200

H	2.20878000	4.48808600	0.42310500

H	2.95243300	3.90912300	-1.07849800

H	2.84735600	5.65855700	-0.75109600

H	1.07065200	4.95756300	-2.39978100

H	0.33117800	5.51617800	-0.87404800

O	0.30306100	3.48111400	-1.25733800

C	4.59060800	1.07818000	-0.35711200

C	3.63261000	1.07254600	-1.37502800

C	2.27151400	1.03148600	-1.07472500

C	1.81589000	0.99427800	0.26280200

C	2.80256000	0.99999900	1.28015200

C	4.16146200	1.03934100	0.97481100

H	5.65012600	1.10898400	-0.59626200

H	3.94495700	1.07656100	-2.41623700

H	1.56132100	0.95548700	-1.88736900

H	2.47403000	0.96858600	2.31318900

H	4.89109200	1.05646200	1.78199700

C	0.40484600	0.92423000	0.62705600

H	-0.27716600	-0.37631800	-2.18193700

O	-1.06241600	1.27491400	-1.41122500

H	-0.52855300	1.83093400	-2.00619000

**TS-8**

E(RB3LYP) = -1300.712583 Hartree

Sum of electronic and thermal Free Energies = -1300.476912 Hartree

C	0.82662500	-1.49421900	-1.80782500

O	0.10852200	-0.62022100	-2.59897900

O	0.29333400	-1.83405800	-0.69620600

Cs	-2.61984300	-1.99240500	-1.08975500

O	1.93283100	-1.86901000	-2.24850900

Cs	3.07548300	-1.90764100	0.48967800

C	-2.05617700	0.80709000	1.32425600

C	-1.35882000	-0.15032200	2.07449200

C	-2.01080000	-1.08458800	2.86231500

C	-3.41581900	-1.05282900	2.89821500

C	-4.11560200	-0.08586500	2.16614000

C	-3.44352800	0.85965100	1.36977400

C	-1.46038600	2.90748900	-0.03848700

C	-1.05889800	1.60962500	0.55179200

C	0.10454300	0.07396200	1.84999300

H	-1.44085600	-1.81010500	3.43629100

H	-3.96094200	-1.76399300	3.51358600

H	-5.20125900	-0.05711300	2.22179500

H	-3.98162800	1.61044600	0.80504100

O	-2.48439500	3.49884500	0.25492700

O	1.01394900	-0.56022400	2.39138800

C	-0.28581200	5.77602700	-0.40631000

C	-0.77141000	4.73066600	-1.39845900

H	-0.87591600	5.72924600	0.51312100

H	0.76760400	5.60785400	-0.16049900

H	-0.38937400	6.77997900	-0.83396900

H	-0.20398100	4.76832400	-2.33123600

H	-1.83650200	4.84885200	-1.61236900

O	-0.53731300	3.40054200	-0.89392100

C	4.14830900	2.24904400	-0.44216400

C	3.14143900	1.96178600	-1.36999200

C	1.84716100	1.66591900	-0.94304300

C	1.53517600	1.64657100	0.42923200

C	2.55606400	1.93340900	1.35578600

C	3.84950900	2.23573200	0.92419700

H	5.15498100	2.48196500	-0.78004700

H	3.36601300	1.94704300	-2.43288100

H	1.08807700	1.38708700	-1.66244100

H	2.32787900	1.90920800	2.41684300

H	4.62167400	2.46681600	1.65442000

C	0.21610900	1.20339400	0.90679400

H	-0.57181700	-0.12837200	-2.03405300

O	-1.80046800	0.78849900	-1.40291600

H	-1.62192500	1.53504100	-1.99585600

**INT-11**

E(RB3LYP) = -920.366162 Hartree

Sum of electronic and thermal Free Energies = -920.150341 Hartree

C	2.14557700	0.38462800	0.07336100

C	2.47292800	-0.97374100	-0.07349700

C	3.78275800	-1.40873200	-0.15542700

C	4.80216900	-0.44534200	-0.08335500

C	4.48257600	0.90384900	0.06684300

C	3.14742700	1.34054600	0.14711300

C	0.00741300	1.83571900	0.16634100

C	0.66260700	0.50720200	0.10202200

C	1.20193300	-1.74887600	-0.11422800

H	4.00419500	-2.46566000	-0.27051200

H	5.84225500	-0.75152400	-0.14176300

H	5.28130200	1.63792300	0.12556500

H	2.90623200	2.38889300	0.27553500

O	0.50978300	2.81235300	0.69233300

O	1.06997600	-2.95056400	-0.24706600

C	-3.31057900	2.78450800	-0.95752500

C	-1.95111100	3.06783200	-0.34978000

H	-3.21403700	2.50003500	-2.00981600

H	-3.80526800	1.96596700	-0.42588600

H	-3.94106000	3.67784700	-0.89550900

H	-2.01878300	3.35588100	0.70405900

H	-1.41339000	3.86237500	-0.87702200

O	-1.18706800	1.84563400	-0.44692500

C	-4.01484100	-1.90124200	0.34187700

C	-3.16359800	-2.54476300	-0.55636300

C	-1.82560200	-2.16777500	-0.65178300

C	-1.32340800	-1.12084800	0.14017400

C	-2.18625600	-0.49403800	1.05710000

C	-3.51851700	-0.87857800	1.15543900

H	-5.05603300	-2.20147200	0.41836500

H	-3.54015200	-3.34713400	-1.18420300

H	-1.16473700	-2.67640600	-1.34278400

H	-1.79782600	0.28284800	1.70749700

H	-4.16900900	-0.38934500	1.87506900

C	0.07932200	-0.72361600	0.03413100

**INT-12**

E(RB3LYP) = -1300.318984 Hartree

Sum of electronic and thermal Free Energies = -1300.002976 Hartree

C	2.43425600	-0.65622800	0.62390000

C	3.23846300	0.26531700	-0.06725200

C	4.54455000	-0.01405000	-0.42579500

C	5.06714900	-1.27074900	-0.08507700

C	4.27710800	-2.19227300	0.60211900

C	2.95141700	-1.89926100	0.96903000

C	0.05416300	-0.76548500	1.61301400

C	1.09171800	-0.03943600	0.84257500

C	2.43629500	1.49742800	-0.29256000

H	5.13480500	0.72555600	-0.95846400

H	6.08709900	-1.52671600	-0.35574700

H	4.69271800	-3.16152300	0.86365600

H	2.34758000	-2.61854300	1.50714400

O	0.14445200	-1.94768900	1.90703800

O	2.80515100	2.53564600	-0.80656100

C	-3.34438700	0.13508700	2.28406200

C	-2.08070700	-0.61039000	2.66735800

H	-3.24345900	1.20600700	2.48314500

H	-3.56395900	-0.00345600	1.22183100

H	-4.18956500	-0.25155000	2.86441100

H	-2.12381100	-1.66459500	2.39074200

H	-1.87175600	-0.54155700	3.74054600

O	-0.97790900	0.01164700	1.96839900

C	-2.07211700	4.07113900	-0.27415700

C	-0.79535500	4.48652200	0.10523000

C	0.22243800	3.55322100	0.29156300

C	-0.03188100	2.18031900	0.12569200

C	-1.31893700	1.77612300	-0.26685700

C	-2.32731000	2.71079600	-0.46675600

H	-2.86103500	4.80237100	-0.42708500

H	-0.58654600	5.54255800	0.25050100

H	1.21610300	3.88231300	0.56905400

H	-1.52281000	0.72641600	-0.42486000

H	-3.31266300	2.37416300	-0.77746300

C	1.05151500	1.21025600	0.28653000

C	-4.23491800	-1.84405900	-1.01551500

C	-3.47053800	-2.70947700	-0.22923100

C	-2.08504600	-2.74629900	-0.35859700

C	-1.43388000	-1.91196900	-1.28571800

C	-2.21091300	-1.05194300	-2.08412100

C	-3.59518000	-1.01808700	-1.94331800

H	-5.31520400	-1.81672700	-0.91020400

H	-3.95550900	-3.35830000	0.49488100

H	-1.49305400	-3.40018900	0.27335300

H	-1.72769000	-0.40180400	-2.80795700

H	-4.17719900	-0.34466800	-2.56624800

C	0.01809400	-1.95021900	-1.35731800

H	0.61343300	-2.64670000	-0.78240000

N	0.69112800	-1.08150000	-2.05025800

N	1.27501200	-0.28387100	-2.62869000

**TS-19**

E(RB3LYP) = -1300.298958 Hartree

Sum of electronic and thermal Free Energies = -1299.977230 Hartree

C	2.19298400	-0.99089200	0.43429800

C	3.17873800	-0.12903500	-0.06254300

C	4.50879700	-0.50914000	-0.17667800

C	4.86049300	-1.80203600	0.22090600

C	3.88477400	-2.66819800	0.72529700

C	2.54349100	-2.27749800	0.83798700

C	-0.17532800	-0.71140100	1.37350800

C	0.85467000	-0.30227600	0.38214400

C	2.55607500	1.18353100	-0.37317200

H	5.24224300	0.19501900	-0.55791000

H	5.89178400	-2.13424200	0.14670600

H	4.17146400	-3.66695100	1.04327900

H	1.80213900	-2.94693900	1.25589000

O	-0.07400900	-1.69825100	2.08413300

O	3.13639800	2.19059900	-0.73897100

C	-3.51350500	0.59474100	1.90761700

C	-2.32442000	-0.26989200	2.27633700

H	-3.25424200	1.65616000	1.96026000

H	-3.84659600	0.36912600	0.89037100

H	-4.34255300	0.39923700	2.59641200

H	-2.53849500	-1.33544900	2.16764800

H	-1.98456100	-0.09325600	3.30209200

O	-1.25326700	0.08357500	1.37449800

C	-1.42708200	4.50894700	0.07792700

C	-0.10276100	4.54971100	0.51187100

C	0.70131800	3.41365100	0.43383400

C	0.19110400	2.20466600	-0.06836800

C	-1.14206300	2.18175400	-0.50818400

C	-1.94210100	3.31713200	-0.43624000

H	-2.05099300	5.39662000	0.13456800

H	0.31254100	5.47086600	0.91101900

H	1.73282200	3.46422700	0.75799400

H	-1.56235200	1.26088600	-0.88817400

H	-2.97121900	3.27032600	-0.78242300

C	1.06656100	1.01549100	-0.14503400

C	-4.12900600	-2.32742800	-0.73255800

C	-3.14631200	-3.09680500	-0.10694800

C	-1.79782600	-2.82798700	-0.32686200

C	-1.41472300	-1.76665400	-1.16346200

C	-2.40854600	-1.00685300	-1.80288600

C	-3.75406100	-1.28791800	-1.58786000

H	-5.17990700	-2.54340600	-0.56525400

H	-3.42892300	-3.91413100	0.54984400

H	-1.03723800	-3.41719900	0.17429900

H	-2.12412700	-0.21032400	-2.48332200

H	-4.51235600	-0.69771500	-2.09390300

C	0.01535100	-1.45081800	-1.27550700

H	0.70238800	-2.29349000	-1.22525500

N	0.39671300	-0.54095200	-2.23065800

N	0.86692600	0.52587800	-2.27562300

**INT-13**

E(RB3LYP) = -1300.347672 Hartree

Sum of electronic and thermal Free Energies = -1300.024280 Hartree

C	1.62229600	2.34319200	0.30273300

C	0.46715300	1.95787000	-0.38642400

C	-0.10285500	2.81753500	-1.32304700

C	0.50202600	4.05904400	-1.53897600

C	1.65236100	4.44624800	-0.83568700

C	2.22496100	3.58514700	0.09634900

C	2.07306900	1.27319300	1.21446300

C	-0.02942400	0.61061200	0.09401100

C	-0.50415500	-0.31304600	-1.01691700

H	-0.97903800	2.51829100	-1.88484000

H	0.07248300	4.73772400	-2.27054800

H	2.09827400	5.41770700	-1.02684800

H	3.12112700	3.84806600	0.64977900

O	2.98738600	1.30378200	2.00595900

O	-0.70811900	0.01500900	-2.16582500

C	3.49772000	-3.10330200	-0.85896500

C	3.05753100	-3.23170700	0.45888100

C	2.30591500	-2.21872600	1.05564200

C	1.98119600	-1.06941500	0.33104700

C	2.44499300	-0.93050200	-0.98325200

C	3.19199700	-1.94596400	-1.57701800

H	4.08016500	-3.89516400	-1.32117400

H	3.29857900	-4.12465400	1.02896000

H	1.95994900	-2.31744300	2.07730400

H	2.20931700	-0.03662500	-1.55367700

H	3.53628600	-1.82988900	-2.60069400

C	1.14643000	0.03208000	0.95047700

C	-0.88567700	-3.90199900	-0.79211100

C	-1.11723500	-2.56445200	-1.46506800

H	0.17487900	-4.03028300	-0.55549100

H	-1.45999900	-3.97387700	0.13703300

H	-1.19965600	-4.71369300	-1.45701000

H	-2.17282500	-2.38592000	-1.69400100

H	-0.54010500	-2.45681900	-2.38676100

O	-0.68453800	-1.54800000	-0.53378300

C	-5.11742200	-0.32382200	-0.04933400

C	-4.59179300	0.92924300	-0.36593400

C	-3.31411000	1.27874000	0.06922000

C	-2.54630000	0.37694300	0.81324100

C	-3.07925700	-0.87613200	1.13185000

C	-4.36008200	-1.22260500	0.70453900

H	-6.11399800	-0.59714800	-0.38410000

H	-5.17667500	1.63634100	-0.94702400

H	-2.90617300	2.25659800	-0.17399200

H	-2.48528500	-1.57262400	1.71297600

H	-4.76748800	-2.19644400	0.96135700

C	-1.13951600	0.75728000	1.20454200

H	-1.14450200	1.79988300	1.54548500

N	-0.63286200	-0.01998100	2.36965600

N	0.54113600	-0.38617500	2.24275500

**5a**

E(RB3LYP) = -1300.365179 Hartree

Sum of electronic and thermal Free Energies = -1300.038544 Hartree

C	0.19023500	2.87634500	0.03055600

C	-0.70284900	1.87751800	-0.36409000

C	-1.89939900	2.22537200	-0.98618100

C	-2.16636800	3.57707800	-1.21231700

C	-1.25971600	4.57508700	-0.82546700

C	-0.06813300	4.22903400	-0.19694000

C	1.37952000	2.29303900	0.67206100

C	-0.18574900	0.48403000	-0.01669300

C	0.18964200	-0.31203200	-1.26327300

H	-2.61600700	1.47023500	-1.28732500

H	-3.09554900	3.86011500	-1.69878500

H	-1.49463700	5.61820600	-1.01516700

H	0.65364500	4.97515300	0.12082600

O	2.38965700	2.86170700	1.03315900

O	0.37902900	0.17492400	-2.35681200

C	4.40242300	-1.88275900	0.09948900

C	3.48478900	-2.18307300	1.10743500

C	2.42909300	-1.31287900	1.37475100

C	2.28354600	-0.12760000	0.64673500

C	3.20890200	0.16995900	-0.36086600

C	4.25943500	-0.70484500	-0.63519400

H	5.22475300	-2.56078800	-0.11029100

H	3.58520100	-3.09951200	1.68223900

H	1.69567300	-1.57542300	2.13063500

H	3.10692000	1.08414400	-0.93529800

H	4.97106600	-0.45982800	-1.41829900

C	1.09530700	0.77832200	0.87211300

C	1.02939300	-3.83685900	-1.44718500

C	0.86108000	-2.44806300	-2.02753900

H	1.73490500	-3.81596400	-0.61187200

H	0.07150200	-4.22607100	-1.08824100

H	1.41576600	-4.51603900	-2.21465800

H	0.16344600	-2.42728400	-2.87067100

H	1.81087300	-2.01917000	-2.35923600

O	0.33371200	-1.61090900	-0.97246900

C	-4.72569700	-2.32361100	0.17660100

C	-3.96534800	-1.81727000	-0.87608700

C	-2.77533200	-1.13694000	-0.62160000

C	-2.32407600	-0.94637500	0.69475600

C	-3.10232700	-1.46004500	1.74899800

C	-4.28581500	-2.14071600	1.49116500

H	-5.65276500	-2.85335400	-0.02241700

H	-4.29481800	-1.95105200	-1.90256200

H	-2.20376300	-0.75638700	-1.46059000

H	-2.75993500	-1.31075200	2.76693000

H	-4.87214800	-2.52857000	2.31952600

C	-1.07520800	-0.23936600	0.99322700

N	-0.64166700	-0.10377200	2.20404400

N	0.49184800	0.67040500	2.21367200

H	1.12132900	0.44003000	2.97348800

**Supplementary References**

(1) Gaussian 16, Revision A.03,M. J. Frisch, G. W. Trucks, H. B. Schlegel, G. E. Scuseria, M. A. Robb, J. R. Cheeseman, G. Scalmani, V. Barone, G. A. Petersson, H. Nakatsuji, X. Li, M. Caricato, A. V. Marenich, J. Bloino, B. G. Janesko, R. Gomperts, B. Mennucci, H. P. Hratchian, J. V. Ortiz, A. F. Izmaylov, J. L. Sonnenberg, D. Williams-Young, F. Ding, F. Lipparini, F. Egidi, J. Goings, B. Peng, A. Petrone, T. Henderson, D. Ranasinghe, V. G. Zakrzewski, J. Gao, N. Rega, G. Zheng, W. Liang, M. Hada, M. Ehara, K. Toyota, R. Fukuda, J. Hasegawa, M. Ishida, T. Nakajima, Y. Honda, O. Kitao, H. Nakai, T. Vreven, K. Throssell, J. A. Montgomery, Jr., J. E. Peralta, F. Ogliaro, M. J. Bearpark, J. J. Heyd, E. N. Brothers, K. N. Kudin, V. N. Staroverov, T. A. Keith, R. Kobayashi, J. Normand, K. Raghavachari, A. P. Rendell, J. C. Burant, S. S. Iyengar, J. Tomasi, M. Cossi, J. M. Millam, M. Klene, C. Adamo, R. Cammi, J. W. Ochterski, R. L. Martin, K. Morokuma, O. Farkas, J. B. Foresman, and D. J. Fox, Gaussian, Inc., Wallingford CT, **2016**.

(2) a) S. Grimme, J. Antony, S. Ehrlich, H. Krieg, *J. Chem. Phys.*, **2010**, *132*, 154104-154123. b) Lee, C.; Yang, W.; Parr, R. G. *Phys. Rev. B*, **1988**, *37*, 785-789

(3) a) H. Puthoff, *Phys. Rev. A*, **1989**, *40*, 4857-4862; b) A. V. Marenich, C. J. Cramer, D. G. Truhlar, *J. Phys. Chem. B*, **2009**, *113*, 4538-4543.

(4) C. Y. Legault, CYLView, 1.0b, Université de Sherbrook, **2009**; www.cylview.org.
